# DNA/RNA-Based Next-Generation Sequencing Improves the Early Diagnosis and Management of Neoplastic Bile Duct Strictures: A 6-Year, Prospective, Multi-Institutional, Real-Time Study

**DOI:** 10.1053/j.gastro.2026.02.040

**Published:** 2026-03-27

**Authors:** Rohit Das, Jeffrey Kleinberger, Tarek Sawas, Abigail I. Wald, Robert Bubar, Harkirat Singh, Jennifer Chennat, Asif Khalid, Stephanie L. Romutis, Mordechai Rabinovitz, Anna Tavakkoli, Kimberly J. Fairley, Brian A. Boone, Savreet Sarkaria, John Nasr, Nikhil R. Thiruvengadam, Charles Gabbert, Sultan Mahmood, Ahmed A. Messallam, Mihajlo Gjeorgjievski, Markus Goldschmiedt, Shyam Thakkar, Thomas Tielleman, Mohannad Dugum, Jon Reich, Shailendra Singh, Randall E. Brand, Kevin McGrath, Kenneth Fasanella, Anne Marie Lennon, Rekha Reddy, Roshan Shrestha, Daniel J. Ellis, Sara A. Singhi, Terrence Lee, Peter S. Kay, Ryan D. Lee, Tiffany Y. Chua, Ibrahim Nassour, James L. Buxbaum, Joanna K. Law, Yasser Bhat, Prija A. Jamidar, Emily R. Jonica, Jeffrey J. Easler, Wasseem Skef, Hendrikus Dutch Vanderveldt, Anil K. Dasyam, David A. Geller, Amer H. Zureikat, Herbert J. Zeh, Patricio M. Polanco, J. Wallis Marsh, Melissa Hogg, Kenneth K. Lee, James F. Pingpank, M. Haroon A. Choudry, Abhinav Humar, Carl Schmidt, John Mansour, Geoffrey Nunns, Melanie Ongchin, Alessandro Paniccia, Sigfred Lajara, Sara E. Monaco, N. Paul Ohori, Liron Pantanowitz, James M. Mitchell, Katelyn Smith, Nuha Shaker, Anju H. Singhi, Dennis Hsu, Anwaar Saeed, Ibrahim H. Sahin, Janie Zhang, Vikram Gorantla, John Rhee, Marina N. Nikiforova, Nisa Kubiliun, Adam Slivka, Aatur D. Singhi

**Affiliations:** 1 Department of Medicine, University of Pittsburgh Medical Center, Pittsburgh, Pennsylvania; 2 Department of Pathology, University of Pittsburgh Medical Center, Pittsburgh, Pennsylvania; 3 Department of Pathology, Geisinger Medical Center, Danville, Pennsylvania; 4 Department of Medicine, University of Texas Southwestern, Dallas, Texas; 5 VA Pittsburgh Healthcare System, Pittsburgh, Pennsylvania; 6 Department of Medicine, West Virginia University Health Sciences Center, Morgantown, West Virginia; 7 Department of Surgery, West Virginia University Health Sciences Center, Morgantown, West Virginia; 8 Vandalia Health Mon Medical Center, Morgantown, West Virginia; 9 Department of Medicine, Division of Gastroenterology and Hepatology, Loma Linda University Medical Center, Loma Linda, California; 10 Piedmont Healthcare, Marietta, Georgia; 11 Gastro Health, Grandview Medical Center, Birmingham, Alabama; 12 Digestive Health Center, Essentia Health-Duluth Clinic, Duluth, Minnesota; 13 Eugene Gastroenterology Consultants, P.C., Springfield, Oregon; 14 Division of Gastroenterology, Hepatology, and Nutrition, University of Florida, Gainesville, Florida; 15 Department of Surgery, University of Florida, Gainesville, Florida; 16 University of Southern California, Keck Medicine, Digestive Health Institute, Los Angeles, California; 17 Virginia Mason Franciscan Health, Center for Digestive Health, Seattle, Washington; 18 Department of Gastroenterology, Palo Alto Medical Foundation (PAMF), Mountain View, California; 19 Division of Digestive Diseases and Advanced Endoscopy, Yale University School of Medicine, New Haven, Connecticut; 20 Division of Gastroenterology and Hepatology, Oregon Health and Science University, Portland, Oregon; 21 Department of Gastroenterology and Hepatology, Indiana University Health Medical Center, Indianapolis, Indiana; 22 Baylor College of Medicine, Gastroenterology and Hepatology, Michael E. DeBakey VA Medical Center, Houston, Texas; 23 Department of Radiology, University of Pittsburgh Medical Center, Pittsburgh, Pennsylvania; 24 Department of Surgery, University of Pittsburgh Medical Center, Pittsburgh, Pennsylvania; 25 Department of Surgery, University of Texas Southwestern, Dallas, Texas; 26 Department of Surgery, NorthShore University Health System, Evanston, Illinois; 27 Department of Pathology, Memorial Sloan Kettering Cancer Center, New York, New York; 28 Department of Pathology, University of Texas Southwestern, Dallas, Texas; 29 Department of Medicine, University of Michigan, Ann Harbor, Michigan

**Keywords:** Cholangiocarcinoma, Pancreatic Cancer, Next-Generation Sequencing, Biliary Stricture, Early Diagnosis

## Abstract

**BACKGROUND & AIMS:**

The distinction between benign and neoplastic bile duct strictures remains challenging. Pathologic assessment of endoscopic retrograde cholangiopancreatography (ERCP)-obtained specimens has limited sensitivity, particularly among patients with primary sclerosing cholangitis (PSC). Next-generation sequencing of bile duct specimens provides a promising diagnostic approach, but a prospective, multi-institutional, and comprehensive DNA/RNA analysis is lacking.

**METHODS:**

A 6-year, prospective, multi-institutional study was conducted using BiliSeq version 2 (28 cancer-associated genes and 167 fusion genes) and BiliSeq version 3 (161 cancer-associated genes and 763 fusion genes) for 2908 ERCP-obtained brushings, biopsies, and bile from 2116 patients at 28 medical institutions. Molecular results were compared with clinical, imaging, and pathologic parameters including diagnostic pathology and/or at least 1-year follow-up.

**RESULTS:**

BiliSeqV2/V3 testing was performed for 2865 (99%) specimens from 2080 (98%) patients. Based on follow-up from 1979 (95%) patients, BiliSeq version 2/version 3 demonstrated 82% sensitivity and 98% specificity for a neoplastic stricture. In comparison, pathologic assessment had a sensitivity of 44% and a specificity of 99%. Combining BiliSeq version 2/version 3 testing with pathologic assessment improved the sensitivity to 88% and maintained a high specificity of 97%. High-risk populations, such as Hispanic, germline carrier, and PSC patients, also showed improvement in sensitivity with BiliSeq version 2/version 3 (74% to 86%) compared with pathologic assessment (26% to 50%). Further, actionable molecular alterations were identified in 20% of BiliSeq version 3–positive neoplasms and modified patient management in 30% of these cases.

**CONCLUSIONS:**

Applying BiliSeq version 2/version V3 testing to ERCP-obtained specimens improved the diagnostic evaluation of bile duct strictures, achieving higher sensitivity, especially for PSC, and maintained high specificity compared with traditional methods. This study highlights the importance of next-generation sequencing for precise diagnosis and therapeutic intervention.

## Graphical Abstract

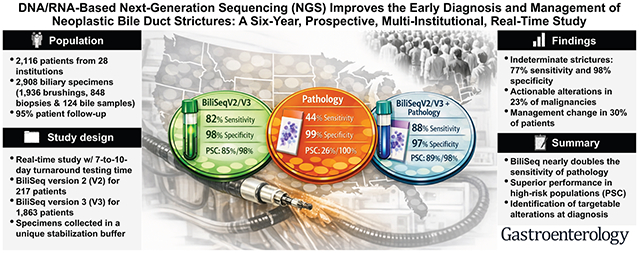

**D**espite advancements in imaging, pathology, and cytogenetic techniques, distinguishing between benign and neoplastic bile duct strictures remains challenging.^1,2^ Two key tools used to evaluate bile duct strictures are magnetic resonance cholangiopancreatography (MRCP) and endoscopic retrograde cholangiopancreatography (ERCP). Although MRCP and ERCP are sensitive modalities that often define the location and nature of a disease process involving the biliary tree, both techniques can be diagnostically inaccurate and, thus, require pathologic assessment of corresponding biliary specimens.^3^ Bile duct brushings and biopsies serve as the preferred specimens for pathologic evaluation, and the preoperative diagnosis of at least “suspicious for malignancy” is associated with ~99% specificity for neoplasia.^4,5^ The sensitivity of pathology, however, is between 8% and 67%.^6–9^ Hence, cytogenetic testing of bile duct brushings using fluorescence in situ hybridization (FISH) is an important adjunct.^10,11^ Although aneuploidy has been described in 80% of neoplastic strictures, the sensitivity of FISH combined with pathology is only 50% to 63%.^12–14^ Notably, FISH on cytologically benign specimens is associated with a high false-positive rate, especially among patients with primary sclerosing cholangitis (PSC).^15,16^ Chromosomal abnormalities do not indicate the location or type of neoplasm nor provide therapeutically relevant data.

In addition to chromosomal abnormalities, neoplasms arising from or secondarily involving the biliary tree are characterized by recurrent gene point mutations, small insertions and deletions, frameshift mutations, and copy number alterations. These alterations can be detected in not only bile duct brushings but also in biopsies and bile. ^17–19^ We previously reported the incorporation of a 28-gene DNA-based next-generation sequencing (NGS) panel (BiliSeq version 1 [BiliSeqV1]) for the preoperative evaluation of 252 patients with a bile duct stricture. ^19^ The sensitivity and specificity of BiliSeqV1 for neoplastic strictures were 73% and 100%, respectively. In combination with pathology, the sensitivity of BiliSeqV1 increased to 83%. Multiple investigators have independently published similar results using analogous DNA-based NGS panels.^17,18,20,21^ However, these studies, including BiliSeqV1, have largely been single-institutional experiences or retrospective analyses. Furthermore, a large, comprehensive analysis of high-risk populations, such as PSC, Hispanic, and germline carrier patients, has not been performed. Finally, numerous cancer-associated fusion genes have been identified as key diagnostic biomarkers and potential drug targets for precision medicine–directed therapies, but their status has not been fully investigated with respect to biliary specimens.

To address these limitations, we conducted a 6-year prospective, real-time, multi-institutional analysis of 2 expanded NGS panels (BiliSeq version 2 [BiliSeqV2] and BiliSeq version 3 [BiliSeqV3]) to detect neoplastic strictures from ERCP specimens. BiliSeqV2 combines BiliSeqV1 with RNA testing of 167 fusion genes, whereas BiliSeqV3 includes an expanded DNA- and RNA-based panel of 161 genes and 763 fusion genes. Similar to BiliSeqV1, BiliSeqV2 and BiliSeqV3 were performed within a Clinical Laboratory Improvement Amendments–certified and College of American Pathologists–accredited clinical laboratory and within a 7- to 10-day turnaround that promptly provides genomic data to the patient’s clinical team. To facilitate rapid reporting of results, all ERCP-obtained specimens were collected using a previously published nucleic acid lysis/stabilization buffer rather than using cytologic smears/slides, alcohol fixatives (eg, CytoLyte), or formalin-fixed paraffin-embedded tissue, which may decrease overall yields and quality of DNA and RNA. The objectives of this study were to (1) evaluate the diagnostic performance of combined DNA/RNA-based NGS testing in the assessment of neoplastic strictures from a diverse patient population, (2) determine the accuracy of BiliSeqV2/V3 among high-risk patients (eg, PSC, etc.), and (3) identify oncologic actionable molecular alterations at the time of diagnosis to improve patient management.

## Materials and Methods

### Study Population

Study approval was obtained from the authors’ respective institutional review boards (overarching institutional review board number STUDY19030333) and the study design is outlined in Supplementary Figure 1. Prospective BiliSeqV2 and BiliSeqV3 testing were performed between January 2018 and September 2024 and consisted of 2865 biliary specimens obtained by ERCP from 28 independent medical institutions (see the Supplementary Data for study cohort description). Briefly, biliary specimens (brushings, biopsies, and bile) were submitted using a provided collection vial containing a nucleic acid lysis/stabilization buffer. Within 24 to 48 hours of the ERCP procedure, the vial was shipped to the University of Pittsburgh Medical Center (UPMC) Molecular & Genomics Pathology (MGP) laboratory for nucleic acid extraction/isolation, DNA/RNA library creation, sequencing, and reporting of results. Medical records were reviewed for patient demographics, ERCP findings, serum carbohydrate antigen 19-9 (CA19-9) levels, and pathologic assessment of brushings, biopsies, and/or bile specimens.^19,22^ A separate analysis was performed for 359 repeat ERCPs from 269 patients for BiliSeqV2/V3. The etiology of a bile duct stricture was based on diagnostic pathology from surgically resected specimens or biopsy specimens (either autopsy, open or laparoscopic surgical resection/biopsy, percutaneous needle, or ERCP-obtained biliary biopsy) and/or clinical criteria after ≥12 months of follow-up. As the reported specificity of bile duct brushings is <100%, the findings from pathological assessment of brushing specimens were not considered diagnostic pathology. Furthermore, a negative ERCP-obtained biopsy was not considered supportive of benign etiology. A stricture was designated as a benign cholangiopathy if a repeat imaging or ERCP at ≥12 months of follow-up documented the resolution or stability of prior ductal abnormalities. In contrast, a clinical diagnosis of neoplasia was defined by the combination of a mass on radiographic imaging in the absence of acute and/or chronic cholangiopathy, and clinical or radiographic progression after ≥12 months of follow-up, or death clinically and radiographically determined to be due to a neoplasm involving the bile duct. For surgical resection and biopsy specimens, diagnostic pathologic diagnoses were rendered based on standard histomorphologic criteria and reported immunohistochemical stains.

### BiliSeq Version 2 Testing

DNA/RNA-based NGS testing was performed prospectively as part of clinical care and within a 7- to 10-day turnaround in the UPMC MGP laboratory (see Supplementary Materials and Methods for assay validation). The DNA component of BiliSeqV2 was previously described and consisted of a 28-gene panel (BiliSeqV1).^19^ RNA-based testing included 167 fusion genes (Supplementary Table 1). Briefly, 10 ng messenger RNA was reverse transcribed into complementary DNA, subject to multiplex polymerase chain reaction, and sequenced on an Ion S5 XL System (Thermo Fisher Scientific) with data analyzed using Torrent Suite Software v5.12 and Variant Explorer v2 (see Supplementary Materials and Methods).^23,24^ More than 50 fusion-specific reads that cross the fusion breakpoint are required to make a positive fusion call.

### BiliSeq Version 3 Testing

Amplification-based targeted DNA-based NGS was performed with AmpliSeq primers for 161 genes (Supplementary Table 2) to include single nucleotide variants, small insertions and deletions (indels), loss of heterozygosity, and copy number alterations (see Supplementary Data for assay validation). Amplicons were barcoded, ligated with specific adapters, purified, and sequenced on an Ion GeneStudio S5 Prime System (Thermo Fisher Scientific) with data analyzed using Variant Explorer v2 (see Supplementary Materials and Methods).^25,26^ Each variant was prioritized according to the 2017 Association for Molecular Pathology/American Society of Clinical Oncology/College of American Pathologists joint consensus guidelines for interpretation of sequence variants in cancer using a tier-based system. Tier I, Tier II, and Tier III variants were identified; however, only Tier I and Tier II variants were used for subsequent analysis. The minimum depth of coverage for testing was 1000x. For each alteration, a variable allele frequency (VAF) was calculated based on the number of reads of the mutant allele vs the wild-type allele and reported as a percentage. A 1.5% VAF was used as a cutoff criterion for an alteration. Germline variants were predicted based on a VAF of approximately 45% to 55% and extensive literature and searchable database review to include but not limited to ClinVar, BRCA Exchange, ARUP, cBioportal, and others. RNA-based testing was performed similarly to that described for BiliSeqV2 but consisted of 763 fusion genes (Supplementary Table 3).

### Classification of Actionable Molecular Alterations

Therapeutically relevant alterations were defined using a combination of OncoKB classification, European Society of Medical Oncology Scale for Clinical Actionability of Molecular Targets, American Society of Clinical Oncology guidelines, National Comprehensive Cancer Network Clinical Practice Guidelines in Oncology, Food and Drug Administration–approved regimens, and those that contributed to patient accrual in registered clinical trials at the time of detection.^27–32^

### Statistical Analysis

Chi-squared analysis or Fisher’s exact tests were used to compare categorical data, and the Mann-Whitney *U* test was used to compare continuous variables. Sensitivity, specificity, positive predictive value, and negative predictive value were calculated with 95% Wilson confidence intervals using standard 2×2 contingency tables for cases with follow-up. All statistical analyses were performed using the SPSS Statistical software, V.29 (IBM Corp), and statistical significance was defined as a *P* value of <.05.

## Results

### Prospective, Real-Time, Multi-institutional BiliSeq Version 2/Version 3 Testing of 2080 Patients

In total, prospective molecular testing was attempted for 2908 biliary specimens (1936 bile duct brushings, 848 bile duct biopsies, and 124 collected bile samples) from 2116 patients undergoing an ERCP for a bile duct stricture and obtained from 28 institutions over 6 years. Specimens were submitted at the discretion of the performing gastroenterologist based on clinical judgment. Sufficient nucleic acid for combined DNA and RNA testing was identified in 2865 (99%) specimens (1900 brushings, 841 biopsies, and 124 bile specimens) from 2080 (98%) patients (Supplementary Table 4). Failed specimens included 36 bile duct brushings and 7 bile duct biopsies. Among the 2080 patients, 759 (36%) patients had multiple, concurrent bile duct specimens submitted for molecular testing: 658 (of 759, 87%) patients with brushing and biopsy specimens, 72 (9%) patients with brushing and bile specimens, 3 (<1%) patients with biopsy and bile specimens, and 26 (3%) patients with all 3 specimens. Genomic alterations were observed in 845 (44%) brushings, 433 (51%) biopsies, and 36 (29%) bile specimens. For paired specimens, 17 (of 759, 2%) patients had discrepant molecular results that consisted of 15 patients with brushing and biopsy specimens (6 with negative brushing and positive biopsy; 9 with positive brushing and negative biopsy) and 2 patients with brushing and bile specimens (positive brushing and negative bile). Repeat testing was performed for 269 patients, which includes an additional 325 bile duct brushings, 195 bile duct biopsies, and 27 collected bile specimens.

**Figure 1.**
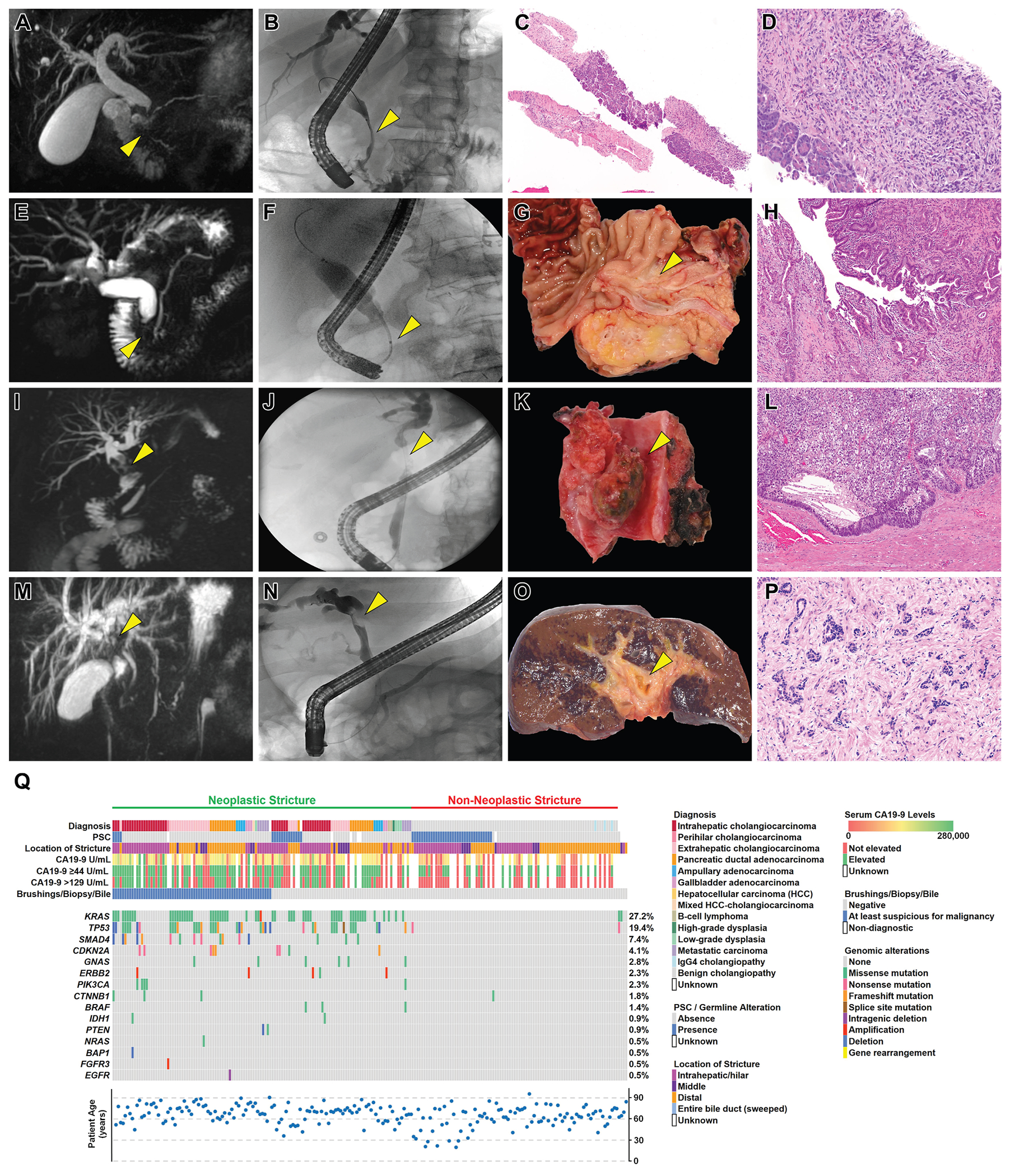
Representative patients with bile duct strictures evaluated by BiliSeqV2/V3 testing. (*A–D*) A patient with an indeterminate distal bile duct stricture (*arrowheads*) on MRCP (*A*) and ERCP (*B*). Pathologic assessment showed atypical cells and BiliSeqV2/V3 was negative for genomic alterations. Follow-up biopsy (*C, D*) of the pancreas was characterized by ductitis and venulitis, diagnostic for autoimmune pancreatitis, type 1. (*E–H*) A patient with a distal stricture (*arrowheads*) on MRCP (*E*) and ERCP (*F*). ERCP-obtained brushings and biopsy were positive for *KRAS* and *TP53* mutations, but pathologically negative for neoplasia. Surgical resection (*G, arrowhead*) revealed a moderately differentiated extrahepatic cholangiocarcinoma (*H*). (*I–L*) A middle bile duct stricture (*arrowheads*) on MRCP (*I*) and ERCP (*J*). Pathology was suspicious for malignancy and BiliSeqV2/V3 detected a *TP53* mutation. Resection of the upper to middle bile duct (*K*) revealed a polypoid mass (*arrowhead*), consistent with a poorly differentiated extrahepatic cholangiocarcinoma with clear cell features (*L*). (*M–P*) A patient with an intrahepatic/ hilar stricture (*arrowheads*) on imaging MRCP (*M*) and ERCP (*N*). Brushings showed rare ductal cells, but BiliSeqV2/V3 identified alterations in *ARID1A*, *ATM*, *CDKN2A*, and *MDM2*. Resection (*O*) revealed an ill-defined mass in the liver hilum, consistent with a poorly differentiated intrahepatic cholangiocarcinoma (*P*). (*Q*) Oncoplot of 217 BiliSeqV2 patients organized by final diagnosis, showing alterations in 73% of neoplastic strictures vs 53% detection by pathologic assessment.

**Figure 2.**
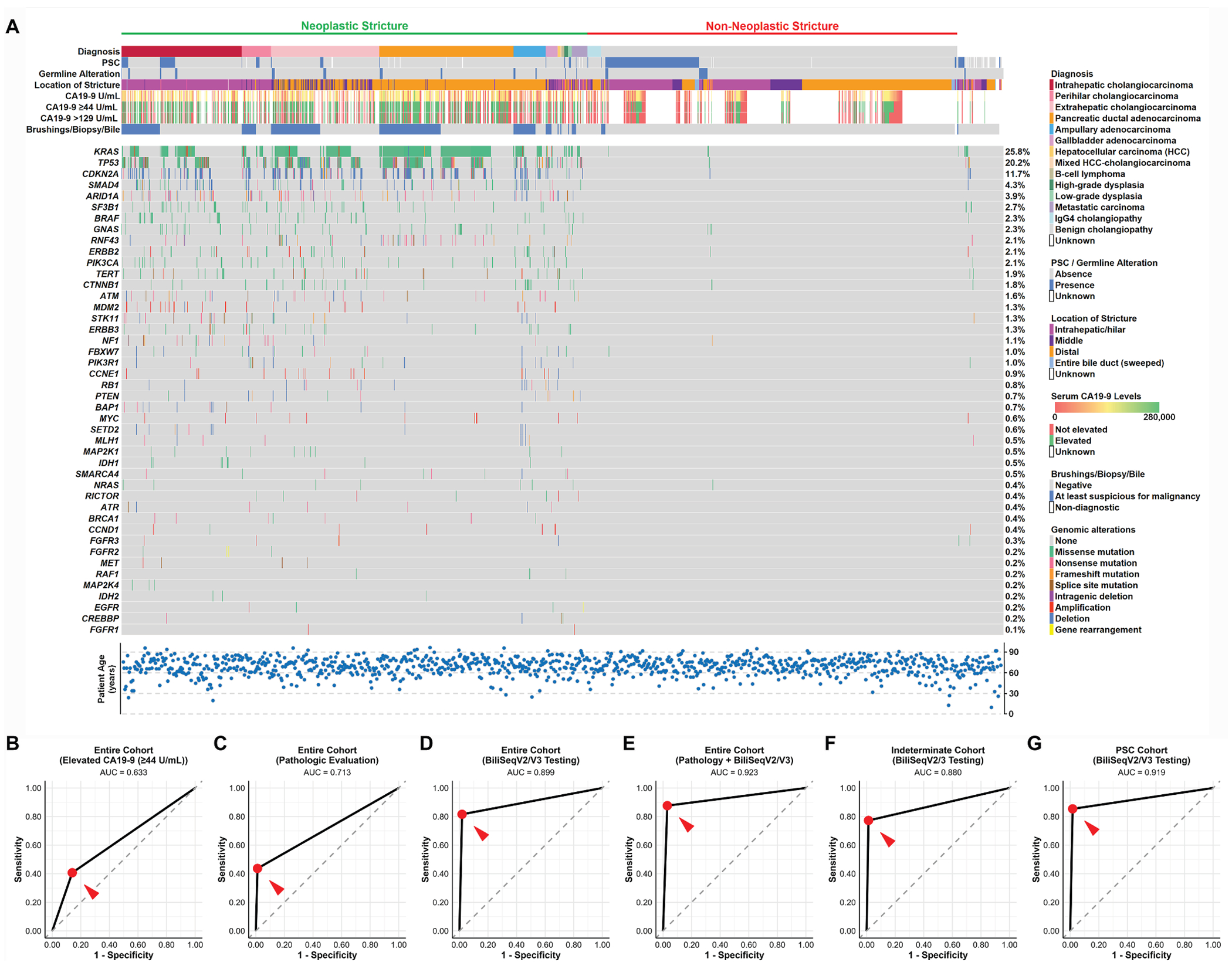
(*A*) Oncoplot of 1863 BiliSeqV3 patients organized by final diagnosis. BiliSeqV3 detected genomic alterations in 83% of neoplastic strictures, with *KRAS, TP53, CDKN2A, SMAD4, ERBB2, GNAS, ARID1A*, and *SF3B1* most prevalent. Pathologic assessment was suspicious for neoplasm/malignancy in only 43% of cases. (*B–G*) Receiver operating characteristic curves comparing serum CA19-9 (≥44 U/mL), pathologic evaluation, BiliSeqV2/V3, and combined pathology/BiliSeqV2/V3 for the entire cohort (*B–E*), indeterminate strictures (*F*), and PSC patients (*G*). The *red dot* and *red arrow* indicate the operational threshold of each assay. AUC, area under the curve.

BiliSeqV2, a DNA/RNA-based panel of 28 genes and 167 fusion genes, was used to evaluate this study’s first 217 (10%) patients (Figure 1). An alteration was found in 71 (43%) brushings, 66 (46%) biopsies, and 2 (40%) bile specimens from 96 (44%) patients and included *KRAS* (n = 59, 27%), *TP53* (n = 42, 19%), *SMAD4* (n = 16, 7%), *CDKN2A* (n = 9, 4%), *GNAS* (n = 6, 3%), and others. The remaining 1863 (90%) patients were evaluated using BiliSeqV3, a DNA/RNA-based panel of 161 genes and 763 fusion genes (Figure 2). BiliSeqV3 detected an alteration in 774 (45%) brushings, 367 (53%) biopsies, and 34 (29%) bile specimens from 847 (45%) patients and, similar to BiliSeqV2, included *KRAS* (n = 480, 26%), *TP53* (n = 377, 20%), *CDKN2A* (n = 217, 12%), and *SMAD4* (n = 81, 4%), but also *ARID1A* (n = 72, 4%), *SF3B1* (n = 49, 3%), and other genes. Within the combined patient cohort (BiliSeqV2/V3), variant allele frequencies (VAFs) for individual genes varied widely and ranged between 1.0% and 85.6% (Supplementary Figure 2); however, a VAF of 1.5% was used as a cutoff for a positive result. The RNA-based panel to BiliSeqV2 did not identify any alterations; however, BiliSeqV3 detected *MET* exon 14 (*MET*ex14) skipping (n = 2), *EGFR::VOPP1* (n = 1), *EML4::ALK* (n = 2), *FGFR2::BICC1* (n = 2), *KANK1::NTRK3* (n = 1), and *TMPRSS2::ERG* (n = 1) alterations. Further, BiliSeqV3 identified germline alterations for 69 (4%) patients (Supplementary Table 5). Most of these germline alterations are known to be involved in homologous recombination (n = 56, 78%).

### Clinicopathologic Correlation and Follow-Up for 1979 Patients

In comparison with a negative result, detectable genomic alterations (Table 1) based on BiliSeqV2 (Supplementary Table 6) and BiliSeqV3 (Supplementary Table 7) were associated with increased median patient age (71.0 years vs 64.0 years, *P* < .001), identified less often among Hispanic patients (29% vs 71%, *P* < .001), less frequent for patients with a PSC history (24% vs 76%, *P* < .001), found to correlate with elevated serum CA19-9 (≥44 U/mL: 66% vs 34%, *P* < .001; >129 U/mL: 71% vs 29%, *P* < .001), and at least “suspicious for malignancy” on preoperative pathology (84% vs 16%, *P* < .001). Besides older median age, no statistically significant differences were seen between BiliSeqV2 and BiliseqV3. Of note, no Hispanic patients nor detectable germline alterations were represented within the patient cohort for BiliSeqV2.

Follow-up was available for 1979 (95%) patients (Table 2) and included 1108 (56%) neoplastic strictures and 871 (44%) benign strictures. For the 1108 neoplastic strictures, 1087 (98%) were malignant and 21 (2%) were dysplastic (9 high grade and 12 low grade). An alteration by BiliSeqV2/V3 had 82% sensitivity and 98% specificity for a neoplastic stricture (Figure 2 and Table 3). Separately, BiliSeqV2 (Supplementary Table 8) and BiliSeqV3 (Supplementary Table 9) had similarly high specificities, but the sensitivity was 10% higher for BiliSeqV3 than BiliSeqV2. Between BiliSeqV2 and BiliSeqV3, 16 false-positive cases were identified and consisted of alterations involving *KRAS* (n = 4), *TP53* (n = 3), *GNAS* (n = 2), *CTNNB1* (n = 2), *CDKN2A* (n = 1), *ERBB2* (n = 1), *FBXW7* (n = 1), *NOTCH2* (n = 1), and *NRAS* (n = 1). Variant allele frequencies for the false-positive cases ranged between 1.5% and 30%, and 1 (6%) false-positive cases harbored genomic variants in more than 1 gene.

Serum CA19-9 data at the time of clinical presentation was available for 918 (46%) patients and an elevated CA19-9 with a cutoff of ≥44 U/mL and with a cutoff of >129 U/mL had lower sensitivities (69% and 54%) and lower specificities (54% and 78%). A preoperative pathologic diagnosis of at least “suspicious for malignancy” also had a lower sensitivity of 44%, but a high specificity of 99% (*P* < .001 by McNemar’s test, Supplementary Data and Supplementary Figures 3 to 23). However, adding BiliSeqV2/V3 improved the sensitivity of preoperative pathology to 88% and maintained a high specificity of 97% (*P* < .001 by McNemar’s test, Supplementary Data). BiliSeqV2/V3 and pathology were further evaluated based on specimen type. Among 1815 bile duct brushings, 833 bile duct biopsies, and 107 bile collections, the sensitivity and specificity of BiliSeqV2/V3 for a neoplastic stricture ranged from 73% to 85% and 97% to 99%, respectively. The specificity of pathology for these specimen types was analogous to BiliseqV2/V3 but had lower sensitivities between 17% and 47%. However, combining BiliSeqV2/V3 with pathology increased the sensitivities to between 73% and 88%, while the specificities remained between 97% and 99%.

### Subanalysis Based on Indeterminate Stricture, Demographics, History, Stricture Location, and Repeat Endoscopic Retrograde Cholangiopancreatography Bile Duct Testing

A total of 548 patients presented with indeterminate biliary strictures, defined as strictures that remain uncharacterized as benign or malignant following cross-sectional imaging and ERCP with tissue sampling, and follow-up. For these diagnostically challenging cases, BiliSeqV2/V3 demonstrated 77% sensitivity and 98% specificity for detecting a neoplastic stricture (Figure 2 and Table 4). In comparison, elevated CA19-9 at cutoffs of ≥44 U/mL and >129 U/mL had lower sensitivities of 61% and 44%, and specificities of 53% and 79%, respectively. Among multiple racial and ethnic groups (Supplementary Table 10), such as Hispanic and Asian patients, and germline carriers (Supplementary Table 11), BiliSeqV2/V3 was once again superior in performance as compared with elevated CA19-9 and pathologic assessment. The sensitivity of BiliSeqV2/V3 was on the order of 36% to 44% higher than pathology. For 327 PSC patients with follow-up that included 89 neoplastic cases, the sensitivity of pathology was 26%, which contrasted with a sensitivity of 46% for 1631 non-PSC patients with follow-up (Supplementary Table 12 and Table 4). BiliSeqV2/V3 was superior in sensitivity at 85% and its combination with pathology attained 89% sensitivity and 98% specificity. A subanalysis of BiliSeqV3 revealed a higher prevalence of *GNAS* and *MDM2* alterations in patients with PSC as compared with non-PSC patients (*P* = .002 and *P* < .001, respectively), and a lower *TP53* prevalence (*P* = .011) (Supplementary Table 13).

Analogous to PSC and non-PSC patients, differences in pathologic assessment were identified based on stricture location (Supplementary Tables 14 and 15). The sensitivity of pathology identifying a neoplastic stricture within the intrahepatic/hilar region was 36%, whereas the middle and distal locations were 51% and 48%, respectively. In comparison, BiliSeqV2/V3 has sensitivities of 80%, 87%, and 82% within the intrahepatic/hilar, middle, and distal locations, respectively. The sensitivities of detecting neoplastic strictures involving the intrahepatic/hilar, middle, and distal bile duct were further improved to 86%, 93%, and 87%, respectively, by combining BiliSeqV2/V3 and pathology. Using BiliSeqV3 data alone, *KRAS*, *TP53*, and *RNF43* alterations were more prevalent in the distal bile duct as compared with the intrahepatic/hilar bile duct (*P* < .001, *P* = .002, and *P* = .014, respectively) (Supplementary Table 16). Conversely, alterations involving the *TERT* promoter, *STK11*, *NF1*, and *BAP1* were more likely to be identified in the intrahepatic/hilar bile duct (*P* = .028, *P* = .017, *P* = .001, and *P* = .001, respectively).

Repeat ERCP with brushings, biopsies, and/or bile for BiliSeqV2/V3 and pathologic assessment was performed for 269 (of 2080, 13%) patients with follow-up for 260 (97%) patients (Table 4). Among the 918 biliary specimens (547 bile duct brushings, 323 bile duct biopsies, and 48 collected bile samples) from 260 patients with follow-up, an ERCP with specimen collection was repeated 1 time for 194 (75%) patients, 2 times for 54 (21%) patients, 3 times for 8 (3%) patients, 4 times for 2 (1%) patients, and 5 times for 2 (1%) patients. The initial diagnostic performance of BiliSeqV2/V3 for these 260 patients yielded 72% sensitivity and 97% specificity for a neoplastic stricture (Supplementary Table 17). Pathologic assessment, however, was associated with only 14% sensitivity and 99% specificity, whereas the combination of BiliSeqV2/V3 and pathology achieved a sensitivity and specificity of 75% and 97%, respectively. The sensitivities of BiliSeqV2/V3, pathology, and both diagnostic modalities increased to 83%, 30%, and 90%, respectively, with the next ERCP specimen for all 260 patients. On factoring all subsequent repeat ERCP specimens, sensitivities and specificities of BiliSeqV2/V3, pathology, and both diagnostic modalities were 85%, 33%, and 93%, respectively, and 93%, 98%, and 92%, respectively.

### Therapeutically Relevant Genomic Alterations

**Figure 3.**
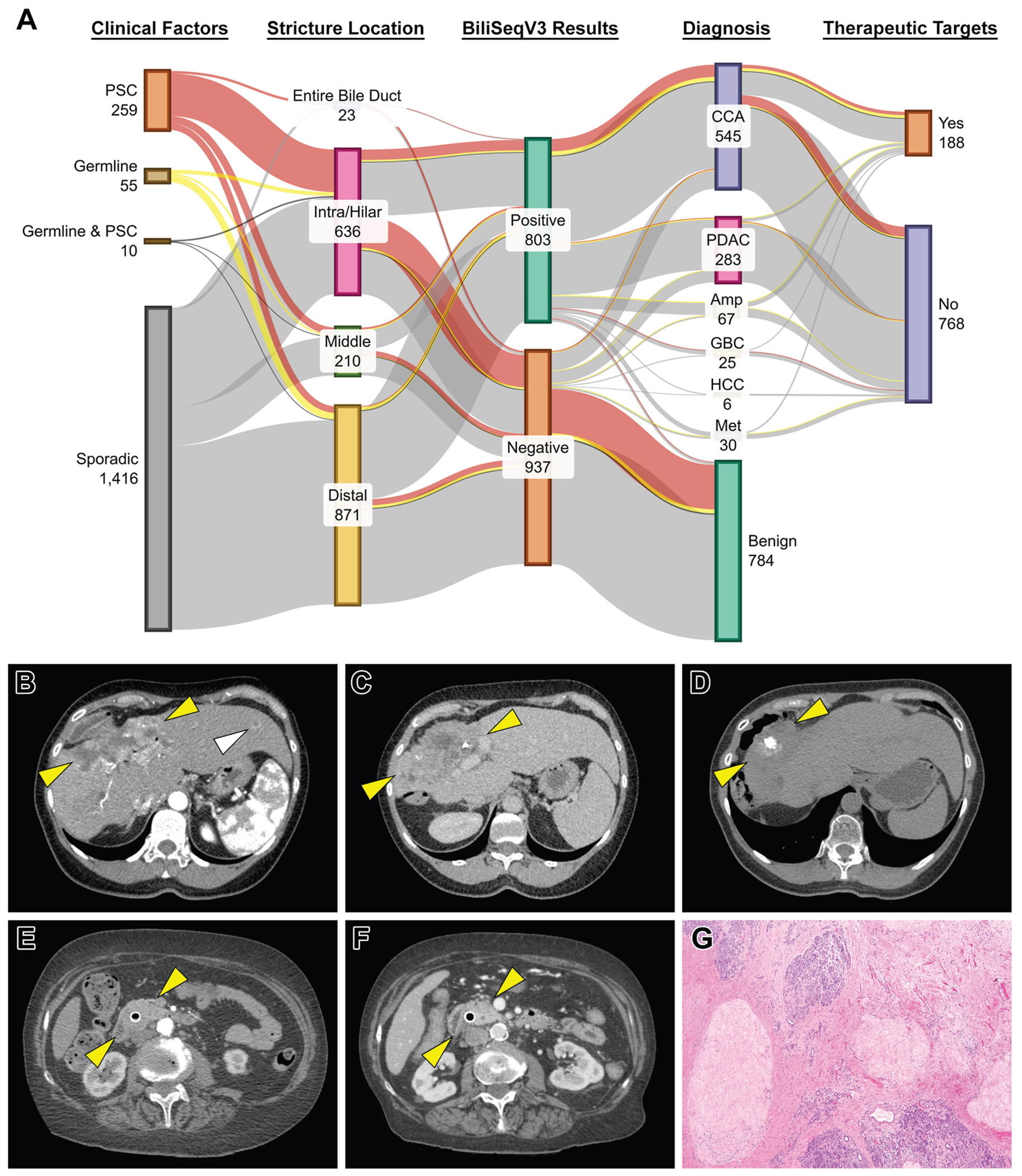
(*A*) Alluvial plot of 1740 of 1766 (99%) patients with BiliSeqV3 testing and clinicopathologic follow-up, excluding dysplasia, lymphoma, and mixed neoplastic cases. The plot displays germline/PSC status, anatomic stricture location, BiliSeqV3 results, follow-up diagnosis, and therapeutically relevant alterations. Of 826 neoplastic strictures, 182 (23%) harbored actionable molecular alterations, and these data modified management in 56 (30%) patients. (*B–D*) A 61-year-old woman with a 10.6-cm intrahepatic mass (*yellow arrowheads*), single satellite lesion (*white arrowhead*), and bile duct stricture on computed tomography (CT) (*B*). ERCP specimens were pathologically negative, but BiliSeqV3 identified an *FGFR2*::*BICC1* fusion. The patient was treated with pemigatinib, with tumor reduction to 6 cm at 8 months (*C*) and 4.6 cm with increased calcification at 20 months (*D*). (*E–G*) A 60-year-old woman with a 3.4-cm pancreatic head mass (*arrowheads*) on CT (*E*). Bile duct brushings and biopsy were positive for adenocarcinoma, and BiliSeqV3 detected alterations in *KRAS*, *TP53*, *SMAD4*, *RB1*, and *BRCA2*. Based on the *BRCA2* deletion, the patient received FOLFIRINOX. At 6 months (*F*), the mass decreased to 2 cm. Pancreaticoduodenectomy showed complete pathologic response with a 1.4-cm fibrotic scar (*G*).

Focusing on BiliSeqV3 and those patients with detailed clinicopathologic features and follow-up, 188 of 812 (23%) BiliSeqV3-positive malignancies harbored targetable alterations (Figure 3 and Supplementary Table 18). These neoplasms consisted of 137 of 458 (30%) cholangiocarcinomas (CCAs), 15 of 228 (7%) pancreatic ductal adenocarcinomas, 24 of 58 (41%) ampullary adenocarcinomas, 4 of 20 (20%) gallbladder carcinomas, 7 metastatic cancers, and 1 hepatocellular carcinoma. Most actionable molecular alterations involved the mitogen-activated protein kinase (MAPK) pathway (n = 106, 56%) and included *ERBB2* (n = 35), *ERBB3* (n = 18), *BRAF* V600E (n = 12), *KRAS* G12C (n = 12), *MAP2K1* (n = 8), *FGFR2* (n = 4), *RAF1* (n = 3), *EGFR* (n = 3), *MAPK1* (n = 3), *ALK* (n = 2), *FGFR1* (n = 2), *MAP2K4* (n = 2), *MET* exon 14 skipping (n = 1), and *NTRK3* (n = 1). Other relevant alterations were associated with DNA repair (n = 75, 40%): homologous recombination (*ATM* [n = 35], *BRCA2* [n = 11], *BRCA1* [n = 8], *ATR* [n = 10], *FANCA* [n = 1], *FANCD2* [n = 1], *MRE11* [n = 1], and *NBN* [n = 1]) and mismatch repair (*MLH1* [n = 7]). Finally, *IDH1*/2 mutations were identified in 11 cases. For 56 of 188 (30%) patients, these alterations were used to modify patient management, and 37 of 56 (66%) patients exhibited disease stability by radiographic imaging or measurable response by Response Evaluation Criteria in Solid Tumors (RECIST) or on surgical pathology assessment had a partial to complete response (Figure 3). Of note, for 1 patient, a *TMPRSS2*::*ERG* fusion was detected in the patient’s bile duct specimens. As the presence of a *TMRPSS2* gene fusion is highly specific for prostate cancer, its identification prompted further examination of the patient’s prostate, and he was subsequently diagnosed with metastatic prostatic adenocarcinoma.

## Discussion

The integration of expanded NGS panels, such as BiliSeqV2 and BiliSeqV3, with traditional pathologic assessment represents an advancement in the diagnostic evaluation of bile duct strictures. Based on a multi-institutional, prospectively collected, and real-time genomic reporting of 2865 biliary specimens from 2080 patients with a bile duct stricture and follow-up from 1979 patients, BiliSeqV2/V3 testing achieved a sensitivity of 82% and a specificity of 98% for a neoplastic stricture. Both CA19-9 (≥44 U/mL) and pathologic assessment demonstrated lower sensitivities of 69% and 44%, respectively. However, combining BiliSeqV2/V3 with the pathologic assessment of biliary specimens improved the sensitivity to 88% and maintained a high specificity of 97%.

The clinical utility of BiliSeqV2/V3 extends beyond improved sensitivity. Although the positive predictive value is high for both pathology and BiliSeqV2/V3, the clinical value of incorporating, parallel NGS-based testing of biliary specimens lies in the detection of patients who would have otherwise been missed at initial presentation. Given that a delayed diagnosis for neoplasms arising or involving the biliary system can be associated with disease progression and loss of surgical candidacy, this improvement may have significant implications on patient outcome. Moreover, multiple societies and guidelines recommend routine NGS-based testing, as approximately one-third of patients can harbor alterations that can be targeted by approved or investigational therapies.^33,34^ Thus, integrating BiliSeqV2/ V3 and similar assays to the assessment of bile duct strictures may not only expedite an early diagnosis but also subsequent management.

Multiple studies have investigated the application of NGS to biliary specimens, but the reported accuracy seems to vary widely. This variation in diagnostic performance may be attributed to the location of the stricture, the specimen type, the method of specimen collection, and the sequencing detection strategy. One of the earliest studies was by Dudley et al,^17^ who evaluated a cohort of 73 bile duct brushing specimens using a 39-gene DNA-based NGS panel. The sensitivity and specificity for a neoplastic stricture were 68% and 97%, respectively. Of note, the authors extracted DNA from CytoLyte-preserved brushing specimens. As an alcohol fixative may result in nucleic acid degradation, it is not unexpected that 8 of 73 (11%) brushing specimens failed NGS testing. BiliSeqV2/V3 uses a dedicated biliary specimen collected in a buffer that simultaneously lyses cells and stabilizes nucleic acid for subsequent analysis. The failure rate of employing this workflow as described herein is ~1%. Nevertheless, Harbhajanka et al^21^ reported 93% sensitivity and 100% specificity using a cohort of 94 CytoLyte-preserved brushing specimens and a 52-gene DNA-based NGS panel. However, the authors of this study did not report their assay failure rate and 79% of their neoplastic cohort were pancreatic ductal adenocarcinomas. Thus, the study was heavily biased toward distal strictures, which are easier to sample than intrahepatic/hilar strictures.

We previously published the results of BiliSeqV1, a 28-gene DNA-based NGS panel, which evaluated 252 patients with a bile duct stricture.^19^ BiliSeqV1 achieved 73% sensitivity and 100% specificity for a neoplastic stricture. The sensitivity and specificity of BiliSeqV3 were 83% and 98%, respectively. The increased sensitivity and decreased specificity of BiliSeqV3 as compared with BiliSeqV1 is likely due to a few key differences between the 2 NGS panels: expansion from 28 to 161 DNA-based genes, the inclusion of RNA testing, and an increased limit of detection (LOD), specifically a VAF of 1.5% instead of 3.0%. The LOD of an NGS assay is a key factor in its sensitivity, but it can also lead to an increased false-positive rate. The Bilemut assay by Arechederra et al^18^ is a 52-gene DNA-based panel designed for ERCP-obtained bile specimens and has an LOD of 0.15%. Based on a 68-patient cohort, the Bilemut assay had a sensitivity of 96%, but a poor specificity of 69%. Although BiliSeqV2/V3 has a lower LOD, the assay yielded 73% sensitivity and 97% specificity for neoplastic strictures using 107 bile specimens. These findings suggest future studies are required to define the optimal LOD for biliary specimens that may be dependent on specimen type: brushings, biopsies, and bile.

The diagnostic evaluation of indeterminate biliary strictures represents a particularly challenging scenario where BiliSeqV2/V3 provides added clinical value. Among 548 patients with indeterminate strictures in our study, defined as strictures remaining uncharacterized after cross-sectional imaging and ERCP with tissue sampling, BiliSeqV2/V3 achieved 77% sensitivity and 98% specificity. This finding is consistent with recent literature highlighting the role of NGS in this population. Meijering et al^35^ reported that NGS of biliary specimens led to faster and better management in almost 1 of 10 cases with indeterminate strictures, and suggested that NGS could be cost-effective because it can be performed on previously obtained samples without requiring additional procedures. Similarly, a recent study from the University of Texas Southwestern Medical Center demonstrated that BiliSeqV1 and pathologic evaluation enhanced malignant biliary stricture detection sensitivity, leading to management changes in 75% of cases when pathology test results were negative.^36^

Another critical aspect of this study is the evaluation of high-risk patients, such as those with PSC. PSC is an autoimmune condition, associated with a 15% lifetime incidence of CCA or 400 times higher than the general population.^37,38^ Unfortunately, among patients with PSC, the sensitivity of pathology for neoplastic strictures is suboptimal and is 5% to 40%.^39^ The poor sensitivity is due to the inflammatory background that often characterizes these specimens and, thus, the distinction between neoplastic cells and inflammatory atypia can be difficult. Within this study of 327 patients with PSC with neoplastic follow-up for 89 patients, pathologic assessment had a 26% sensitivity and 100% specificity, but BiliSeqV2/V3 had an 85% sensitivity and a 98% specificity. In a case-control study of 20 patients with PSC with and without CCA, Kamp et al^20^ found DNA-based NGS of a 14-gene panel to be associated with a similar sensitivity of 75%, but a specificity of 85%. The lower specificity is likely due to the reported LOD of 0.1%. Currently, FISH testing is widely used to evaluate PSC specimens. In a meta-analysis of 8 studies, the pooled sensitivity and specificity of FISH for CCA in patients with PSC were 68% and 70%, respectively.^16^ As a result, FISH testing is frequently ordered for patients with PSC with an equivocal pathologic diagnosis whereby the sensitivity and specificity increase to 80% and 98%, respectively.^40,41^ In comparison, combining BiliSeqV2/V3 and pathology of patients with PSC attained 89% sensitivity and 98% specificity. Importantly, FISH testing has inherent limitations that NGS overcomes. FISH detects chromosomal abnormalities but does not provide information about the specific genes involved, the location or type of neoplasm, or therapeutically actionable targets.

In addition to PSC, the incidence of CCA is higher among Hispanic and Asian populations (2.8 to 3.3 cases per 100,000) as compared with the non-Hispanic White and Black populations (2.1 cases per 100,000).^42,43^ Stratifying based on race and ethnicity, the detection of an alteration by BiliSeqV2/V3 was less often seen in Hispanic patients. Nonetheless, BiliSeqV2/V3 was superior to conventional diagnostic modalities and showed similar sensitivities and specificities for a neoplastic stricture regardless of patient background. Neoplasms arising from or secondarily involving the biliary tree are well documented to be associated with germline predisposition syndromes. Considering the expanded DNA coverage of BiliSeqV3, it is not surprising that 4% of patients harbored a pathogenic germline alteration. Moreover, most germline alterations, such as *BRCA2*, *BRCA1*, and *PALB2*, involve the homologous recombination repair pathway.^44^ Germline alterations associated with homologous recombination repair have been linked to improved survival with platinum- and PARP inhibitor–based chemotherapy and an increased hereditary risk for patients’ family members.^45–47^ In other words, BiliSeqV3 could potentially impact treatment decisions and guide cancer prevention strategies.

With the identification of pathologic germline alterations, the detection of actionable molecular alterations is another application of BiliSeqV2/V3. BiliSeqV2/V3 was superior to conventional diagnostic modalities; however, patients frequently have locally advanced and/or metastatic disease at clinical presentation. In fact, 2% of neoplastic strictures described within this study were solely dysplastic. Excluding patients with PSC, BiliSeqV2/V3 represents a modality for early diagnosis and not necessarily early detection of neoplastic strictures. Thus, at the time of diagnosis, it is imperative to optimize patient management and, among patients with locally advanced pancreatobiliary neoplasms, longer overall survival has been reported for patients who have actionable molecular alterations and who have received matched therapy as compared with patients who received unmatched therapy or those without an actionable molecular alteration.^48^ Herein, BiliSeqV3 identified 23% of patients with an actionable molecular alteration and 66% of patients receiving a matched therapy exhibited at least disease stability by RECIST criteria.

We acknowledge that there are several limitations to this study. Follow-up and diagnostic pathology follow-up were unavailable for all patients but, for the remaining patients with reported follow-up, at least 1 year of close clinicoradiographic follow-up was required to determine the etiology for each bile duct stricture. Identical specimens for BiliSeqV2/V3 and pathologic assessment were not evaluated, and this may explain the discordant diagnostic performance between these 2 modalities. However, considering the 38% difference in sensitivity between BiliSeqV2/V3 and pathologic assessment and that >2000 bile duct specimens were evaluated, it is unlikely that differences in specimens would explain the disparate results. This study does not address the ideal gene panel for the evaluation of bile duct strictures. Although BiliSeqV2 and BiliSeqV3 are DNA/RNA-based NGS panels, the number of genes evaluated by BiliSeqV3 is greater than BiliSeqV2. The sensitivity of BiliSeqV3 was 10% higher than BiliSeqV2 and this finding suggests the improved sensitivity for BiliSeqV3 is due to the expanded number of genes. Besides gene panels, the optimal approach of integrating NGS-based testing to the evaluation of bile duct strictures remains unclear. To minimize repeat sampling, BiliSeq is routinely performed in parallel to pathologic assessment. A positive BiliSeq result hastens the referral process for appropriate surgical and/or oncological subspecialists. A negative BiliSeq result provides additional reassurance of a negative pathological assessment. As none of these diagnostic modalities have perfect sensitivity, repeat testing may be necessary for a subset of patients.^49^ Indeed, repeat testing of 260 patients as described herein did improve the sensitivities for BiliSeqV2/V3, pathologic assessment, and their combination from 72%, 14%, and 75%, respectively, to 85%, 33%, and 93%, respectively. Another limitation is that a direct head-to-head comparison between BiliSeqV2/V3 and FISH testing was not performed, as FISH data were not systemically collected across participating institutions and, in our experience, often not ordered within incorporation of BiliSeqV2/V3. Nevertheless, Dudley et al^17^ reported that NGS-based testing of bile duct specimens had a higher sensitivity and higher specificity than paired FISH analysis. This study also lacks a comparison between BiliSeqV2/V3 of ERCP-obtained specimens and endoscopic ultrasound (EUS)-guided fine-needle aspiration (FNA) of bile duct strictures. Although EUS-FNA may be more effective for distal lesions, ERCP-obtained specimens are preferred for proximal lesions and small lesions (<1.0 cm).^50–54^ In the setting of bile duct wall thickening, EUS-FNA is associated with a low sensitivity for detecting neoplasia.^55^ Because ERCP is performed for both diagnosis and therapeutic stent placement, BiliSeqV2/V3 may obviate the need for EUS-FNA in select cases. A positive BiliSeqV2/V3 result can provide therapeutically relevant genomic alterations, but the true clinical benefit of matching patients with specific therapies needs to be evaluated as part of a randomized clinical trial. Nevertheless, we showed that 66% of patients receiving matched therapy exhibited at least disease stability by RECIST criteria. BiliSeqV2/V3 was also useful in identifying a previously undiagnosed, metastatic prostate cancer for 1 patient. Finally, although a formal cost-effectiveness analysis was beyond the scope of this study, several factors support the economic value of BiliSeqV2/ V3. In our repeat testing cohort, 24% of patients required 2 or more additional ERCPs before diagnosis, representing substantial procedural costs. The improved first-pass sensitivity of BiliSeqV2/V3 could reduce repeat procedures. Moreover, BiliSeqV2/V3 is approximately $1000 less than a 4-probe FISH assay and 5 times less than solid tumor NGS panels.^56^

In summary, the application of expanded DNA/RNA-based NGS panels, such as BiliSeqV2 and BiliSeqV3, with pathologic assessment of ERCP-obtained specimens, significantly improved the diagnostic evaluation of bile duct strictures. Among 2865 prospectively collected, ERCP-obtained biliary specimens from 2080 patients at 28 different medical institutions, BiliSeqV2/V3 demonstrated a sensitivity of 82% and specificity of 98% for a neoplastic stricture, surpassing CA19-9 and pathology alone. Combining BiliSeqV2/V3 with pathology increased sensitivity to 88% while maintaining specificity at 97%. Similarly, in high-risk populations like those with PSC, BiliSeqV2/V3 showed superior sensitivity (85%) over pathology. Ethnic variations in cholangiocarcinoma incidence were observed, with Hispanic patients showing fewer alterations, yet BiliSeqV2/V3 maintained high accuracy across racial and ethnic groups. BiliSeqV3 also identified germline alterations in 4% of patients, which can influence cancer prevention and treatment strategies. In fact, BiliSeqV3 detected actionable molecular alterations in 23% of patients, aiding targeted therapies with 66% achieving at least disease stability. Overall, DNA/RNA-based testing enhanced diagnostic accuracy, supported early diagnosis, identified actionable molecular alterations, and influenced treatment decisions and outcomes.

## Supplementary Material

1

2

Note: To access the supplementary material accompanying this article, visit the online version of *Gastroenterology* at www.gastrojournal.org , and at https://doi.org/10.1053/j.gastro.2026.02.040.

## Figures and Tables

**Table 1.** Clinical and Pathologic Findings of 2080 Patients With a Bile Duct Stricture and Associated BiliSeqV2/V3 and Prediagnostic Pathology

Patient and bile duct characteristics	Total (n = 2080)	BiliSeqV2/V3		*P*	Prediagnostic pathology^a^		*P*
		Negative (n = 1137, 55%)	Positive (n = 943, 45%)		Negative (n = 1570, 76%)	Positive^a^ (n = 496, 24%)	
Gender							
Female	916 (44)	512 (56)	404 (44)	.339	684 (75)	226 (25)	.466
Male	1164 (56)	625 (54)	539 (46)		886 (76)	270 (23)	

Median age (range), *y*	68.0 (8–97)	64.0 (8–96)	71.0 (13–97)	< .001	66.0 (8–96)	71.0 (24–97)	< .001

Racial representation (n = 2006)		n = 1085	n = 921		n = 1507	n=488	
White	1603 (80)	836 (52)	767 (48)	< .001	1183 (74)	413 (26)	.013
Black	204 (10)	119 (58)	85 (42)		160 (78)	41 (20)	
Hispanic	127 (6)	90 (71)	37 (29)		108 (85)	18 (14)	
Asian	72 (4)	40 (56)	32 (44)		56 (78)	16 (22)	

History of PSC (n = 2030)	341 (17)	260 (76)	81 (24)	< .001	312 (91)	24 (7)	< .001

CA19-9 ≥44 U/mL (n = 924)	577 (62)	199 (34)	378 (66)	< .001	339 (59)	238 (41)	< .001

CA19-9 >129 U/mL (n = 924)	411 (44)	119 (29)	292 (71)	< .001	227 (55)	184 (45)	< .001

Location of stricture (n = 2064)		n = 1125	n = 939		n = 1558	n = 496	
Intrahepatic/hilar	774 (38)	425 (55)	349 (45)	.026	615 (79)	155 (20)	.002
Middle	251 (12)	137 (55)	114 (45)		183 (73)	68 (27)	
Distal	1014 (49)	542 (53)	472 (47)		740 (73)	271 (27)	
Entire bile duct (sweep)	25 (1)	21 (84)	4 (16)		20 (80)	2 (8)	

Detection of germline alteration (n = 1863)	69 (4)	31 (45)	38 (55)	.233	48 (70)	21 (30)	.160

Positive prediagnostic pathology (n = 2066)^a^	496 (24)	79 (16)	417 (84)	< .001			

Neoplastic follow-up (n = 1979)	1108 (56)	204 (18)	904 (82)	< .001	624 (56)	484 (44)	< .001

NOTE. Values are n (%) unless otherwise indicated.

a Prediagnostic pathology was based on pathologic assessment of biliary brushings, biopsies, and/or bile, and was categorized as negative (benign or atypical) and positive (at least suspicious of a neoplastic process was identified).

**Table 2.** Follow-up From 1979 Patients Who Underwent BiliSeqV2/V3 Testing and Prediagnostic Pathologic Evaluation

Patient and bile duct characteristics	Total (n = 1979)	BiliSeqV2/V3		*P*	Prediagnostic pathology^a^		*P*
		Negative (n = 1059, 54%)	Positive (n = 920, 46%)		Negative (n = 1480, 75%)	Positive^a^ (n = 494, 25%)	
Clinical and diagnostic pathology follow-up							
Intrahepatic cholangiocarcinoma (PSC, n = 55)	294	55 (19)	239 (81)	< .001	190 (65)	104 (35)	< .001
Perihilar cholangiocarcinoma (PSC, n = 9)	62	13 (21)	49 (79)		32 (52)	30 (48)	
Extrahepatic cholangiocarcinoma (PSC, n = 14)	260	35 (13)	225 (87)		137 (53)	123 (47)	
Pancreatic ductal adenocarcinoma (PSC, n = 2)	305	61 (20)	244 (80)		167 (55)	138 (45)	
Ampullary adenocarcinoma	75	11 (15)	64 (85)		24 (32)	51 (68)	
Gallbladder adenocarcinoma (PSC, n = 1)	30	6 (20)	24 (80)		15 (50)	15 (50)	
HCC (PSC, n = 1)	8	2 (25)	6 (75)		5 (62)	3 (38)	
Mixed HCC-cholangiocarcinoma	3	2 (67)	1 (33)		1 (33)	2 (67)	
B-cell lymphoma	8	3 (38)	5 (62)		7 (88)	1 (12)	
High-grade biliary epithelial dysplasia (PSC, n = 2)	9	1 (11)	8 (89)		9 (100)	0 (0)	
Low-grade biliary epithelial dysplasia (PSC, n = 4)	12	2 (17)	10 (83)		9 (75)	3 (25)	
Metastatic colonic adenocarcinoma	18	4 (22)	14 (78)		12 (67)	6 (33)	
Metastatic non–small cell lung carcinoma	6	2 (33)	4 (67)		5 (83)	1 (17)	
Metastatic endometrial carcinoma	2	0 (0)	2 (100)		1 (50)	1 (50)	
Metastatic pancreatic ductal adenocarcinoma	2	0 (0)	2 (100)		1 (50)	1 (50)	
Metastatic esophageal adenocarcinoma	1	0 (0)	1 (100)		0 (0)	1 (100)	
Metastatic gastric adenocarcinoma	2	2 (100)	0 (0)		1 (50)	1 (50)	
Metastatic renal cell carcinoma	1	1 (100)	0 (0)		1 (100)	0 (0)	
Metastatic prostatic adenocarcinoma	2	0 (0)	2 (100)		2 (100)	0 (0)	
Metastatic appendiceal adenocarcinoma	1	0 (0)	1 (100)		0 (0)	1 (100)	
Metastatic ileal neuroendocrine tumor	2	2 (100)	0 (0)		1 (50)	1 (50)	
Metastatic breast carcinoma	2	1 (50)	1 (50)		1 (50)	1 (50)	
Cancer of unknown primary (PSC, n = 1)	3	1 (33)	2 (67)		3 (100)	0 (0)	
Benign cholangiopathy (PSC, n = 237)	837	821 (98)	16 (2)		822 (98)	10 (1)	
IgG4 cholangiopathy (PSC, n = 1)	34	34 (100)	0 (0)		34 (100)	0 (0)	

NOTE. Values are n (%) unless otherwise indicated.

HCC, hepatocellular carcinoma; Ig, immunoglobulin.

a Prediagnostic pathology was based on pathologic assessment of biliary brushings, biopsies, and/or bile, and was categorized as negative (benign or atypical) and positive (at least suspicious of a neoplastic process was identified)

**Table 3.** Diagnostic Performance of BiliSeqV2 and BiliSeqV3 Compared With CA19-9 Elevation and Pathologic Assessment

Parameter	Sensitivity, % (95% CI)	Specificity, % (95% CI)	Positive predictive value, % (95% CI)	Negative predictive value, % (95% CI)	Accuracy, % (95% CI)
Patients with BiliseqV2 testing (n = 213)					
CA19-9 ≥44 U/mL (n = 149)	73 (0.63–0.81)	47 (0.32–0.62)	75 (0.65–0.83)	44 (0.30–0.59)	64 (0.56–0.72)
CA19-9 >129 U/mL (n = 149)	52 (0.42–0.62)	87 (0.74–0.95)	90 (0.79–0.96)	46 (0.35–0.56)	63 (0.55–0.71)
Pathologic assessment	53 (0.44–0.62)	100 (0.96–1.00)	100 (0.95–1.00)	60 (0.51–0.68)	72 (0.66–0.78)
BiliSeqV2 testing	73 (0.64–0.81)	98 (0.92–1.00)	98 (0.93–1.00)	71 (0.62–0.79)	83 (0.77–0.88)
Pathologic assessment and BiliSeqV2 testing	86 (0.78–0.91)	98 (0.92–1.00)	98 (0.94–1.00)	83 (0.74–0.89)	91 (0.86–0.94)

Patients with BiliSeqV3 testing (n = 1766)					
CA19-9 ≥44 U/mL (n = 769)	69 (0.64–0.72)	56 (0.49–0.62)	80 (0.76–0.83)	41 (0.36–0.47)	65 (0.61–0.68)
CA19-9 >129 U/mL (n = 769)	54 (0.50–0.58)	76 (0.70–0.81)	85 (0.81–0.88)	40 (0.35–0.44)	60 (0.57–0.64)
Pathologic assessment	42 (0.39–0.46)	99 (0.98–0.99)	98 (0.96–0.99)	58 (0.55–0.60)	67 (0.65–0.70)
BiliSeqV3 testing	83 (0.80–0.85)	98 (0.97–0.99)	98 (0.97–0.99)	82 (0.79–0.84)	90 (0.88–0.91)
Pathologic assessment and BiliSeqV3 testing	88 (0.86–0.90)	97 (0.95–0.98)	97 (0.96–0.98)	86 (0.84–0.89)	92 (0.91–0.93)

All patients (n = 1979)					
CA19-9 ≥44 U/mL (n = 918)	69 (0.65–0.73)	54 (0.48–0.60)	79 (0.75–0.82)	42 (0.36–0.47)	65 (0.62–0.68)
CA19-9 >129 U/mL (n = 918)	54 (0.50–0.57)	78 (0.72–0.83)	86 (0.82–0.89)	41 (0.36–0.45)	61 (0.57–0.64)
Pathologic assessment	44 (0.41–0.47)	99 (0.98–0.99)	98 (0.96–0.99)	58 (0.55–0.60)	68 (0.66–0.70)
BiliSeqV2/V3 testing	82 (0.79–0.84)	98 (0.97–0.99)	98 (0.97–0.99)	81 (0.78–0.83)	89 (0.87–0.90)
Pathologic assessment and BiliSeqV2/V3 testing	88 (0.86–0.90)	97 (0.96–0.98)	97 (0.96–0.98)	86 (0.84–0.89)	92 (0.90–0.93)

Patients with biliary brushings (n = 1815)					
Pathologic assessment	36 (0.33–0.39)	99 (0.98–0.99)	97 (0.95–0.99)	55 (0.53–0.58)	64 (0.62–0.66)
BiliSeqV2/V3 testing	81 (0.78–0.83)	99 (0.97–0.99)	99 (0.97–0.99)	80 (0.78–0.83)	89 (0.87–0.90)
Pathologic assessment and BiliSeqV2/V3 testing	86 (0.84–0.88)	97 (0.96–0.98)	98 (0.96–0.98)	85 (0.82–0.87)	91 (0.90–0.92)

Patients with biliary biopsies (n = 833)					
Pathologic assessment	47 (0.43–0.51)	100 (0.99–1.00)	100 (0.98–1.00)	55 (0.51–0.59)	68 (0.65–0.71)
BiliSeqV2/V3 testing	85 (0.81–0.88)	99 (0.97–1.00)	99 (0.98–1.00)	81 (0.77–0.85)	90 (0.88–0.92)
Pathologic assessment and BiliSeqV2/V3 testing	88 (0.85–0.91)	99 (0.97–1.00)	99 (0.98–1.00)	84 (0.80–0.88)	92 (0.90–0.94)

Patients with bile (n = 107)					
Pathologic assessment	17 (0.07–0.32)	100 (0.95–1.00)	100 (0.59–1.00)	66 (0.56–0.75)	68 (0.59–0.77)
BiliSeqV2/V3 testing	73 (0.57–0.86)	97 (0.89–1.00)	94 (0.79–0.99)	85 (0.75–0.92)	88 (0.80–0.93)
Pathologic assessment and BiliSeqV2/V3 testing	73 (0.57–0.86)	97 (0.89–1.00)	94 (0.79–0.99)	85 (0.75–0.92)	88 (0.80–0.93)

**Table 4.** Diagnostic Performance of BiliSeqV2/V3 Among Patients With PSC and Patients Undergoing Repeat Testing

Parameter	Sensitivity, % (95% CI)	Specificity, % (95% CI)	Positive predictive value, % (95% CI)	Negative predictive value, % (95% CI)	Accuracy, % (95% CI)
Indeterminate strictures (n = 548)					
CA19-9 ≥44 U/mL (n = 377)	61 (0.54–0.68)	53 (0.46–0.61)	60 (0.52–0.66)	55 (0.47–0.63)	58 (0.52–0.63)
CA19-9 >129 U/mL (n = 377)	44 (0.37–0.51)	79 (0.72–0.84)	70 (0.61–0.78)	56 (0.49–0.62)	60 (0.55–0.65)
BiliSeqV2/V3 testing	77 (0.71–0.82)	99 (0.97–1.00)	98 (0.95–0.99)	85 (0.80–0.88)	89 (0.86–0.92)
Pathologic assessment and BiliSeqV2/V3 testing	77 (0.71–0.82)	98 (0.96–0.99)	97 (0.94–0.99)	85 (0.80–0.88)	89 (0.86–0.92)

PSC patients (n = 327)					
CA19-9 ≥44 U/mL (n = 145)	66 (0.52–0.78)	63 (0.52–0.73)	51 (0.38–0.63)	76 (0.65–0.85)	64 (0.56–0.72)
CA19-9 >129 U/mL (n = 145)	45 (0.32–0.60)	88 (0.80–0.94)	69 (0.51–0.83)	74 (0.64–0.82)	72 (0.64–0.80)
Pathologic assessment	26 (0.17–0.36)	100 (0.98–1.00)	96 (0.79–1.00)	78 (0.73–0.83)	79 (0.74–0.84)
BiliSeqV2/V3 testing	85 (0.76–0.92)	98 (0.96–1.00)	95 (0.88–0.99)	95 (0.91–0.97)	95 (0.92–0.97)
Pathologic assessment and BiliSeqV2/V3 testing	89 (0.80–0.94)	98 (0.95–0.99)	94 (0.87–0.98)	96 (0.93–0.98)	95 (0.93–0.97)

Non-PSC patients (n = 1631)					
CA19-9 ≥44 U/mL (n = 772)	69 (0.66–0.73)	50 (0.42–0.57)	83 (0.79–0.86)	32 (0.26–0.38)	65 (0.62–0.68)
CA19-9 >129 U/mL (n = 772)	54 (0.50–0.58)	73 (0.66–0.79)	87 (0.84–0.91)	32 (0.27–0.36)	58 (0.55–0.62)
Pathologic assessment	46 (0.42–0.49)	99 (0.97–0.99)	98 (0.96–0.99)	53 (0.50–0.56)	66 (0.64–0.68)
BiliSeqV2/V3 testing	81 (0.78–0.83)	98 (0.97–0.99)	99 (0.97–0.99)	76 (0.73–0.79)	88 (0.86–0.89)
Pathologic assessment and BiliSeqV2/V3 testing	87 (0.85–0.89)	97 (0.95–0.98)	98 (0.96–0.99)	83 (0.80–0.85)	91 (0.89–0.92)

Patients with repeat testing (n = 260), initial testing					
Pathologic assessment	14 (0.08–0.21)	99 (0.96–1.00)	94 (0.70–1.00)	60 (0.54–0.67)	63 (0.56–0.68)
BiliSeqV2/V3 testing	72 (0.63–0.80)	97 (0.93–0.99)	95 (0.88–0.99)	82 (0.76–0.88)	87 (0.82–0.90)
Pathologic assessment and BiliSeqV2/V3 testing	75 (0.66–0.83)	97 (0.92–0.99)	94 (0.87–0.98)	84 (0.77–0.89)	87 (0.83–0.91)

Patients with repeat testing (n = 260), repeat testing					
Pathologic assessment	33 (0.25–0.43)	98 (0.94–1.00)	92 (0.80–0.98)	66 (0.60–0.73)	70 (0.64–0.76)
BiliSeqV2/V3 testing	85 (0.77–0.91)	93 (0.87–0.96)	90 (0.82–0.95)	89 (0.83–0.93)	89 (0.85–0.93)
Pathologic assessment and BiliSeqV2/V3 testing	93 (0.86–0.97)	92 (0.86–0.96)	90 (0.82–0.94)	94 (0.89–0.98)	92 (0.88–0.95)

## Data Availability

The data that support the findings of this study are available from the corresponding author on reasonable request.

## WHAT YOU NEED TO KNOW

### BACKGROUND AND CONTEXT

Next-generation sequencing of endoscopic retrograde cholangiopancreatography–obtained specimens has become a promising adjunct to the evaluation of bile duct strictures, but previous studies have been limited to retrospective analyses and single-institutional studies.

### NEW FINDINGS

A 6-year, prospective, multi-institutional study revealed next-generation sequencing using DNA/RNA-based panels (BiliSeqV2/V3) of endoscopic retrograde cholangiopancreatography–obtained biliary specimens were superior to current diagnostic modalities, especially among high-risk populations, with a sensitivity and specificity of 82% and 98%.

### LIMITATIONS

This study does not address the ideal DNA/RNA-based panel for next-generation sequencing of endoscopic retrograde cholangiopancreatography–obtained specimens, but BiliSeqV2/V3 includes most of the known genomic alterations associated with neoplasia arising or secondarily involving the bile duct.

### CLINICAL RESEARCH RELEVANCE

The application of BiliSeqV2/V3 testing of endoscopic retrograde cholangiopancreatography–obtained specimens improved the early diagnosis of neoplastic bile duct strictures and identified actionable molecular alterations that influenced treatment decisions and patient outcomes.

### BASIC RESEARCH RELEVANCE

Considering the diagnostic significance of various genomic alterations including their combination, the findings elucidated by BiliSeqV2/V3 testing may provide further insight into the molecular pathogenesis of pancreatobiliary cancers, especially in the context of primary sclerosing cholangitis.

## Abbreviations used in this paper:

BiliSeqV1: BiliSeq version 1
BiliSeqV2: BiliSeq version 2
BiliSeqV3: BiliSeq version 3
CA19-9: carbohydrate antigen 19-9
CCA: cholangiocarcinoma
ERCP: endoscopic retrograde cholangiopancreatography
EUS: endoscopic ultrasound
FISH: fluorescence in situ hybridization
FNA: fine-needle aspiration
LOD: limit of detection
MRCP: magnetic resonance cholangiopancreatography
NGS: next-generation sequencing
PSC: primary sclerosing cholangitis
RECIST: Response Evaluation Criteria in Solid Tumors
VAF: variable allele frequency
