# Supplementary material for: DNA/RNA-Based Next-Generation Sequencing Improves the Early Diagnosis and Management of Neoplastic Bile Duct Strictures: A 6-Year, Prospective, Multi-Institutional, Real-Time Study
Source: Gastroenterology. Author manuscript; Available in PMC 2026 Aug 8. (PMC13452169; doi:10.1053/j.gastro.2026.02.040)
Supplement: Supplementary file 1 [file NIHMS2198258-supplement-1.pdf]

Supplementary Contents

Supplementary Materials and Methods	Page 2
Supplementary Results	Page 4
Supplementary Figure Legend	Page 42
Supplementary Figure 1	Page 49
Supplementary Figure 2	Page 50
Supplementary Figure 3	Page 51
Supplementary Figure 4	Page 52
Supplementary Figure 5	Page 53
Supplementary Figure 6	Page 54
Supplementary Figure 7	Page 55
Supplementary Figure 8	Page 56
Supplementary Figure 9	Page 57
Supplementary Figure 10	Page 58
Supplementary Figure 11	Page 59
Supplementary Figure 12	Page 60
Supplementary Figure 13	Page 61
Supplementary Figure 14	Page 62
Supplementary Figure 15	Page 63
Supplementary Figure 16	Page 64
Supplementary Figure 17	Page 65
Supplementary Figure 18	Page 66
Supplementary Figure 19	Page 67
Supplementary Figure 20	Page 68
Supplementary Figure 21	Page 69
Supplementary Figure 22	Page 70
Supplementary Figure 23	Page 71

Supplementary Materials and Methods

Analytical Validation of BiliSeqV2 and BiliSeqV3 per CAP/CLIA Guidelines

BiliSeqV2 and BiliSeqV3 are two independent next-generation sequencing (NGS) panels that were developed and validated within the Clinical Laboratory Improvement Amendments (CLIA)-certified and College of American Pathologists (CAP)-accredited Molecular and Genomic Pathology (MGP) laboratory at the University of Pittsburgh Medical Center. Both panels were built upon the same amplification-based targeted sequencing architecture and Ion Torrent semiconductor sequencing technology as the original BiliSeqV1 assay. BiliSeqV2 retained the 28-gene DNA panel of BiliSeqV1 and incorporated RNA-based testing for 167 fusion genes. BiliSeqV3 expanded the DNA component to 161 cancer-associated genes and the RNA component to 763 fusion genes.

Analytical validation of BiliSeqV2 and BiliSeqV3 was performed in accordance with CAP and CLIA guidelines for NGS-based oncology assays. Validation studies were conducted using 80 archived bile duct specimens (40 neoplastic and 40 benign) with confirmed diagnostic pathology from surgical resection or clinicopathologic follow-up as previously described. The 80 validation specimens were derived from the original BiliSeqV1 cohort and included bile duct brushings and biopsies collected during endoscopic retrograde cholangiopancreatography (ERCP) for indeterminate bile duct strictures. Specimens were selected to represent the spectrum of genomic alterations and pathologic diagnoses encountered in clinical practice. Neoplastic specimens (n=40) included cholangiocarcinoma (intrahepatic, perihilar, and distal), pancreatic ductal adenocarcinoma, gallbladder adenocarcinoma, and ampullary adenocarcinoma. Benign specimens (n=40) included primary sclerosing cholangitis, IgG4-related cholangitis, and other benign cholangiopathies.

Accuracy was determined by comparing BiliSeqV2 and BiliSeqV3 results with the original BiliSeqV1 testing results and confirmed diagnostic pathology. For the 80 validation specimens, both BiliSeqV2 and BiliSeqV3 demonstrated 100% concordance with BiliSeqV1 for all previously

identified genomic alterations in overlapping genes. BiliSeqV3 detected additional clinically relevant alterations in genes not covered by BiliSeqV1 or BiliSeqV2, including *ARID1A*, *SF3B1*, *BAP1*, *PBRM1*, *RNF43*, *BRCA1*, *BRCA2*, and *PALB2*. Intra-run and inter-run precision were assessed by testing representative specimens (positive and negative for genomic alterations) in triplicate across independent sequencing runs. Reproducibility was 100% for variant detection across all tested specimens, with coefficient of variation (CV) for variant allele frequency (VAF) measurements of <5% for variants with VAF >10%.

The limit of detection (LOD) for single nucleotide variants (SNVs) and small insertions/deletions (indels) was established at 3% VAF for BiliSeqV2 (consistent with BiliSeqV1) and 1.5% VAF for BiliSeqV3. LOD was determined using serial dilutions of positive control specimens with known mutations. For copy number alterations, the LOD was established at ≥6 copies for amplifications. The minimum sequencing depth of coverage was 500x for BiliSeqV2 and 1000x for BiliSeqV3. Analytical specificity was assessed using normal control specimens and specimens with known variants at subthreshold levels. No false-positive calls were observed in negative control specimens, demonstrating 100% specificity for distinguishing true variants from sequencing artifacts. RNA fusion detection was validated using positive control cell lines with known fusions and clinical specimens with previously characterized fusions. A minimum of 50 fusion-specific reads crossing the fusion breakpoint were required for a positive fusion call. Validation demonstrated 100% sensitivity and specificity for fusion detection in specimens with adequate RNA quality.

Quality control measures implemented for BiliSeqV2 and BiliSeqV3 testing include positive controls with known mutations run with each sequencing batch, negative controls (no-template controls) to detect contamination, internal quality metrics including minimum sequencing depth, uniformity of coverage, and mapping quality scores, manual review of all positive calls by a board-certified molecular pathologist, and proficiency testing through CAP laboratory accreditation program surveys. BiliSeqV2 testing was performed on the Ion S5 XL System

(Thermo Fisher Scientific, Waltham, MA) using Ion AmpliSeq chemistry. BiliSeqV3 testing utilized the Ion GeneStudio S5 Prime System with enhanced throughput and coverage uniformity. Clinical testing was performed with a 7-to-10-day turnaround time from specimen receipt to final report generation. Each variant was prioritized according to the 2017 AMP/ASCO/CAP joint consensus guidelines for interpretation of sequence variants in cancer using a tier-based system, with Tier I and Tier II variants used for subsequent analysis.

Supplementary Results

BiliSeq Version 2 Analysis

The BiliSeq Version 2 (BiliSeqV2) cohort consisted of 217 patients who underwent endoscopic retrograde cholangiopancreatography (ERCP) for evaluation of a biliary duct stricture, yielding a total of 316 biliary specimens submitted for molecular testing (detailed information in Supplementary Table 4). Patients ranged in age from 20 to 96 years (mean, 65.9 years; median, 68.0 years). The cohort was predominantly male with 127 of 217 (58.5%) male patients and 90 of 217 (41.5%) female patients. Regarding race and ethnicity, the majority of patients were Caucasian (186 of 217, 85.7%), followed by Black (20 of 217, 9.2%), and Asian (7 of 217, 3.2%). No report of race/ethnicity was obtained for four patients (4 of 217, 1.8%). A documented history of primary sclerosing cholangitis (PSC) was noted for 51 of 217 (23.5%) patients, while 157 of 217 (72.4%) patients did not have PSC, and 9 of 217 (4.1%) had an unspecified PSC status. Indeterminate strictures, defined as strictures with inconclusive ERCP imaging and negative pathologic evaluation after ERCP bile duct sampling, were present in 74 of 217 (34.1%) patients.

Serum carbohydrate antigen 19-9 (CA19-9) levels were available for 149 of 217 (68.7%) patients at the time of ERCP and ranged from 0.8 to 20,000 U/mL (mean, 1,190.5 U/mL; median, 63.4 U/mL). The biliary duct strictures were located within the following anatomic sites: distal in 103 of 217 (47.5%) patients, intrahepatic/hilar in 92 of 217 (42.4%) patients, and middle bile duct in 22 of 217 (10.1%) patients. Among the 217 patients, specimens submitted for cytopathologic

and histopathologic evaluation included biliary brushings for 167 (77.0%) patients and biliary biopsies for 144 (66.4%) patients. Additionally, bile specimens were available for pathologic evaluation in 5 (2.3%) patients. Neoplastic pathology, defined as at least suspicious for adenocarcinoma, was identified in 40 of 167 (24.0%) brushing specimens and 40 of 144 (27.8%) biopsy specimens. None of the 5 (0%) bile specimens demonstrated neoplasia. A total of 316 biliary specimens from 217 patients were submitted for BiliSeqV2 testing, consisting of 167 brushings, 144 biopsies, and 5 bile specimens. Positive BiliSeqV2 results were detected in 71 of 167 (42.5%) brushing specimens, 66 of 144 (45.8%) biopsy specimens, and 2 of 5 (40.0%) bile specimens.

Follow-up was available for 213 of 217 (98.2%) patients. The duration of follow-up ranged from 4 to 60 months (mean, 25.8 months; median, 23.0 months). Follow-up consisted of diagnostic pathology for 98 of 213 (46.0%) patients and clinicoradiographic impression for 115 of 213 (54.0%) patients. Among the 115 patients with clinicoradiographic follow-up, the duration of surveillance ranged from 4 to 60 months (mean, 27.8 months; median, 24.0 months). Pathologic confirmation was achieved through various methods including Whipple resection (27 of 213, 12.7%), hepatectomy (19 of 213, 8.9%), concurrent ERCP biopsy (18 of 213, 8.5%), pancreatic EUS-FNA (10 of 213, 4.7%), bile duct EUS-FNA (9 of 213, 4.2%), bile duct resection (6 of 213, 2.8%), liver core needle biopsy (4 of 213, 1.9%), gallbladder and bile duct resection (3 of 213, 1.4%), hepatectomy and bile duct resection (1 of 213, 0.5%), and autopsy (1 of 213, 0.5%).

The final diagnoses included 12 distal cholangiocarcinomas, 12 perihilar cholangiocarcinomas, 5 intrahepatic cholangiocarcinomas, 3 pancreatic ductal adenocarcinomas, 3 gallbladder adenocarcinomas and 22 benign cholangiopathies. Among patients with a benign final diagnosis, including benign cholangiopathy and IgG4 disease (n=87), the duration of follow-up ranged from 12 to 60 months (mean, 34.0 months; median, 33.0 months). Among patients with a neoplastic final diagnosis (n=122), the duration of follow-up ranged from 4 to 59 months (mean, 19.3 months; median, 14.0 months).

Diagnostic performance was evaluated for BiliSeqV2, pathologic evaluation, and serum CA19-9 in detecting malignancy among patients with available follow-up (**Supplementary Figure 3**). BiliSeqV2 demonstrated a sensitivity of 73% (95% CI, 0.64-0.81) and specificity of 98% (95% CI, 0.92-1.00) for the detection of malignancy in 213 patients with follow-up, with a positive predictive value (PPV) of 98% (95% CI, 0.93-1.00), negative predictive value (NPV) of 71% (95% CI, 0.62-0.79), and accuracy of 83% (95% CI, 0.77-0.88). The area under the receiver operating characteristic curve (AUC) was 0.85 for BiliSeqV2. Pathologic evaluation alone demonstrated a sensitivity of 53% (95% CI, 0.44-0.62) and specificity of 100% (95% CI, 0.96-1.00), with a PPV of 100% (95% CI, 0.95-1.00), NPV of 60% (95% CI, 0.51-0.68), accuracy of 72% (95% CI, 0.66-0.78), and AUC of 0.77. Among the 149 patients with available serum CA19-9 levels, CA19-9 at a threshold of ≥44 U/mL demonstrated a sensitivity of 73% (95% CI, 0.63-0.81) and specificity of 47% (95% CI, 0.32-0.62), with a PPV of 75% (95% CI, 0.65-0.83), NPV of 44% (95% CI, 0.30-0.59), accuracy of 64% (95% CI, 0.56-0.72), and AUC of 0.60. CA19-9 at a threshold of >129 U/mL demonstrated a sensitivity of 52% (95% CI, 0.42-0.62) and specificity of 87% (95% CI, 0.74-0.95), with a PPV of 90% (95% CI, 0.79-0.96), NPV of 46% (95% CI, 0.35-0.56), accuracy of 63% (95% CI, 0.55-0.71), and AUC of 0.70. BiliSeqV2 demonstrated significantly higher sensitivity compared to pathologic evaluation alone (p=0.001) and CA19-9>129 U/mL (p=0.002). BiliSeqV2 demonstrated significantly higher specificity compared to CA19-9≥44 U/mL (p<0.001). BiliSeqV2 also demonstrated significantly higher accuracy compared to CA19-9≥44 U/mL (p=0.003) and pathologic evaluation alone (p=0.004).

The combination of pathologic evaluation and BiliSeqV2 demonstrated a sensitivity of 86% (95% CI, 0.78-0.91) and specificity of 98% (95% CI, 0.92-1.00), with a PPV of 98% (95% CI, 0.94-1.00), NPV of 83% (95% CI, 0.74-0.89), accuracy of 91% (95% CI, 0.86-0.94), and AUC of 0.92. The combination of pathologic evaluation and BiliSeqV2 demonstrated significantly higher sensitivity compared to BiliSeqV2 alone (p<0.001), pathologic evaluation alone (p<0.001), CA19-9≥44 U/mL (p=0.013), and CA19-9>129 U/mL (p<0.001). The combination of pathologic

evaluation and BiliSeqV2 also demonstrated significantly higher accuracy compared to BiliSeqV2 alone (p<0.001), pathologic evaluation alone (p<0.001), CA19-9≥44 U/mL (p<0.001), and CA19-9>129 U/mL (p<0.001).

BiliSeq Version 3 Analysis

The BiliSeq Version 3 (BiliSeqV3) cohort comprised 1,863 patients who underwent ERCP for evaluation of a biliary duct stricture, yielding a total of 2,549 biliary specimens submitted for molecular testing (detailed information in Supplementary Table 4). Patients ranged in age from 8 to 97 years (mean, 65.1 years; median, 67.0 years; standard deviation, 15.6 years). The cohort was predominantly male with 1,037 of 1,863 (55.7%) male patients and 826 of 1,863 (44.3%) female patients. Regarding race and ethnicity, the majority of patients were Caucasian (1,417 of 1,863, 76.1%), followed by Black (184 of 1,863, 9.9%), Hispanic (127 of 1,863, 6.8%), Asian (65 of 1,863, 3.5%), and not specified (70 of 1,863, 3.8%).

A documented history of PSC was noted for 290 of 1,863 (15.6%) patients, while 1,532 of 1,863 (82.2%) patients did not have PSC, and 41 of 1,863 (2.2%) had an unspecified PSC status. Indeterminate strictures, defined as strictures with inconclusive ERCP imaging and negative pathologic evaluation after ERCP bile duct sampling, were present in 484 of 1,863 (26.0%) patients. Germline alteration testing was performed for all 1,863 patients in the BiliSeqV3 cohort, with germline alterations identified in 69 of 1,863 (3.7%) patients, while 1,794 of 1,863 (96.3%) patients did not harbor germline alterations.

Serum CA19-9 levels were available for 777 of 1,863 (41.7%) patients at the time of ERCP and ranged from 0.5 to 273,872 U/mL (mean, 2,640.5 U/mL; median, 83.6 U/mL). The biliary duct strictures were located within the following anatomic sites: distal in 911 of 1,863 (48.9%) patients, intrahepatic/hilar in 687 of 1,863 (36.9%) patients, middle bile duct in 230 of 1,863 (12.3%) patients, and entire bile duct in 19 of 1,863 (1.0%) patients.

Among the 1,863 patients, specimens submitted for cytopathologic and histopathologic evaluation included biliary brushings for 1,733 (93.0%) patients and biliary biopsies for 697 (37.4%) patients. Additionally, bile specimens were available for pathologic evaluation in 119 (6.4%) patients. Neoplastic pathology, defined as at least suspicious for adenocarcinoma, was identified in 334 of 1,733 (19.3%) brushing specimens, 198 of 697 (28.4%) biopsy specimens, and 7 of 119 (5.9%) bile specimens.

A total of 2,549 biliary specimens from 1,863 patients were submitted for BiliSeqV3 molecular testing, consisting of 1,733 brushings, 697 biopsies, and 119 bile specimens. Positive BiliSeqV3 results were detected in 774 of 1,733 (44.7%) brushing specimens, 367 of 697 (52.7%) biopsy specimens, and 34 of 119 (28.6%) bile specimens. Follow-up was available for 1,766 of 1,863 (94.8%) patients. The duration of follow-up ranged from 1 to 59 months (mean, 16.6 months; median, 14.0 months). Follow-up consisted of diagnostic confirmation for 588 of 1,766 (33.3%) patients and clinicoradiographic impression for 1,178 of 1,766 (66.7%) patients. Among the 1,178 patients with clinicoradiographic follow-up, the duration of surveillance ranged from 1 to 59 months (mean, 17.7 months; median, 14.0 months). Pathologic confirmation was achieved through various methods including Whipple resection (210 of 1,766, 11.9%), concurrent ERCP biopsy (112 of 1,766, 6.3%), hepatectomy (81 of 1,766, 4.6%), bile duct resection (42 of 1,766, 2.4%), pancreatic EUS-FNA (35 of 1,766, 2.0%), hepatectomy and bile duct resection (32 of 1,766, 1.8%), liver core needle biopsy (27 of 1,766, 1.5%), bile duct EUS-FNA (21 of 1,766, 1.2%), autopsy (10 of 1,766, 0.6%), gallbladder and bile duct resection (8 of 1,766, 0.5%), liver transplantation (7 of 1,766, 0.4%), ampullary EUS-FNA (2 of 1,766, 0.1%), and liver EUS-FNA (1 of 1,766, 0.1%). Among patients with a benign final diagnosis, including benign cholangiopathy and IgG4 disease (n=784), the duration of follow-up ranged from 12 to 59 months (mean, 22.8 months; median, 20.0 months). Among patients with a neoplastic final diagnosis (n=971), the duration of follow-up ranged from 1 to 56 months (mean, 11.5 months; median, 9.0 months).

The final diagnoses among the 1,766 patients with available follow-up included benign cholangiopathy (753 of 1,766, 42.6%), pancreatic ductal adenocarcinoma (283 of 1,766, 16.0%), intrahepatic cholangiocarcinoma (253 of 1,766, 14.3%), extrahepatic cholangiocarcinoma (230 of 1,766, 13.0%), ampullary adenocarcinoma (67 of 1,766, 3.8%), perihilar cholangiocarcinoma (61 of 1,766, 3.5%), IgG4 disease (31 of 1,766, 1.8%), gallbladder adenocarcinoma (25 of 1,766, 1.4%), metastatic colonic adenocarcinoma (10 of 1,766, 0.6%), low-grade dysplasia (8 of 1,766, 0.5%), high-grade dysplasia (8 of 1,766, 0.5%), hepatocellular carcinoma (6 of 1,766, 0.3%), B-cell lymphoma (6 of 1,766, 0.3%), metastatic colorectal adenocarcinoma (5 of 1,766, 0.3%), metastatic lung adenocarcinoma (4 of 1,766, 0.2%), unknown primary (3 of 1,766, 0.2%), metastatic prostate cancer (2 of 1,766, 0.1%), metastatic gastric cancer (2 of 1,766, 0.1%), metastatic ileal neuroendocrine tumor (2 of 1,766, 0.1%), mixed hepatocellular carcinoma-cholangiocarcinoma (2 of 1,766, 0.1%), metastatic breast cancer (2 of 1,766, 0.1%), metastatic pancreatic cancer (1 of 1,766, 0.1%), metastatic endometrioid carcinoma (1 of 1,766, 0.1%), and metastatic appendiceal adenocarcinoma (1 of 1,766, 0.1%).

Diagnostic performance was evaluated for BiliSeqV3, pathologic evaluation, and serum CA19-9 in detecting malignancy among patients with available follow-up (**Supplementary Figure 4**). BiliSeqV3 demonstrated a sensitivity of 83% (95% CI, 0.80-0.85) and specificity of 98% (95% CI, 0.97-0.99) for the detection of malignancy in 1,766 patients with follow-up, with a positive predictive value (PPV) of 98% (95% CI, 0.97-0.99), negative predictive value (NPV) of 82% (95% CI, 0.79-0.84), and accuracy of 90% (95% CI, 0.88-0.91). The area under the receiver operating characteristic curve (AUC) was 0.90 for BiliSeqV3. Pathologic evaluation alone demonstrated a sensitivity of 42% (95% CI, 0.39-0.46) and specificity of 99% (95% CI, 0.98-0.99) in 1,761 patients, with a PPV of 98% (95% CI, 0.96-0.99), NPV of 58% (95% CI, 0.55-0.60), accuracy of 67% (95% CI, 0.65-0.70), and AUC of 0.71. Among the 769 patients with available serum CA19-9 levels, CA19-9 at a threshold of ≥44 U/mL demonstrated a sensitivity of 69% (95% CI, 0.64-0.72) and specificity of 56% (95% CI, 0.49-0.62), with a PPV of 80% (95% CI, 0.76-0.83), NPV of 41% (95%

CI, 0.36-0.47), accuracy of 65% (95% CI, 0.61-0.68), and AUC of 0.62. CA19-9 at a threshold of >129 U/mL demonstrated a sensitivity of 54% (95% CI, 0.50-0.58) and specificity of 76% (95% CI, 0.70-0.81), with a PPV of 85% (95% CI, 0.81-0.88), NPV of 40% (95% CI, 0.35-0.44), accuracy of 60% (95% CI, 0.57-0.64), and AUC of 0.65. BiliSeqV3 demonstrated significantly higher sensitivity compared to pathologic evaluation alone (p<0.001), CA19-9≥44 U/mL (p<0.001), and CA19-9>129 U/mL (p<0.001). BiliSeqV3 demonstrated significantly higher specificity compared to CA19-9≥44 U/mL (p<0.001) and CA19-9>129 U/mL (p<0.001). BiliSeqV3 also demonstrated significantly higher accuracy compared to pathologic evaluation alone (p<0.001), CA19-9≥44 U/mL (p<0.001), and CA19-9>129 U/mL (p<0.001).

The combination of pathologic evaluation and BiliSeqV3 demonstrated a sensitivity of 88% (95% CI, 0.86-0.90) and specificity of 97% (95% CI, 0.95-0.98), with a PPV of 97% (95% CI, 0.96-0.98), NPV of 86% (95% CI, 0.84-0.89), accuracy of 92% (95% CI, 0.91-0.93), and AUC of 0.92. The combination of pathologic evaluation and BiliSeqV3 demonstrated significantly higher sensitivity compared to BiliSeqV3 alone (p<0.001), pathologic evaluation alone (p<0.001), CA19-9≥44 U/mL (p<0.001), and CA19-9>129 U/mL (p<0.001). Specificity was significantly lower for the combination of pathologic evaluation and BiliSeqV3 compared to pathologic evaluation alone (p<0.001) and BiliSeqV3 alone (p=0.002). The combination of pathologic evaluation and BiliSeqV3 demonstrated significantly higher accuracy compared to BiliSeqV3 alone (p<0.001), pathologic evaluation alone (p<0.001), CA19-9≥44 U/mL (p<0.001), and CA19-9>129 U/mL (p<0.001).

Overall Cohort Analysis

Diagnostic performance was evaluated for BiliSeqV2/V3, pathologic evaluation, and serum CA19-9 in detecting malignancy among all patients with available follow-up (**Supplementary Figure 5**). BiliSeqV2/V3 demonstrated a sensitivity of 82% (95% CI, 0.79-0.84) and specificity of 98% (95% CI, 0.97-0.99) for the detection of malignancy in 1,979 patients with

follow-up, with a positive predictive value (PPV) of 98% (95% CI, 0.97-0.99), negative predictive value (NPV) of 81% (95% CI, 0.78-0.83), and accuracy of 89% (95% CI, 0.87-0.90). The area under the receiver operating characteristic curve (AUC) was 0.90 for BiliSeqV2/V3. Pathologic evaluation alone demonstrated a sensitivity of 44% (95% CI, 0.41-0.47) and specificity of 99% (95% CI, 0.98-0.99) in 1,974 patients, with a PPV of 98% (95% CI, 0.96-0.99), NPV of 58% (95% CI, 0.55-0.60), accuracy of 68% (95% CI, 0.66-0.70), and AUC of 0.71. Among the 918 patients with available serum CA19-9 levels, CA19-9 at a threshold of ≥44 U/mL demonstrated a sensitivity of 69% (95% CI, 0.65-0.73) and specificity of 54% (95% CI, 0.48-0.60), with a PPV of 79% (95% CI, 0.75-0.82), NPV of 42% (95% CI, 0.36-0.47), accuracy of 65% (95% CI, 0.62-0.68), and AUC of 0.62. CA19-9 at a threshold of >129 U/mL demonstrated a sensitivity of 54% (95% CI, 0.50-0.57) and specificity of 78% (95% CI, 0.72-0.83), with a PPV of 86% (95% CI, 0.82-0.89), NPV of 41% (95% CI, 0.36-0.45), accuracy of 61% (95% CI, 0.57-0.64), and AUC of 0.66. BiliSeqV2/V3 had significantly higher sensitivity compared to pathologic evaluation alone ($p<0.001$), CA19-9≥44 U/mL ($p<0.001$), and CA19-9>129 U/mL ($p<0.001$). BiliSeqV2/V3 demonstrated significantly higher specificity compared to CA19-9≥44 U/mL ($p<0.001$) and CA19-9>129 U/mL ($p<0.001$). BiliSeqV2/V3 also demonstrated significantly higher accuracy compared to pathologic evaluation alone ($p<0.001$), CA19-9≥44 U/mL ($p<0.001$), and CA19-9>129 U/mL ($p<0.001$).

The combination of pathologic evaluation and BiliSeqV2/V3 demonstrated a sensitivity of 88% (95% CI, 0.86-0.90) and specificity of 97% (95% CI, 0.96-0.98), with a PPV of 97% (95% CI, 0.96-0.98), NPV of 86% (95% CI, 0.84-0.88), accuracy of 92% (95% CI, 0.90-0.93), and AUC of 0.92. The combination of pathologic evaluation and BiliSeqV2/V3 demonstrated significantly higher sensitivity compared to BiliSeqV2/V3 alone ($p<0.001$), pathologic evaluation alone ($p<0.001$), CA19-9≥44 U/mL ($p<0.001$), and CA19-9>129 U/mL ($p<0.001$). Specificity was significantly lower for the combination of pathologic evaluation and BiliSeqV2/V3 compared to pathologic evaluation alone ($p<0.001$) and BiliSeqV2/V3 alone ($p=0.002$). The combination of pathologic evaluation and BiliSeqV2/V3 demonstrated significantly higher accuracy compared to

BiliSeqV2/V3 alone (p<0.001), pathologic evaluation alone (p<0.001), CA19-9≥44 U/mL (p<0.001), and CA19-9>129 U/mL (p<0.001).

Subanalysis of Bile Duct Brushings

Among the 1,815 patients with bile duct brushing specimens and available follow-up, BiliSeqV2/V3 demonstrated a sensitivity of 81% (95% CI, 0.78-0.83) and specificity of 99% (95% CI, 0.97-0.99) for the detection of malignancy, with a PPV of 99% (95% CI, 0.97-0.99), NPV of 80% (95% CI, 0.78-0.83), and accuracy of 89% (95% CI, 0.87-0.90) (**Supplementary Figure 6**). The AUC was 0.90 for BiliSeqV2/V3. Brushing pathology alone demonstrated a sensitivity of 36% (95% CI, 0.33-0.39) and specificity of 99% (95% CI, 0.98-0.99), with a PPV of 97% (95% CI, 0.95-0.99), NPV of 55% (95% CI, 0.53-0.58), accuracy of 64% (95% CI, 0.62-0.66), and AUC of 0.67. Among the 815 patients with available serum CA19-9 levels and brushing specimens, CA19-9 at a threshold of ≥44 U/mL demonstrated a sensitivity of 71% (95% CI, 0.67-0.75) and specificity of 54% (95% CI, 0.47-0.60), with a PPV of 78% (95% CI, 0.75-0.82), NPV of 44% (95% CI, 0.38-0.50), accuracy of 66% (95% CI, 0.62-0.69), and AUC of 0.62. CA19-9 at a threshold of >129 U/mL demonstrated a sensitivity of 55% (95% CI, 0.51-0.59) and specificity of 77% (95% CI, 0.71-0.82), with a PPV of 85% (95% CI, 0.81-0.88), NPV of 42% (95% CI, 0.37-0.47), accuracy of 62% (95% CI, 0.58-0.65), and AUC of 0.66.

BiliSeqV2/V3 had significantly higher sensitivity compared to brushing pathology alone (p<0.001), CA19-9≥44 U/mL (p<0.001), and CA19-9>129 U/mL (p<0.001). BiliSeqV2/V3 demonstrated significantly higher specificity compared to CA19-9≥44 U/mL (p<0.001) and CA19-9>129 U/mL (p<0.001). BiliSeqV2/V3 also demonstrated significantly higher accuracy compared to brushing pathology alone (p<0.001), CA19-9≥44 U/mL (p<0.001), and CA19-9>129 U/mL (p<0.001).

The combination of brushing pathology and BiliSeqV2/V3 demonstrated a sensitivity of 86% (95% CI, 0.84-0.88) and specificity of 97% (95% CI, 0.96-0.98), with a PPV of 98% (95% CI,

0.96-0.98), NPV of 85% (95% CI, 0.82-0.87), accuracy of 91% (95% CI, 0.90-0.92), and AUC of 0.92. The combination of brushing pathology and BiliSeqV2/V3 demonstrated significantly higher sensitivity compared to BiliSeqV2/V3 alone (p<0.001), brushing pathology alone (p<0.001), CA19-9≥44 U/mL (p<0.001), and CA19-9>129 U/mL (p<0.001). Specificity was significantly lower for the combination of brushing pathology and BiliSeqV2/V3 compared to brushing pathology alone (p<0.001) and BiliSeqV2/V3 alone (p=0.002). The combination of brushing pathology and BiliSeqV2/V3 demonstrated significantly higher accuracy compared to BiliSeqV2/V3 alone (p<0.001), brushing pathology alone (p<0.001), CA19-9≥44 U/mL (p<0.001), and CA19-9>129 U/mL (p<0.001).

Subanalysis of Bile Duct Biopsies

Among the 833 patients with bile duct biopsy specimens and available follow-up, BiliSeqV2/V3 had a sensitivity of 85% (95% CI, 0.81-0.88) and specificity of 99% (95% CI, 0.97-1.00) for the detection of malignancy, with a PPV of 99% (95% CI, 0.98-1.00), NPV of 81% (95% CI, 0.77-0.85), and accuracy of 90% (95% CI, 0.88-0.92) (**Supplementary Figure 7**). The AUC was 0.92 for BiliSeqV2/V3. Biopsy pathology alone demonstrated a sensitivity of 47% (95% CI, 0.43-0.51) and specificity of 100% (95% CI, 0.99-1.00), with a PPV of 100% (95% CI, 0.98-1.00), NPV of 55% (95% CI, 0.51-0.59), accuracy of 68% (95% CI, 0.65-0.71), and AUC of 0.74. Among the 531 patients with available serum CA19-9 levels and biopsy specimens, CA19-9 at a threshold of ≥44 U/mL demonstrated a sensitivity of 69% (95% CI, 0.64-0.73) and specificity of 56% (95% CI, 0.48-0.64), with a PPV of 80% (95% CI, 0.76-0.85), NPV of 40% (95% CI, 0.34-0.47), accuracy of 65% (95% CI, 0.61-0.69), and AUC of 0.62. CA19-9 at a threshold of >129 U/mL demonstrated a sensitivity of 53% (95% CI, 0.48-0.58) and specificity of 83% (95% CI, 0.76-0.89), with a PPV of 89% (95% CI, 0.84-0.93), NPV of 40% (95% CI, 0.34-0.46), accuracy of 61% (95% CI, 0.57-0.65), and AUC of 0.68.

A higher sensitivity for BiliSeqV2/V3 was observed as compared to biopsy pathology alone ($p<0.001$), CA19-9≥44 U/mL ($p<0.001$), and CA19-9>129 U/mL ($p<0.001$). BiliSeqV2/V3 demonstrated significantly higher specificity compared to CA19-9≥44 U/mL ($p<0.001$) and CA19-9>129 U/mL ($p<0.001$). BiliSeqV2/V3 also demonstrated significantly higher accuracy compared to biopsy pathology alone ($p<0.001$), CA19-9≥44 U/mL ($p<0.001$), and CA19-9>129 U/mL ($p<0.001$).

The combination of biopsy pathology and BiliSeqV2/V3 demonstrated a sensitivity of 88% (95% CI, 0.85-0.91) and specificity of 99% (95% CI, 0.97-1.00), with a PPV of 99% (95% CI, 0.98-1.00), NPV of 84% (95% CI, 0.80-0.88), accuracy of 92% (95% CI, 0.90-0.94), and AUC of 0.94. The combination of biopsy pathology and BiliSeqV2/V3 demonstrated significantly higher sensitivity compared to BiliSeqV2/V3 alone ($p=0.001$), biopsy pathology alone ($p<0.001$), CA19-9≥44 U/mL ($p<0.001$), and CA19-9>129 U/mL ($p<0.001$). The combination of biopsy pathology and BiliSeqV2/V3 demonstrated significantly higher accuracy compared to BiliSeqV2/V3 alone ($p<0.001$), biopsy pathology alone ($p<0.001$), CA19-9≥44 U/mL ($p<0.001$), and CA19-9>129 U/mL ($p<0.001$).

Subanalysis of Bile Collection Specimens

Among the 107 patients with bile specimens and available follow-up, BiliSeqV2/V3 had a sensitivity of 73% (95% CI, 0.57-0.86) and specificity of 97% (95% CI, 0.89-1.00) for the detection of malignancy, with a PPV of 94% (95% CI, 0.79-0.99), NPV of 85% (95% CI, 0.75-0.92), and accuracy of 88% (95% CI, 0.80-0.93). The AUC was 0.85 for BiliSeqV2/V3. Bile pathology alone demonstrated a sensitivity of 17% (95% CI, 0.07-0.32) and specificity of 100% (95% CI, 0.95-1.00), with a PPV of 100% (95% CI, 0.59-1.00), NPV of 66% (95% CI, 0.56-0.75), accuracy of 68% (95% CI, 0.59-0.77), and AUC of 0.59. Serum CA19-9 levels were available for only 4 patients with bile specimens, precluding meaningful statistical comparisons. BiliSeqV2/V3 demonstrated significantly higher sensitivity compared to bile pathology alone ($p<0.001$).

BiliSeqV2/V3 also demonstrated significantly higher accuracy compared to bile pathology alone ($p<0.001$).

The combination of bile pathology and BiliSeqV2/V3 demonstrated a sensitivity of 73% (95% CI, 0.57-0.86) and specificity of 97% (95% CI, 0.89-1.00), with a PPV of 94% (95% CI, 0.79-0.99), NPV of 85% (95% CI, 0.75-0.92), accuracy of 88% (95% CI, 0.80-0.93), and AUC of 0.85. No significant difference in sensitivity, specificity, or accuracy was observed between BiliSeqV2/V3 alone and the combination of bile pathology and BiliSeqV2/V3 ($p=1.0$ for all comparisons), reflecting the limited additional diagnostic yield of bile pathology in this specimen type.

Subanalysis of Intrahepatic/Hilar Bile Duct Strictures

Among the 742 patients with intrahepatic/hilar strictures and available follow-up, BiliSeqV2/V3 demonstrated a sensitivity of 80% (95% CI, 0.76-0.84) and specificity of 98% (95% CI, 0.95-0.99) for the detection of neoplasia, with a PPV of 98% (95% CI, 0.95-0.99), NPV of 79% (95% CI, 0.75-0.83), and accuracy of 88% (95% CI, 0.85-0.90) (**Supplementary Figure 8**). The AUC was 0.89 for BiliSeqV2/V3. Pathologic evaluation alone demonstrated a sensitivity of 36% (95% CI, 0.31-0.41) and specificity of 98% (95% CI, 0.96-0.99), with a PPV of 97% (95% CI, 0.93-0.99), NPV of 54% (95% CI, 0.50-0.58), accuracy of 63% (95% CI, 0.60-0.67), and AUC of 0.67. Among the 350 patients with available serum CA19-9 levels and intrahepatic/hilar strictures, CA19-9 at a threshold of ≥44 U/mL demonstrated a sensitivity of 71% (95% CI, 0.64-0.76) and specificity of 55% (95% CI, 0.45-0.64), with a PPV of 76% (95% CI, 0.70-0.82), NPV of 48% (95% CI, 0.39-0.57), accuracy of 65% (95% CI, 0.60-0.70), and AUC of 0.63. CA19-9 at a threshold of >129 U/mL demonstrated a sensitivity of 57% (95% CI, 0.50-0.63) and specificity of 77% (95% CI, 0.69-0.85), with a PPV of 84% (95% CI, 0.77-0.89), NPV of 47% (95% CI, 0.40-0.54), accuracy of 64% (95% CI, 0.58-0.69), and AUC of 0.67.

BiliSeqV2/V3 demonstrated significantly higher sensitivity compared to pathologic evaluation alone ($p<0.001$), CA19-9≥44 U/mL ($p=0.006$), and CA19-9>129 U/mL ($p<0.001$). BiliSeqV2/V3 demonstrated significantly higher specificity compared to CA19-9≥44 U/mL ($p<0.001$) and CA19-9>129 U/mL ($p<0.001$). BiliSeqV2/V3 also demonstrated significantly higher accuracy compared to pathologic evaluation alone ($p<0.001$), CA19-9≥44 U/mL ($p<0.001$), and CA19-9>129 U/mL ($p<0.001$).

The combination of pathologic evaluation and BiliSeqV2/V3 demonstrated a sensitivity of 86% (95% CI, 0.83-0.89) and specificity of 96% (95% CI, 0.93-0.98), with a PPV of 97% (95% CI, 0.94-0.98), NPV of 85% (95% CI, 0.80-0.88), accuracy of 91% (95% CI, 0.88-0.93), and AUC of 0.91. The combination demonstrated significantly higher sensitivity compared to BiliSeqV2/V3 alone ($p<0.001$), pathologic evaluation alone ($p<0.001$), CA19-9≥44 U/mL ($p<0.001$), and CA19-9>129 U/mL ($p<0.001$). The combination also demonstrated significantly higher accuracy compared to BiliSeqV2/V3 alone ($p<0.001$).

Subanalysis of Middle Bile Duct Strictures

Among the 235 patients with middle bile duct strictures and available follow-up, BiliSeqV2/V3 demonstrated a sensitivity of 86% (95% CI, 0.79-0.91) and specificity of 100% (95% CI, 0.97-1.00) for the detection of neoplasia, with a PPV of 100% (95% CI, 0.97-1.00), NPV of 86% (95% CI, 0.78-0.91), and accuracy of 92% (95% CI, 0.88-0.95) (**Supplementary Figure 9**). The AUC was 0.93 for BiliSeqV2/V3. Pathologic evaluation alone demonstrated a sensitivity of 51% (95% CI, 0.42-0.60) and specificity of 98% (95% CI, 0.93-1.00), with a PPV of 97% (95% CI, 0.90-1.00), NPV of 63% (95% CI, 0.55-0.70), accuracy of 73% (95% CI, 0.67-0.78), and AUC of 0.75. Among the 88 patients with available serum CA19-9 levels and middle bile duct strictures, CA19-9 at a threshold of ≥44 U/mL demonstrated a sensitivity of 68% (95% CI, 0.54-0.79) and specificity of 55% (95% CI, 0.36-0.74), with a PPV of 75% (95% CI, 0.62-0.86), NPV of 46% (95% CI, 0.29-0.63), accuracy of 64% (95% CI, 0.53-0.74), and AUC of 0.61. CA19-9 at a threshold of

>129 U/mL demonstrated a sensitivity of 56% (95% CI, 0.42-0.69) and specificity of 72% (95% CI, 0.53-0.87), with a PPV of 80% (95% CI, 0.65-0.91), NPV of 45% (95% CI, 0.30-0.60), accuracy of 61% (95% CI, 0.50-0.72), and AUC of 0.64. BiliSeqV2/V3 demonstrated significantly higher sensitivity compared to pathologic evaluation alone (p<0.001), CA19-9≥44 U/mL (p=0.02), and CA19-9>129 U/mL (p<0.001). BiliSeqV2/V3 demonstrated significantly higher specificity compared to CA19-9≥44 U/mL (p<0.001) and CA19-9>129 U/mL (p=0.008). BiliSeqV2/V3 also demonstrated significantly higher accuracy compared to pathologic evaluation alone (p<0.001), CA19-9≥44 U/mL (p<0.001), and CA19-9>129 U/mL (p<0.001).

The combination of pathologic evaluation and BiliSeqV2/V3 demonstrated a sensitivity of 93% (95% CI, 0.87-0.97) and specificity of 98% (95% CI, 0.93-1.00), with a PPV of 98% (95% CI, 0.94-1.00), NPV of 92% (95% CI, 0.86-0.96), accuracy of 95% (95% CI, 0.92-0.98), and AUC of 0.96. The combination demonstrated significantly higher sensitivity compared to BiliSeqV2/V3 alone (p=0.004), pathologic evaluation alone (p<0.001), CA19-9≥44 U/mL (p<0.001), and CA19-9>129 U/mL (p<0.001).

Subanalysis of Distal Bile Duct Strictures

Among the 982 patients with distal bile duct strictures and available follow-up, BiliSeqV2/V3 demonstrated a sensitivity of 82% (95% CI, 0.78-0.85) and specificity of 98% (95% CI, 0.96-0.99) for the detection of neoplasia, with a PPV of 98% (95% CI, 0.97-0.99), NPV of 80% (95% CI, 0.77-0.84), and accuracy of 89% (95% CI, 0.87-0.91). The AUC was 0.90 for BiliSeqV2/V3 (**Supplementary Figure 10**). Pathologic evaluation alone demonstrated a sensitivity of 48% (95% CI, 0.44-0.52) and specificity of 99% (95% CI, 0.98-1.00), with a PPV of 99% (95% CI, 0.97-1.00), NPV of 59% (95% CI, 0.55-0.63), accuracy of 70% (95% CI, 0.67-0.73), and AUC of 0.74. Among the 476 patients with available serum CA19-9 levels and distal strictures, CA19-9 at a threshold of ≥44 U/mL demonstrated a sensitivity of 68% (95% CI, 0.63-0.73) and specificity of 54% (95% CI, 0.44-0.63), with a PPV of 82% (95% CI, 0.77-0.86), NPV of 36% (95%

CI, 0.29-0.44), accuracy of 65% (95% CI, 0.60-0.69), and AUC of 0.61. CA19-9 at a threshold of >129 U/mL demonstrated a sensitivity of 51% (95% CI, 0.45-0.56) and specificity of 80% (95% CI, 0.71-0.87), with a PPV of 88% (95% CI, 0.83-0.92), NPV of 35% (95% CI, 0.29-0.41), accuracy of 58% (95% CI, 0.53-0.62), and AUC of 0.65.

BiliSeqV2/V3 demonstrated significantly higher sensitivity compared to pathologic evaluation alone (p<0.001), CA19-9≥44 U/mL (p<0.001), and CA19-9>129 U/mL (p<0.001). BiliSeqV2/V3 demonstrated significantly higher specificity compared to CA19-9≥44 U/mL (p<0.001) and CA19-9>129 U/mL (p<0.001). BiliSeqV2/V3 also demonstrated significantly higher accuracy compared to pathologic evaluation alone (p<0.001), CA19-9≥44 U/mL (p<0.001), and CA19-9>129 U/mL (p<0.001).

The combination of pathologic evaluation and BiliSeqV2/V3 had a sensitivity of 87% (95% CI, 0.84-0.90) and specificity of 97% (95% CI, 0.95-0.99), with a PPV of 98% (95% CI, 0.96-0.99), NPV of 85% (95% CI, 0.82-0.88), accuracy of 92% (95% CI, 0.90-0.93), and AUC of 0.92. The combination demonstrated significantly higher sensitivity compared to BiliSeqV2/V3 alone (p<0.001), pathologic evaluation alone (p<0.001), CA19-9≥44 U/mL (p<0.001), and CA19-9>129 U/mL (p<0.001). The combination also demonstrated significantly higher accuracy compared to BiliSeqV2/V3 alone (p<0.001).

Indeterminate Bile Duct Stricture Analysis

Among the 2,080 patients evaluated by BiliSeqV2/V3 testing, 558 (26.8%) patients presented with indeterminate strictures, defined as strictures with inconclusive ERCP imaging and negative pathologic evaluation after ERCP bile duct sampling (detailed information in Supplementary Table 4). This subgroup included 74 patients from the BiliSeqV2 cohort and 484 patients from the BiliSeqV3 cohort. Patients with indeterminate strictures ranged in age from 15 to 96 years (mean, 64.7 years; median, 66.0 years). The subgroup was predominantly male with 325 of 558 (58.2%) male patients and 233 of 558 (41.8%) female patients. Regarding race and

ethnicity, the majority of patients were Caucasian (478 of 558, 85.7%), followed by Black (48 of 558, 8.6%), Asian (13 of 558, 2.3%), and Hispanic (12 of 558, 2.2%).

A documented history of primary sclerosing cholangitis (PSC) was noted for 112 of 558 (20.1%) patients with indeterminate strictures, while 446 of 558 (79.9%) patients did not have PSC. Serum CA19-9 levels were available for 381 of 558 (68.3%) patients and ranged from 0.8 to 273,872 U/mL (mean, 1,981.3 U/mL; median, 50.0 U/mL). The indeterminate strictures were located within the following anatomic sites: distal in 296 of 558 (53.0%) patients, intrahepatic/hilar in 203 of 558 (36.4%) patients, middle bile duct in 56 of 558 (10.0%) patients, and entire bile duct in 3 of 558 (0.5%) patients.

A total of 830 biliary specimens from 558 patients with indeterminate strictures were submitted for BiliSeqV2/V3 molecular testing, consisting of 535 brushings, 292 biopsies, and 3 bile specimens. Positive BiliSeqV2/V3 results, indicating the presence of a genomic alteration, were detected in 187 of 535 (35.0%) brushing specimens, 89 of 294 (30.3%) biopsy specimens, and 1 of 4 (25.0%) bile specimens. Follow-up was available for 548 of 558 (98.2%) patients with indeterminate strictures. The duration of follow-up ranged from 1 to 60 months (mean, 19.9 months; median, 17.0 months). Among patients with a benign final diagnosis, including benign cholangiopathy and IgG4 disease (n=306), the duration of follow-up ranged from 12 to 60 months (mean, 25.2 months; median, 22.5 months). Among patients with a neoplastic final diagnosis (n=242), the duration of follow-up ranged from 1 to 57 months (mean, 13.2 months; median, 10.0 months). The final diagnoses among patients with indeterminate strictures and available follow-up included benign cholangiopathy (284 of 548, 51.8%), intrahepatic cholangiocarcinoma (72 of 548, 13.1%), pancreatic ductal adenocarcinoma (70 of 548, 12.8%), extrahepatic cholangiocarcinoma (56 of 548, 10.2%), IgG4 disease (22 of 548, 4.0%), gallbladder adenocarcinoma (10 of 548, 1.8%), ampullary adenocarcinoma (10 of 548, 1.8%), perihilar cholangiocarcinoma (6 of 548, 1.1%), B-cell lymphoma (5 of 548, 0.9%), low-grade dysplasia (3 of 548, 0.5%), metastatic colonic adenocarcinoma (2 of 548, 0.4%), metastatic lung

adenocarcinoma (2 of 548, 0.4%), metastatic renal cell carcinoma (1 of 548, 0.2%), high-grade dysplasia (1 of 548, 0.2%), metastatic pancreatic cancer (1 of 548, 0.2%), metastatic prostate cancer (1 of 548, 0.2%), metastatic endometrioid carcinoma (1 of 548, 0.2%), and metastatic breast cancer (1 of 548, 0.2%). Overall, 306 of 548 (55.8%) patients with available follow-up had a benign final diagnosis, while 242 of 548 (44.2%) patients had a neoplastic final diagnosis.

Diagnostic performance was evaluated for BiliSeqV2/V3 and serum CA19-9 in detecting neoplasia among patients with indeterminate strictures and available follow-up (**Supplementary Figure 11**). BiliSeqV2/V3 demonstrated a sensitivity of 77% (95% CI, 0.71-0.82) and specificity of 99% (95% CI, 0.97-1.00) for the detection of neoplasia in 548 patients with follow-up, with a positive predictive value (PPV) of 98% (95% CI, 0.95-0.99), negative predictive value (NPV) of 85% (95% CI, 0.80-0.88), and accuracy of 89% (95% CI, 0.86-0.92). The area under the receiver operating characteristic curve (AUC) was 0.88 for BiliSeqV2/V3. Among the 377 patients with available serum CA19-9 levels, CA19-9 at a threshold of ≥44 U/mL demonstrated a sensitivity of 61% (95% CI, 0.54-0.68) and specificity of 53% (95% CI, 0.46-0.61), with a PPV of 60% (95% CI, 0.52-0.66), NPV of 55% (95% CI, 0.47-0.63), accuracy of 58% (95% CI, 0.52-0.63), and AUC of 0.57. CA19-9 at a threshold of >129 U/mL demonstrated a sensitivity of 44% (95% CI, 0.37-0.51) and specificity of 79% (95% CI, 0.72-0.84), with a PPV of 70% (95% CI, 0.61-0.78), NPV of 56% (95% CI, 0.49-0.62), accuracy of 60% (95% CI, 0.55-0.65), and AUC of 0.61. BiliSeqV2/V3 demonstrated significantly higher sensitivity compared to CA19-9≥44 U/mL ($p=0.001$) and CA19-9>129 U/mL ($p<0.001$). BiliSeqV2/V3 demonstrated significantly higher specificity compared to CA19-9≥44 U/mL ($p<0.001$) and CA19-9>129 U/mL ($p<0.001$). BiliSeqV2/V3 also demonstrated significantly higher accuracy compared to CA19-9≥44 U/mL ($p<0.001$) and CA19-9>129 U/mL ($p<0.001$).

The combination of CA19-9≥44 U/mL and BiliSeqV2/V3 had a sensitivity of 91% (95% CI, 0.87-0.94) and specificity of 52% (95% CI, 0.44-0.59), with a PPV of 71% (95% CI, 0.66-0.76), NPV of 82% (95% CI, 0.73-0.88), accuracy of 74% (95% CI, 0.69-0.78), and AUC of 0.71 in 414

patients with both tests available. The combination of CA19-9>129 U/mL and BiliSeqV2/V3 demonstrated a sensitivity of 87% (95% CI, 0.82-0.91) and specificity of 77% (95% CI, 0.70-0.83), with a PPV of 83% (95% CI, 0.78-0.87), NPV of 82% (95% CI, 0.75-0.87), accuracy of 82% (95% CI, 0.78-0.86), and AUC of 0.82. The combination of CA19-9≥44 U/mL and BiliSeqV2/V3 demonstrated significantly higher sensitivity compared to BiliSeqV2/V3 alone ($p<0.001$), but significantly lower specificity ($p<0.001$) and accuracy ($p<0.001$). The combination of CA19-9>129 U/mL and BiliSeqV2/V3 demonstrated significantly higher sensitivity compared to BiliSeqV2/V3 alone ($p<0.001$), but significantly lower specificity ($p<0.001$) and accuracy ($p=0.004$).

Primary Sclerosing Cholangitis (PSC) Analysis

Among the 2,080 patients evaluated by BiliSeqV2/V3 testing, 341 (16.4%) patients had a documented history of PSC. This subgroup included 51 patients from the BiliSeqV2 cohort and 290 patients from the BiliSeqV3 cohort. Patients with PSC ranged in age from 12 to 87 years (mean, 50.6 years; median, 52.0 years), notably younger than the overall cohort. The subgroup was predominantly male with 214 of 341 (62.8%) male patients and 127 of 341 (37.2%) female patients. Regarding race and ethnicity, the majority of patients were Caucasian (238 of 341, 69.8%), followed by Black (49 of 341, 14.4%), Hispanic (18 of 341, 5.3%), and Asian (17 of 341, 5.0%). Indeterminate strictures were present in 112 of 341 (32.8%) patients with PSC. Serum CA19-9 levels were available for 146 of 341 (42.8%) patients and ranged from 0.8 to 21,000 U/mL (mean, 684.1 U/mL; median, 42.0 U/mL). The biliary duct strictures in patients with PSC were located predominantly in the intrahepatic/hilar region (243 of 341, 71.3%), followed by distal (51 of 341, 15.0%), middle bile duct (40 of 341, 11.7%), and entire bile duct (7 of 341, 2.1%).

A total of 458 biliary specimens from 341 patients with PSC were submitted for BiliSeqV2/V3 molecular testing, consisting of 318 brushings, 114 biopsies, and 26 bile specimens. Positive BiliSeqV2/V3 results, indicating the presence of a genomic alteration, were detected in 73 of 318 (23.0%) brushing specimens, 31 of 114 (27.2%) biopsy specimens, and 3 of 26 (11.5%)

bile specimens. Follow-up was available for 327 of 341 (95.9%) patients with PSC. The duration of follow-up ranged from 1 to 59 months (mean, 22.5 months; median, 21.0 months). Among patients with a benign final diagnosis, including benign cholangiopathy and IgG4 disease (n=238), the duration of follow-up ranged from 12 to 59 months (mean, 24.6 months; median, 22.0 months). Among patients with a neoplastic final diagnosis (n=84), the duration of follow-up ranged from 1 to 45 months (mean, 16.5 months; median, 14.0 months). The final diagnoses among patients with PSC and available follow-up included benign cholangiopathy (237 of 327, 72.5%), intrahepatic cholangiocarcinoma (55 of 327, 16.8%), extrahepatic cholangiocarcinoma (14 of 327, 4.3%), perihilar cholangiocarcinoma (9 of 327, 2.8%), low-grade dysplasia (4 of 327, 1.2%), pancreatic ductal adenocarcinoma (2 of 327, 0.6%), high-grade dysplasia (2 of 327, 0.6%), unknown primary (1 of 327, 0.3%), hepatocellular carcinoma (1 of 327, 0.3%), IgG4 disease (1 of 327, 0.3%), and gallbladder adenocarcinoma (1 of 327, 0.3%).

Diagnostic performance was evaluated for BiliSeqV2/V3, pathologic evaluation, and serum CA19-9 in detecting neoplasia among patients with PSC and available follow-up (**Supplementary Figure 12**). BiliSeqV2/V3 demonstrated a sensitivity of 85% (95% CI, 0.76-0.92) and specificity of 98% (95% CI, 0.96-1.00) for the detection of neoplasia in 327 patients with follow-up, with a positive predictive value (PPV) of 95% (95% CI, 0.88-0.99), negative predictive value (NPV) of 95% (95% CI, 0.91-0.97), and accuracy of 95% (95% CI, 0.92-0.97). The area under the receiver operating characteristic curve (AUC) was 0.92 for BiliSeqV2/V3. Pathologic evaluation alone demonstrated a sensitivity of 26% (95% CI, 0.17-0.36) and specificity of 100% (95% CI, 0.98-1.00) in 324 patients, with a PPV of 96% (95% CI, 0.79-1.00), NPV of 78% (95% CI, 0.73-0.83), accuracy of 79% (95% CI, 0.74-0.84), and AUC of 0.63. Among the 145 patients with available serum CA19-9 levels, CA19-9 at a threshold of ≥44 U/mL demonstrated a sensitivity of 66% (95% CI, 0.52-0.78) and specificity of 63% (95% CI, 0.52-0.73), with a PPV of 51% (95% CI, 0.38-0.63), NPV of 76% (95% CI, 0.65-0.85), accuracy of 64% (95% CI, 0.56-0.72), and AUC of 0.65. CA19-9 at a threshold of >129 U/mL demonstrated a sensitivity of 45% (95% CI, 0.32-

0.60) and specificity of 88% (95% CI, 0.80-0.94), with a PPV of 69% (95% CI, 0.51-0.83), NPV of 74% (95% CI, 0.64-0.82), accuracy of 72% (95% CI, 0.64-0.80), and AUC of 0.67. BiliSeqV2/V3 demonstrated significantly higher sensitivity compared to pathologic evaluation alone (p<0.001), CA19-9≥44 U/mL (p=0.008), and CA19-9>129 U/mL (p<0.001). BiliSeqV2/V3 demonstrated significantly higher specificity compared to CA19-9≥44 U/mL (p<0.001). BiliSeqV2/V3 also demonstrated significantly higher accuracy compared to pathologic evaluation alone (p<0.001), CA19-9≥44 U/mL (p<0.001), and CA19-9>129 U/mL (p<0.001).

The combination of pathologic evaluation and BiliSeqV2/V3 had a sensitivity of 89% (95% CI, 0.80-0.94) and specificity of 98% (95% CI, 0.95-0.99), with a PPV of 94% (95% CI, 0.87-0.98), NPV of 96% (95% CI, 0.93-0.98), accuracy of 95% (95% CI, 0.93-0.97), and AUC of 0.93. No significant difference in sensitivity (p=0.25), specificity (p=1.0), or accuracy (p=0.63) was observed between BiliSeqV2/V3 alone and the combination of pathologic evaluation and BiliSeqV2/V3. The combination of pathologic evaluation and BiliSeqV2/V3 demonstrated significantly higher sensitivity compared to pathologic evaluation alone (p<0.001) and CA19-9>129 U/mL (p<0.001).

Germline Alteration Analysis

Among the 1,863 patients evaluated by BiliSeqV3 testing, which included germline alteration analysis, 69 (3.7%) patients were found to harbor germline alterations. Patients with germline alterations ranged in age from 16 to 86 years (mean, 65.0 years; median, 68.0 years). The subgroup consisted of 40 of 69 (58.0%) male patients and 29 of 69 (42.0%) female patients. Regarding race and ethnicity, the majority of patients were Caucasian (56 of 69, 81.2%), followed by Black (5 of 69, 7.2%), Hispanic (4 of 69, 5.8%), and Asian (2 of 69, 2.9%).

A documented history of PSC was noted for 10 of 69 (14.5%) patients with germline alterations, while 59 of 69 (85.5%) patients did not have PSC. Indeterminate strictures were present in 20 of 69 (29.0%) patients. Serum CA19-9 levels were available for 33 of 69 (47.8%)

patients and ranged from 3.2 to 13,947.8 U/mL (mean, 1,555.9 U/mL; median, 95.8 U/mL). The biliary duct strictures in patients with germline alterations were located within the following anatomic sites: distal in 33 of 69 (47.8%) patients, intrahepatic/hilar in 26 of 69 (37.7%) patients, and middle bile duct in 10 of 69 (14.5%) patients.

A total of 94 biliary specimens from 69 patients with germline alterations were submitted for BiliSeqV3 molecular testing, consisting of 62 brushings, 26 biopsies, and 6 bile specimens. Positive BiliSeqV3 results, indicating the presence of a somatic genomic alteration, were detected in 31 of 62 (50.0%) brushing specimens, 20 of 26 (76.9%) biopsy specimens, and 4 of 6 (66.7%) bile specimens. Follow-up was available for 66 of 69 (95.7%) patients with germline alterations. The duration of follow-up ranged from 1 to 43 months (mean, 16.8 months; median, 15.0 months). Among patients with a benign final diagnosis, including benign cholangiopathy and IgG4 disease (n=24), the duration of follow-up ranged from 12 to 43 months (mean, 21.3 months; median, 18.5 months). Among patients with a neoplastic final diagnosis (n=42), the duration of follow-up ranged from 1 to 38 months (mean, 14.2 months; median, 12.5 months). The final diagnoses among patients with germline alterations and available follow-up included benign cholangiopathy (24 of 66, 36.4%), intrahepatic cholangiocarcinoma (13 of 66, 19.7%), extrahepatic cholangiocarcinoma (12 of 66, 18.2%), pancreatic ductal adenocarcinoma (8 of 66, 12.1%), ampullary adenocarcinoma (7 of 66, 10.6%), metastatic lung adenocarcinoma (1 of 66, 1.5%), and high-grade dysplasia (1 of 66, 1.5%).

Diagnostic performance was evaluated for BiliSeqV3, pathologic evaluation, and serum CA19-9 in detecting neoplasia among patients with germline alterations and available follow-up (**Supplementary Figure 13**). BiliSeqV3 demonstrated a sensitivity of 86% (95% CI, 0.71-0.95) and specificity of 100% (95% CI, 0.86-1.00) for the detection of neoplasia in 66 patients with follow-up, with a positive predictive value (PPV) of 100% (95% CI, 0.90-1.00), negative predictive value (NPV) of 80% (95% CI, 0.61-0.92), and accuracy of 91% (95% CI, 0.81-0.97). The area under the receiver operating characteristic curve (AUC) was 0.93 for BiliSeqV3. Pathologic

evaluation alone demonstrated a sensitivity of 50% (95% CI, 0.34-0.66) and specificity of 100% (95% CI, 0.86-1.00), with a PPV of 100% (95% CI, 0.84-1.00), NPV of 53% (95% CI, 0.38-0.68), accuracy of 68% (95% CI, 0.56-0.79), and AUC of 0.75. Among the 33 patients with available serum CA19-9 levels, CA19-9 at a threshold of ≥44 U/mL demonstrated a sensitivity of 72% (95% CI, 0.51-0.88) and specificity of 62% (95% CI, 0.24-0.91), with a PPV of 86% (95% CI, 0.64-0.97), NPV of 42% (95% CI, 0.15-0.72), accuracy of 70% (95% CI, 0.51-0.84), and AUC of 0.67. CA19-9 at a threshold of >129 U/mL demonstrated a sensitivity of 60% (95% CI, 0.39-0.79) and specificity of 88% (95% CI, 0.47-1.00), with a PPV of 94% (95% CI, 0.70-1.00), NPV of 41% (95% CI, 0.18-0.67), accuracy of 67% (95% CI, 0.48-0.82), and AUC of 0.74. BiliSeqV3 demonstrated significantly higher sensitivity compared to pathologic evaluation alone (p=0.002). BiliSeqV3 also demonstrated significantly higher accuracy compared to pathologic evaluation alone (p=0.002) and CA19-9>129 U/mL (p=0.04).

The combination of pathologic evaluation and BiliSeqV3 demonstrated a sensitivity of 93% (95% CI, 0.81-0.99) and specificity of 100% (95% CI, 0.86-1.00), with a PPV of 100% (95% CI, 0.91-1.00), NPV of 89% (95% CI, 0.71-0.98), accuracy of 95% (95% CI, 0.87-0.99), and AUC of 0.96. The combination of pathologic evaluation and BiliSeqV3 demonstrated significantly higher sensitivity compared to pathologic evaluation alone (p<0.001) and CA19-9>129 U/mL (p=0.02). The combination of pathologic evaluation and BiliSeqV3 had significantly higher accuracy compared to pathologic evaluation alone (p<0.001) and CA19-9>129 U/mL (p=0.01). No significant difference in sensitivity (p=0.25), specificity (p=1.0), or accuracy (p=0.25) was observed between BiliSeqV3 alone and the combination of pathologic evaluation and BiliSeqV3.

Race and Ethnicity Analysis

Among the 2,080 patients evaluated by BiliSeqV2/V3 testing, race and ethnicity data were available for 2,006 (96.4%) patients (detailed information in Supplementary Table 4). The

distribution included 1,603 (77.1%) Caucasian patients, 204 (9.8%) Black patients, 127 (6.1%) Hispanic patients, and 72 (3.5%) Asian patients.

Caucasian patients included 186 from the BiliSeqV2 cohort and 1,417 from the BiliSeqV3 cohort. Patients ranged in age from 10 to 97 years (mean, 66.4 years; median, 69.0 years) and consisted of 898 of 1,603 (56.0%) male and 705 of 1,603 (44.0%) female patients. A documented history of PSC was noted for 238 of 1,603 (14.8%) patients, and indeterminate strictures were present in 478 of 1,603 (29.8%) patients. The biliary duct strictures were located within the following anatomic sites: distal in 832 of 1,603 (51.9%) patients, intrahepatic/hilar in 579 of 1,603 (36.1%) patients, middle bile duct in 171 of 1,603 (10.7%) patients, and entire bile duct in 13 of 1,603 (0.8%) patients. Positive BiliSeqV2/V3 results were detected in 686 of 1,459 (47.0%) brushing specimens and 369 of 704 (52.4%) biopsy specimens.

Follow-up was available for 1,544 patients. Among patients with a benign final diagnosis (n=637), the duration of follow-up ranged from 12 to 60 months (mean, 23.9 months; median, 21.0 months). Among patients with a neoplastic final diagnosis (n=896), the duration of follow-up ranged from 1 to 57 months (mean, 12.1 months; median, 9.0 months). The final diagnoses included benign cholangiopathy (609 of 1,544, 39.4%), pancreatic ductal adenocarcinoma (274 of 1,544, 17.7%), intrahepatic cholangiocarcinoma (231 of 1,544, 15.0%), extrahepatic cholangiocarcinoma (208 of 1,544, 13.5%), ampullary adenocarcinoma (57 of 1,544, 3.7%), perihilar cholangiocarcinoma (46 of 1,544, 3.0%), gallbladder adenocarcinoma (22 of 1,544, 1.4%), and IgG4 disease (22 of 1,544, 1.4%).

Diagnostic performance was evaluated for BiliSeqV2/V3, pathologic evaluation, and serum CA19-9 in detecting neoplasia among Caucasian patients with available follow-up (**Supplementary Figure 14**). BiliSeqV2/V3 demonstrated a sensitivity of 82% (95% CI, 0.79-0.84) and specificity of 99% (95% CI, 0.98-0.99) for the detection of neoplasia in 1,544 patients with follow-up, with a positive predictive value (PPV) of 99% (95% CI, 0.98-1.00), negative predictive value (NPV) of 79% (95% CI, 0.76-0.82), and accuracy of 89% (95% CI, 0.87-0.90). The area

under the receiver operating characteristic curve (AUC) was 0.90 for BiliSeqV2/V3. Pathologic evaluation alone demonstrated a sensitivity of 44% (95% CI, 0.41-0.48) and specificity of 99% (95% CI, 0.98-0.99) in 1,542 patients, with a PPV of 98% (95% CI, 0.96-0.99), NPV of 55% (95% CI, 0.52-0.58), accuracy of 67% (95% CI, 0.64-0.69), and AUC of 0.72. Among the 823 patients with available serum CA19-9 levels, CA19-9 at a threshold of ≥44 U/mL demonstrated a sensitivity of 71% (95% CI, 0.67-0.74) and specificity of 55% (95% CI, 0.49-0.62), with a PPV of 80% (95% CI, 0.76-0.83), NPV of 42% (95% CI, 0.37-0.48), accuracy of 66% (95% CI, 0.63-0.69), and AUC of 0.63. CA19-9 at a threshold of >129 U/mL demonstrated a sensitivity of 55% (95% CI, 0.51-0.59) and specificity of 77% (95% CI, 0.71-0.82), with a PPV of 86% (95% CI, 0.82-0.89), NPV of 40% (95% CI, 0.35-0.45), accuracy of 61% (95% CI, 0.58-0.64), and AUC of 0.66. BiliSeqV2/V3 demonstrated significantly higher sensitivity compared to pathologic evaluation alone (p<0.001), CA19-9≥44 U/mL (p<0.001), and CA19-9>129 U/mL (p<0.001). BiliSeqV2/V3 demonstrated significantly higher specificity compared to CA19-9≥44 U/mL (p<0.001) and CA19-9>129 U/mL (p<0.001). BiliSeqV2/V3 also demonstrated significantly higher accuracy compared to pathologic evaluation alone (p<0.001), CA19-9≥44 U/mL (p<0.001), and CA19-9>129 U/mL (p<0.001). The combination of pathologic evaluation and BiliSeqV2/V3 demonstrated a sensitivity of 88% (95% CI, 0.85-0.90) and specificity of 97% (95% CI, 0.96-0.99), with a PPV of 98% (95% CI, 0.97-0.99), NPV of 85% (95% CI, 0.82-0.87), accuracy of 92% (95% CI, 0.90-0.93), and AUC of 0.93. The combination of pathologic evaluation and BiliSeqV2/V3 demonstrated significantly higher sensitivity compared to BiliSeqV2/V3 alone (p<0.001), pathologic evaluation alone (p<0.001), CA19-9≥44 U/mL (p<0.001), and CA19-9>129 U/mL (p<0.001). Specificity was significantly lower for the combination of pathologic evaluation and BiliSeqV2/V3 compared to BiliSeqV2/V3 alone (p=0.008). The combination of pathologic evaluation and BiliSeqV2/V3 demonstrated significantly higher accuracy compared to BiliSeqV2/V3 alone (p<0.001), pathologic evaluation alone (p<0.001), CA19-9≥44 U/mL (p<0.001), and CA19-9>129 U/mL (p<0.001).

Black patients included 20 from the BiliSeqV2 cohort and 184 from the BiliSeqV3 cohort. Patients ranged in age from 19 to 96 years (mean, 61.1 years; median, 63.0 years) and consisted of 120 of 204 (58.8%) male and 84 of 204 (41.2%) female patients. A documented history of PSC was noted for 49 of 204 (24.0%) patients, a higher prevalence than in the overall cohort. Indeterminate strictures were present in 48 of 204 (23.5%) patients. The biliary duct strictures were located within the following anatomic sites: intrahepatic/hilar in 88 of 204 (43.1%) patients, distal in 78 of 204 (38.2%) patients, middle bile duct in 33 of 204 (16.2%) patients, and entire bile duct in 4 of 204 (2.0%) patients. Positive BiliSeqV2/V3 results were detected in 76 of 184 (41.3%) brushing specimens and 32 of 71 (45.1%) biopsy specimens. Follow-up was available for 194 patients. Among patients with a benign final diagnosis (n=98), the duration of follow-up ranged from 12 to 59 months (mean, 25.3 months; median, 21.0 months). Among patients with a neoplastic final diagnosis (n=92), the duration of follow-up ranged from 1 to 57 months (mean, 12.6 months; median, 10.0 months). The final diagnoses included benign cholangiopathy (95 of 194, 49.0%), intrahepatic cholangiocarcinoma (28 of 194, 14.4%), extrahepatic cholangiocarcinoma (25 of 194, 12.9%), pancreatic ductal adenocarcinoma (19 of 194, 9.8%), perihilar cholangiocarcinoma (6 of 194, 3.1%), IgG4 disease (6 of 194, 3.1%), and ampullary adenocarcinoma (5 of 194, 2.6%).

Diagnostic performance was evaluated for BiliSeqV2/V3, pathologic evaluation, and serum CA19-9 in detecting neoplasia among Black patients with available follow-up (**Supplementary Figure 15**). BiliSeqV2/V3 demonstrated a sensitivity of 83% (95% CI, 0.74-0.90) and specificity of 96% (95% CI, 0.90-0.99) for the detection of neoplasia in 194 patients with follow-up, with a PPV of 95% (95% CI, 0.88-0.99), NPV of 85% (95% CI, 0.77-0.91), and accuracy of 90% (95% CI, 0.85-0.94). The AUC was 0.90 for BiliSeqV2/V3. Pathologic evaluation alone demonstrated a sensitivity of 42% (95% CI, 0.32-0.52) and specificity of 99% (95% CI, 0.94-1.00) in 192 patients, with a PPV of 98% (95% CI, 0.87-1.00), NPV of 63% (95% CI, 0.55-0.71), accuracy of 70% (95% CI, 0.63-0.77), and AUC of 0.70. Among the 67 patients with available

serum CA19-9 levels, CA19-9 at a threshold of ≥44 U/mL demonstrated a sensitivity of 51% (95% CI, 0.35-0.67) and specificity of 42% (95% CI, 0.23-0.63), with a PPV of 58% (95% CI, 0.41-0.74), NPV of 35% (95% CI, 0.19-0.55), accuracy of 48% (95% CI, 0.35-0.60), and AUC of 0.47. CA19-9 at a threshold of >129 U/mL demonstrated a sensitivity of 44% (95% CI, 0.28-0.60) and specificity of 85% (95% CI, 0.65-0.96), with a PPV of 82% (95% CI, 0.60-0.95), NPV of 49% (95% CI, 0.34-0.64), accuracy of 60% (95% CI, 0.47-0.72), and AUC of 0.64. BiliSeqV2/V3 demonstrated significantly higher sensitivity compared to pathologic evaluation alone (p<0.001), CA19-9≥44 U/mL (p=0.001), and CA19-9>129 U/mL (p<0.001). BiliSeqV2/V3 demonstrated significantly higher specificity compared to CA19-9≥44 U/mL (p<0.001). BiliSeqV2/V3 also demonstrated significantly higher accuracy compared to pathologic evaluation alone (p<0.001), CA19-9≥44 U/mL (p<0.001), and CA19-9>129 U/mL (p<0.001). The combination of pathologic evaluation and BiliSeqV2/V3 demonstrated a sensitivity of 89% (95% CI, 0.80-0.94) and specificity of 95% (95% CI, 0.88-0.98), with a PPV of 94% (95% CI, 0.88-0.98), NPV of 89% (95% CI, 0.82-0.95), accuracy of 92% (95% CI, 0.87-0.95), and AUC of 0.92. The combination of pathologic evaluation and BiliSeqV2/V3 demonstrated significantly higher sensitivity compared to pathologic evaluation alone (p<0.001) and CA19-9≥44 U/mL (p=0.001). No significant difference in sensitivity (p=0.06) or accuracy (p=0.22) was observed between BiliSeqV2/V3 alone and the combination of pathologic evaluation and BiliSeqV2/V3.

Hispanic patients were exclusively from the BiliSeqV3 cohort. Patients ranged in age from 8 to 92 years (mean, 59.4 years; median, 60.0 years), representing the youngest racial/ethnic subgroup. The gender distribution was 61 of 127 (48.0%) male and 66 of 127 (52.0%) female patients, representing the only subgroup with a female predominance. A documented history of PSC was noted for 18 of 127 (14.2%) patients, and indeterminate strictures were present in 12 of 127 (9.4%) patients, the lowest prevalence among racial/ethnic subgroups. The biliary duct strictures were located within the following anatomic sites: intrahepatic/hilar in 58 of 127 (45.7%) patients, distal in 47 of 127 (37.0%) patients, middle bile duct in 20 of 127 (15.7%) patients, and

entire bile duct in 1 of 127 (0.8%) patients. Positive BiliSeqV2/V3 results were detected in 34 of 119 (28.6%) brushing specimens and 9 of 29 (31.0%) biopsy specimens, the lowest positivity rates among racial/ethnic subgroups. Follow-up was available for 114 patients. Among patients with a benign final diagnosis (n=67), the duration of follow-up ranged from 12 to 43 months (mean, 20.9 months; median, 19.0 months). Among patients with a neoplastic final diagnosis (n=47), the duration of follow-up ranged from 1 to 27 months (mean, 10.6 months; median, 9.0 months). The final diagnoses included benign cholangiopathy (66 of 114, 57.9%), intrahepatic cholangiocarcinoma (19 of 114, 16.7%), extrahepatic cholangiocarcinoma (11 of 114, 9.6%), ampullary adenocarcinoma (6 of 114, 5.3%), perihilar cholangiocarcinoma (5 of 114, 4.4%), and pancreatic ductal adenocarcinoma (3 of 114, 2.6%).

Diagnostic performance was evaluated for BiliSeqV2/V3, pathologic evaluation, and serum CA19-9 in detecting neoplasia among Hispanic patients with available follow-up (**Supplementary Figure 16**). BiliSeqV2/V3 demonstrated a sensitivity of 74% (95% CI, 0.60-0.86) and specificity of 100% (95% CI, 0.95-1.00) for the detection of neoplasia in 114 patients with follow-up, with a PPV of 100% (95% CI, 0.90-1.00), NPV of 85% (95% CI, 0.75-0.92), and accuracy of 89% (95% CI, 0.82-0.94). The AUC was 0.87 for BiliSeqV2/V3. Pathologic evaluation alone demonstrated a sensitivity of 38% (95% CI, 0.25-0.54) and specificity of 100% (95% CI, 0.95-1.00), with a PPV of 100% (95% CI, 0.81-1.00), NPV of 70% (95% CI, 0.60-0.79), accuracy of 75% (95% CI, 0.66-0.82), and AUC of 0.69. Among the 10 patients with available serum CA19-9 levels, CA19-9 at a threshold of ≥44 U/mL demonstrated a sensitivity of 83% (95% CI, 0.36-1.00) and specificity of 50% (95% CI, 0.07-0.93), with a PPV of 71% (95% CI, 0.29-0.96), NPV of 67% (95% CI, 0.09-0.99), accuracy of 70% (95% CI, 0.35-0.93), and AUC of 0.67. CA19-9 at a threshold of >129 U/mL demonstrated a sensitivity of 67% (95% CI, 0.22-0.96) and specificity of 75% (95% CI, 0.19-0.99), with a PPV of 80% (95% CI, 0.28-0.99), NPV of 60% (95% CI, 0.15-0.95), accuracy of 70% (95% CI, 0.35-0.93), and AUC of 0.71. BiliSeqV2/V3 demonstrated significantly higher sensitivity compared to pathologic evaluation alone (p=0.003). BiliSeqV2/V3

also demonstrated significantly higher accuracy compared to pathologic evaluation alone (p=0.003). Statistical comparisons with CA19-9were limited by the small sample size. The combination of pathologic evaluation and BiliSeqV2/V3 demonstrated a sensitivity of 89% (95% CI, 0.77-0.96) and specificity of 100% (95% CI, 0.95-1.00), with a PPV of 100% (95% CI, 0.92-1.00), NPV of 93% (95% CI, 0.85-0.98), accuracy of 96% (95% CI, 0.90-0.99), and AUC of 0.95. The combination of pathologic evaluation and BiliSeqV2/V3 demonstrated significantly higher sensitivity compared to BiliSeqV2/V3 alone (p=0.02) and pathologic evaluation alone (p<0.001). The combination of pathologic evaluation and BiliSeqV2/V3 also demonstrated significantly higher accuracy compared to BiliSeqV2/V3 alone (p=0.02) and pathologic evaluation alone (p<0.001).

Asian patients included 7 from the BiliSeqV2 cohort and 65 from the BiliSeqV3 cohort. Patients ranged in age from 12 to 92 years (mean, 64.5 years; median, 67.5 years) and consisted of 44 of 72 (61.1%) male and 28 of 72 (38.9%) female patients. A documented history of PSC was noted for 17 of 72 (23.6%) patients, and indeterminate strictures were present in 13 of 72 (18.1%) patients. The biliary duct strictures were located within the following anatomic sites: intrahepatic/hilar in 31 of 72 (43.1%) patients, distal in 26 of 72 (36.1%) patients, middle bile duct in 14 of 72 (19.4%) patients, and entire bile duct in 1 of 72 (1.4%) patients. Positive BiliSeqV2/V3 results were detected in 27 of 67 (40.3%) brushing specimens and 16 of 23 (69.6%) biopsy specimens, with biopsy positivity representing the highest rate among racial/ethnic subgroups. Follow-up was available for 69 patients. Among patients with a benign final diagnosis (n=31), the duration of follow-up ranged from 12 to 43 months (mean, 21.9 months; median, 21.0 months). Among patients with a neoplastic final diagnosis (n=38), the duration of follow-up ranged from 2 to 59 months (mean, 16.7 months; median, 16.5 months). The final diagnoses included benign cholangiopathy (29 of 69, 42.0%), intrahepatic cholangiocarcinoma (13 of 69, 18.8%), extrahepatic cholangiocarcinoma (9 of 69, 13.0%), ampullary adenocarcinoma (4 of 69, 5.8%),

gallbladder adenocarcinoma (4 of 69, 5.8%), and pancreatic ductal adenocarcinoma (4 of 69, 5.8%).

Diagnostic performance was evaluated for BiliSeqV2/V3, pathologic evaluation, and serum CA19-9 in detecting neoplasia among Asian patients with available follow-up (**Supplementary Figure 17**). BiliSeqV2/V3 demonstrated a sensitivity of 79% (95% CI, 0.63-0.90) and specificity of 97% (95% CI, 0.83-1.00) for the detection of neoplasia in 69 patients with follow-up, with a PPV of 97% (95% CI, 0.83-1.00), NPV of 79% (95% CI, 0.63-0.90), and accuracy of 87% (95% CI, 0.77-0.94). The AUC was 0.88 for BiliSeqV2/V3. Pathologic evaluation alone demonstrated a sensitivity of 42% (95% CI, 0.26-0.59) and specificity of 100% (95% CI, 0.89-1.00), with a PPV of 100% (95% CI, 0.79-1.00), NPV of 58% (95% CI, 0.44-0.72), accuracy of 68% (95% CI, 0.56-0.79), and AUC of 0.71. Among the 18 patients with available serum CA19-9 levels, CA19-9 at a threshold of ≥44 U/mL demonstrated a sensitivity of 57% (95% CI, 0.29-0.82) and specificity of 75% (95% CI, 0.19-0.99), with a PPV of 89% (95% CI, 0.52-1.00), NPV of 33% (95% CI, 0.07-0.70), accuracy of 61% (95% CI, 0.36-0.83), and AUC of 0.66. CA19-9 at a threshold of >129 U/mL demonstrated a sensitivity of 29% (95% CI, 0.08-0.58) and specificity of 75% (95% CI, 0.19-0.99), with a PPV of 80% (95% CI, 0.28-0.99), NPV of 23% (95% CI, 0.05-0.54), accuracy of 39% (95% CI, 0.17-0.64), and AUC of 0.52. BiliSeqV2/V3 demonstrated significantly higher sensitivity compared to pathologic evaluation alone (p=0.001) and CA19-9>129 U/mL (p=0.004). BiliSeqV2/V3 also demonstrated significantly higher accuracy compared to pathologic evaluation alone (p=0.004), CA19-9≥44 U/mL (p=0.03), and CA19-9>129 U/mL (p=0.002). The combination of pathologic evaluation and BiliSeqV2/V3 demonstrated a sensitivity of 84% (95% CI, 0.69-0.94) and specificity of 97% (95% CI, 0.83-1.00), with a PPV of 97% (95% CI, 0.84-1.00), NPV of 83% (95% CI, 0.67-0.94), accuracy of 90% (95% CI, 0.80-0.96), and AUC of 0.90. The combination of pathologic evaluation and BiliSeqV2/V3 demonstrated significantly higher sensitivity compared to pathologic evaluation alone (p<0.001) and CA19-9>129 U/mL (p=0.004). The combination of pathologic evaluation and BiliSeqV2/V3 also demonstrated

significantly higher accuracy compared to pathologic evaluation alone (p<0.001) and CA19-9>129 U/mL (p=0.002). No significant difference in sensitivity (p=0.50) or accuracy (p=0.50) was observed between BiliSeqV2/V3 alone and the combination of pathologic evaluation and BiliSeqV2/V3.

Repeat Testing Analysis

Among the 2,080 patients evaluated by BiliSeqV2/V3 testing, 262 (12.6%) patients underwent repeat BiliSeqV2/V3 testing with multiple ERCP procedures (detailed information in Supplementary Table 4). A total of 610 BiliSeqV2/V3 tests were performed in these 262 patients, with the number of ERCP procedures per patient ranging from 2 to 6 (mean, 2.3 tests; median, 2.0 tests). The distribution of repeat testing included 195 of 262 (74.4%) patients with 2 ERCPs, 54 of 262 (20.6%) patients with 3 ERCPs, 9 of 262 (3.4%) patients with 4 ERCPs, 2 of 262 (0.8%) patients with 5 ERCPs, and 2 of 262 (0.8%) patients with 6 ERCPs. The majority of tests were performed using BiliSeqV3 (514 of 610, 84.3%), with the remainder using BiliSeqV2 (96 of 610, 15.7%).

Patients ranged in age from 13 to 91 years (mean, 63.4 years; median, 66.0 years). The subgroup consisted of 146 of 262 (55.7%) male patients and 116 of 262 (44.3%) female patients. Regarding race and ethnicity, the majority of patients were Caucasian (204 of 262, 77.9%), followed by Black (33 of 262, 12.6%), Asian (12 of 262, 4.6%), Hispanic (8 of 262, 3.1%), and not specified (5 of 262, 1.9%). PSC was noted for 83 of 262 (31.7%) patients who underwent repeat testing, a notably higher prevalence compared to the overall cohort (16.4%). Indeterminate strictures were present in 135 of 262 (51.5%) patients. Serum CA19-9 levels were available for 129 of 262 (49.2%) patients and ranged from 0.8 to 39,010 U/mL (mean, 784.0 U/mL; median, 48.7 U/mL). The biliary duct strictures were located within the following anatomic sites: distal in 119 of 262 (45.4%) patients, intrahepatic/hilar in 116 of 262 (44.3%) patients, middle bile duct in 23 of 262 (8.8%) patients, entire bile duct in 2 of 262 (0.8%) patients, and multiple locations in 2

of 262 (0.8%) patients. Germline alteration testing identified germline alterations in 13 of 262 (5.0%) patients, while 150 of 262 (57.3%) patients did not harbor germline alterations, and 99 of 262 (37.8%) patients were not tested or had unspecified germline status.

Across all 610 BiliSeqV2/V3 tests performed in the repeat testing subgroup, specimens included 532 brushings, 288 biopsies, and 48 bile/aspiration specimens. Positive BiliSeqV2/V3 results, indicating the presence of a genomic alteration, were detected in 170 of 552 (30.8%) brushing specimens, 98 of 323 (30.3%) biopsy specimens, and 16 of 48 (33.3%) bile/aspiration specimens tested. At the patient level, 105 of 262 (40.1%) patients had at least one positive BiliSeqV2/V3 test across their repeat testing episodes, while 157 of 262 (59.9%) patients had negative results on all tests.

Follow-up was available for 260 of 262 (99.2%) patients who underwent repeat testing. The duration of follow-up ranged from 3 to 60 months (mean, 19.2 months; median, 15.0 months). Follow-up consisted of pathologic confirmation for 101 of 260 (38.8%) patients and clinicoradiographic impression for 159 of 260 (61.2%) patients. Among the 159 patients with clinicoradiographic follow-up, the duration of surveillance ranged from 5 to 60 months (mean, 24.2 months; median, 21.0 months). Pathologic confirmation was achieved through various methods including Whipple resection (40 of 260, 15.4%), hepatectomy (31 of 260, 11.9%), hepatectomy and bile duct resection (8 of 260, 3.1%), bile duct resection (7 of 260, 2.7%), liver core needle biopsy (5 of 260, 1.9%), pancreatic EUS-FNA (3 of 260, 1.2%), liver transplantation (2 of 260, 0.8%), gallbladder and bile duct resection (2 of 260, 0.8%), concurrent ERCP biopsy (2 of 260, 0.8%), and autopsy (1 of 260, 0.4%).

Among patients with a benign final diagnosis, including benign cholangiopathy and IgG4 disease (n=149), the duration of follow-up ranged from 12 to 60 months (mean, 25.9 months; median, 23.0 months). Among patients with a neoplastic final diagnosis (n=111), the duration of follow-up ranged from 3 to 19 months (mean, 10.2 months; median, 10.0 months). The final diagnoses among the 260 patients with available follow-up included benign cholangiopathy (135

of 260, 51.9%), intrahepatic cholangiocarcinoma (38 of 260, 14.6%), extrahepatic cholangiocarcinoma (29 of 260, 11.2%), IgG4 disease (14 of 260, 5.4%), pancreatic ductal adenocarcinoma (14 of 260, 5.4%), perihilar cholangiocarcinoma (7 of 260, 2.7%), low-grade dysplasia (6 of 260, 2.3%), ampullary adenocarcinoma (4 of 260, 1.5%), high-grade dysplasia (3 of 260, 1.2%), B-cell lymphoma (3 of 260, 1.2%), gallbladder adenocarcinoma (2 of 260, 0.8%), metastatic lung adenocarcinoma (2 of 260, 0.8%), metastatic colonic adenocarcinoma (1 of 260, 0.4%), metastatic colorectal adenocarcinoma (1 of 260, 0.4%), and metastatic renal cell carcinoma (1 of 260, 0.4%). Overall, 149 of 260 (57.3%) patients with available follow-up had a benign final diagnosis, while 111 of 260 (42.7%) patients had a neoplastic final diagnosis.

Diagnostic performance was evaluated for BiliSeqV2/V3, pathologic evaluation, and serum CA19-9 in detecting neoplasia among patients who underwent repeat testing, stratified by testing episode. At initial testing (1st ERCP), BiliSeqV2/V3 demonstrated a sensitivity of 72% (95% CI, 0.63-0.80) and specificity of 97% (95% CI, 0.93-0.99) for the detection of neoplasia in 260 patients with follow-up, with a positive predictive value (PPV) of 95% (95% CI, 0.88-0.99), negative predictive value (NPV) of 82% (95% CI, 0.76-0.88), and accuracy of 87% (95% CI, 0.82-0.90) (**Supplementary Figure 18**). The area under the receiver operating characteristic curve (AUC) was 0.85 for BiliSeqV2/V3 at initial testing. Pathologic evaluation alone at initial testing demonstrated a sensitivity of 14% (95% CI, 0.08-0.21) and specificity of 99% (95% CI, 0.96-1.00) in 259 patients, with a PPV of 94% (95% CI, 0.70-1.00), NPV of 60% (95% CI, 0.54-0.67), accuracy of 63% (95% CI, 0.56-0.68), and AUC of 0.56. Among the 128 patients with available serum CA19-9 levels, CA19-9 at a threshold of ≥44 U/mL demonstrated a sensitivity of 56% (95% CI, 0.43-0.68) and specificity of 48% (95% CI, 0.35-0.61), with a PPV of 54% (95% CI, 0.41-0.66), NPV of 51% (95% CI, 0.37-0.64), accuracy of 52% (95% CI, 0.43-0.61), and AUC of 0.52. CA19-9 at a threshold of >129 U/mL demonstrated a sensitivity of 39% (95% CI, 0.28-0.52) and specificity of 85% (95% CI, 0.74-0.93), with a PPV of 74% (95% CI, 0.57-0.88), NPV of 57% (95% CI, 0.46-0.67), accuracy of 62% (95% CI, 0.53-0.70), and AUC of 0.62. At initial testing,

BiliSeqV2/V3 demonstrated significantly higher sensitivity compared to pathologic evaluation alone (p<0.001) and CA19-9>129 U/mL (p<0.001). BiliSeqV2/V3 demonstrated significantly higher specificity compared to CA19-9≥44 U/mL (p<0.001). BiliSeqV2/V3 also demonstrated significantly higher accuracy compared to pathologic evaluation alone (p<0.001), CA19-9≥44 U/mL (p<0.001), and CA19-9>129 U/mL (p<0.001). The combination of pathologic evaluation and BiliSeqV2/V3 at initial testing demonstrated a sensitivity of 75% (95% CI, 0.66-0.83) and specificity of 97% (95% CI, 0.92-0.99), with a PPV of 94% (95% CI, 0.87-0.98), NPV of 84% (95% CI, 0.77-0.89), accuracy of 87% (95% CI, 0.83-0.91), and AUC of 0.86. No significant difference in sensitivity (p=0.25), specificity (p=1.0), or accuracy (p=0.63) was observed between BiliSeqV2/V3 alone and the combination of pathologic evaluation and BiliSeqV2/V3 at initial testing.

At first repeat testing (2nd ERCP), BiliSeqV2/V3 demonstrated a sensitivity of 83% (95% CI, 0.75-0.89) and specificity of 95% (95% CI, 0.91-0.98), with a PPV of 93% (95% CI, 0.86-0.97), NPV of 88% (95% CI, 0.82-0.93), accuracy of 90% (95% CI, 0.86-0.93), and AUC of 0.89 (**Supplementary Figure 19**). Pathologic evaluation alone at first repeat testing demonstrated a sensitivity of 30% (95% CI, 0.21-0.39) and specificity of 99% (95% CI, 0.96-1.00), with a PPV of 97% (95% CI, 0.85-1.00), NPV of 65% (95% CI, 0.59-0.72), accuracy of 70% (95% CI, 0.64-0.75), and AUC of 0.65. At first repeat testing, BiliSeqV2/V3 demonstrated significantly higher sensitivity compared to pathologic evaluation alone (p<0.001), CA19-9≥44 U/mL (p=0.002), and CA19-9>129 U/mL (p<0.001). BiliSeqV2/V3 demonstrated significantly higher specificity compared to CA19-9≥44 U/mL (p<0.001) and CA19-9>129 U/mL (p=0.02). BiliSeqV2/V3 also demonstrated significantly higher accuracy compared to pathologic evaluation alone (p<0.001), CA19-9≥44 U/mL (p<0.001), and CA19-9>129 U/mL (p<0.001). The combination of pathologic evaluation and BiliSeqV2/V3 at first repeat testing demonstrated a sensitivity of 90% (95% CI, 0.83-0.95) and specificity of 95% (95% CI, 0.90-0.98), with a PPV of 93% (95% CI, 0.86-0.97), NPV of 93% (95% CI, 0.87-0.96), accuracy of 93% (95% CI, 0.89-0.96), and AUC of 0.92. The combination

demonstrated significantly higher sensitivity (p=0.008) and accuracy (p=0.04) compared to BiliSeqV2/V3 alone at first repeat testing.

At second repeat testing (3rd ERCP), BiliSeqV2/V3 demonstrated a sensitivity of 85% (95% CI, 0.77-0.91) and specificity of 93% (95% CI, 0.87-0.96), with a PPV of 90% (95% CI, 0.82-0.95), NPV of 89% (95% CI, 0.83-0.93), accuracy of 89% (95% CI, 0.85-0.93), and AUC of 0.89 (**Supplementary Figure 20**). Pathologic evaluation alone at second repeat testing demonstrated a sensitivity of 32% (95% CI, 0.23-0.41) and specificity of 98% (95% CI, 0.94-1.00), with a PPV of 92% (95% CI, 0.79-0.98), NPV of 66% (95% CI, 0.59-0.72), accuracy of 70% (95% CI, 0.64-0.75), and AUC of 0.65. At second repeat testing, BiliSeqV2/V3 demonstrated significantly higher sensitivity compared to pathologic evaluation alone (p<0.001), CA19-9≥44 U/mL (p<0.001), and CA19-9>129 U/mL (p<0.001). BiliSeqV2/V3 demonstrated significantly higher specificity compared to CA19-9≥44 U/mL (p<0.001). BiliSeqV2/V3 also demonstrated significantly higher accuracy compared to pathologic evaluation alone (p<0.001), CA19-9≥44 U/mL (p<0.001), and CA19-9>129 U/mL (p<0.001). The combination of pathologic evaluation and BiliSeqV2/V3 at second repeat testing demonstrated a sensitivity of 93% (95% CI, 0.86-0.97) and specificity of 92% (95% CI, 0.86-0.96), with a PPV of 90% (95% CI, 0.82-0.94), NPV of 94% (95% CI, 0.89-0.98), accuracy of 92% (95% CI, 0.88-0.95), and AUC of 0.92. The combination demonstrated significantly higher sensitivity (p=0.004) and accuracy (p=0.02) compared to BiliSeqV2/V3 alone at second repeat testing.

For collective performance across all testing episodes, BiliSeqV2/V3 demonstrated a sensitivity of 85% (95% CI, 0.77-0.91) and specificity of 93% (95% CI, 0.87-0.96), with a PPV of 90% (95% CI, 0.82-0.95), NPV of 89% (95% CI, 0.83-0.93), accuracy of 89% (95% CI, 0.85-0.93), and AUC of 0.89 (**Supplementary Figure 21**). Pathologic evaluation alone demonstrated a collective sensitivity of 33% (95% CI, 0.25-0.43) and specificity of 98% (95% CI, 0.94-1.00), with a PPV of 92% (95% CI, 0.80-0.98), NPV of 66% (95% CI, 0.60-0.73), accuracy of 70% (95% CI, 0.64-0.76), and AUC of 0.66. Collectively, BiliSeqV2/V3 demonstrated significantly higher

sensitivity compared to pathologic evaluation alone (p<0.001), CA19-9≥44 U/mL (p<0.001), and CA19-9>129 U/mL (p<0.001). BiliSeqV2/V3 demonstrated significantly higher specificity compared to CA19-9≥44 U/mL (p<0.001). BiliSeqV2/V3 also demonstrated significantly higher accuracy compared to pathologic evaluation alone (p<0.001), CA19-9≥44 U/mL (p<0.001), and CA19-9>129 U/mL (p<0.001). The collective combination of pathologic evaluation and BiliSeqV2/V3 demonstrated a sensitivity of 93% (95% CI, 0.86-0.97) and specificity of 92% (95% CI, 0.86-0.96), with a PPV of 90% (95% CI, 0.82-0.94), NPV of 94% (95% CI, 0.89-0.98), accuracy of 92% (95% CI, 0.88-0.95), and AUC of 0.92. The collective combination demonstrated significantly higher sensitivity (p=0.004) and accuracy (p=0.02) compared to BiliSeqV2/V3 alone, with significantly lower specificity compared to pathologic evaluation alone (p=0.01).

Classification of Dysplasia as a Benign Bile Duct Stricture

In the primary analysis, dysplastic lesions (low-grade and high-grade dysplasia) were classified as neoplastic outcomes. To evaluate the diagnostic performance for detecting malignancy specifically, an analysis was performed in which dysplastic lesions were reclassified as benign outcomes, resulting in a comparison of benign versus malignant diagnoses (**Supplementary Figure 22**). In this analysis, BiliSeqV2/V3 demonstrated a sensitivity of 82% (95% CI, 0.79-0.84) and specificity of 96% (95% CI, 0.95-0.97) for the detection of malignancy in 1,979 patients with follow-up, with a PPV of 96% (95% CI, 0.95-0.97), NPV of 81% (95% CI, 0.79-0.83), and accuracy of 88% (95% CI, 0.87-0.90). The AUC was 0.89 for BiliSeqV2/V3. Pathologic evaluation alone demonstrated a sensitivity of 44% (95% CI, 0.41-0.47) and specificity of 99% (95% CI, 0.98-0.99), with a PPV of 97% (95% CI, 0.96-0.99), NPV of 59% (95% CI, 0.56-0.62), accuracy of 69% (95% CI, 0.67-0.71), and AUC of 0.71. Among the 918 patients with available serum CA19-9 levels, CA19-9 at a threshold of ≥44 U/mL demonstrated a sensitivity of 70% (95% CI, 0.66-0.73) and specificity of 55% (95% CI, 0.49-0.61), with a PPV of 78% (95% CI, 0.75-0.82), NPV of 44% (95% CI, 0.39-0.49), accuracy of 65% (95% CI, 0.62-0.69), and AUC of 0.63. CA19-

9 at a threshold of >129 U/mL demonstrated a sensitivity of 54% (95% CI, 0.50-0.58) and specificity of 79% (95% CI, 0.73-0.83), with a PPV of 86% (95% CI, 0.82-0.89), NPV of 43% (95% CI, 0.38-0.47), accuracy of 62% (95% CI, 0.58-0.65), and AUC of 0.66.

BiliSeqV2/V3 had significantly higher sensitivity compared to pathologic evaluation alone (p<0.001), CA19-9≥44 U/mL (p<0.001), and CA19-9>129 U/mL (p<0.001). BiliSeqV2/V3 demonstrated significantly higher specificity compared to pathologic evaluation alone (p=0.002), CA19-9≥44 U/mL (p<0.001), and CA19-9>129 U/mL (p<0.001). BiliSeqV2/V3 also demonstrated significantly higher accuracy compared to pathologic evaluation alone (p<0.001), CA19-9≥44 U/mL (p<0.001), and CA19-9>129 U/mL (p<0.001).

The combination of pathologic evaluation and BiliSeqV2/V3 demonstrated a sensitivity of 88% (95% CI, 0.86-0.90) and specificity of 95% (95% CI, 0.93-0.96), with a PPV of 96% (95% CI, 0.94-0.97), NPV of 86% (95% CI, 0.84-0.88), accuracy of 91% (95% CI, 0.90-0.92), and AUC of 0.91. The combination demonstrated significantly higher sensitivity compared to BiliSeqV2/V3 alone (p<0.001), pathologic evaluation alone (p<0.001), CA19-9≥44 U/mL (p<0.001), and CA19-9>129 U/mL (p<0.001). Specificity was significantly lower for the combination compared to pathologic evaluation alone (p<0.001) and BiliSeqV2/V3 alone (p=0.002). The combination demonstrated significantly higher accuracy compared to BiliSeqV2/V3 alone (p<0.001), pathologic evaluation alone (p<0.001), CA19-9≥44 U/mL (p<0.001), and CA19-9>129 U/mL (p<0.001). When classifying dysplasia as benign rather than neoplastic, specificity decreased from 98% to 96% for BiliSeqV2/V3, reflecting the detection of molecular alterations in some dysplastic lesions. All other performance metrics remained essentially unchanged.

Exclusion of Pancreatic Ductal Adenocarcinoma and Ampullary Adenocarcinoma

Pancreatic ductal adenocarcinoma (PDAC) and ampullary adenocarcinoma are frequently diagnosed using endoscopic ultrasound-guided fine needle aspiration (EUS-FNA) rather than ERCP-based biliary sampling. To evaluate the diagnostic performance of BiliSeqV2/V3 in a cohort

where ERCP sampling serves as the primary diagnostic modality, a sensitivity analysis was performed excluding patients with final diagnoses of PDAC and ampullary adenocarcinoma (**Supplementary Figure 23**). In this analysis of 1,599 patients, BiliSeqV2/V3 demonstrated a sensitivity of 82% (95% CI, 0.79-0.85) and specificity of 98% (95% CI, 0.97-0.99) for the detection of neoplasia, with a PPV of 97% (95% CI, 0.96-0.98), NPV of 87% (95% CI, 0.84-0.89), and accuracy of 91% (95% CI, 0.89-0.92). The AUC was 0.90 for BiliSeqV2/V3. Pathologic evaluation alone demonstrated a sensitivity of 41% (95% CI, 0.37-0.44) and specificity of 99% (95% CI, 0.98-0.99) in 1,594 patients, with a PPV of 97% (95% CI, 0.94-0.98), NPV of 66% (95% CI, 0.64-0.69), accuracy of 72% (95% CI, 0.70-0.74), and AUC of 0.70. Among the 655 patients with available serum CA19-9 levels, CA19-9 at a threshold of ≥44 U/mL demonstrated a sensitivity of 67% (95% CI, 0.62-0.72) and specificity of 54% (95% CI, 0.48-0.60), with a PPV of 68% (95% CI, 0.63-0.73), NPV of 53% (95% CI, 0.47-0.59), accuracy of 62% (95% CI, 0.58-0.66), and AUC of 0.61. CA19-9 at a threshold of >129 U/mL demonstrated a sensitivity of 52% (95% CI, 0.47-0.57) and specificity of 78% (95% CI, 0.72-0.83), with a PPV of 78% (95% CI, 0.72-0.82), NPV of 53% (95% CI, 0.48-0.58), accuracy of 63% (95% CI, 0.59-0.66), and AUC of 0.65.

BiliSeqV2/V3 had significantly higher sensitivity compared to pathologic evaluation alone (p<0.001), CA19-9≥44 U/mL (p<0.001), and CA19-9>129 U/mL (p<0.001). BiliSeqV2/V3 demonstrated significantly higher specificity compared to CA19-9≥44 U/mL (p<0.001) and CA19-9>129 U/mL (p<0.001). BiliSeqV2/V3 also demonstrated significantly higher accuracy compared to pathologic evaluation alone (p<0.001), CA19-9≥44 U/mL (p<0.001), and CA19-9>129 U/mL (p<0.001).

The combination of pathologic evaluation and BiliSeqV2/V3 demonstrated a sensitivity of 88% (95% CI, 0.85-0.90) and specificity of 97% (95% CI, 0.96-0.98), with a PPV of 96% (95% CI, 0.94-0.97), NPV of 91% (95% CI, 0.89-0.92), accuracy of 93% (95% CI, 0.91-0.94), and AUC of 0.92. The combination demonstrated significantly higher sensitivity compared to BiliSeqV2/V3 alone (p<0.001), pathologic evaluation alone (p<0.001), CA19-9≥44 U/mL (p<0.001), and CA19-

9>129 U/mL ($p<0.001$). Specificity was significantly lower for the combination compared to pathologic evaluation alone ($p<0.001$) and BiliSeqV2/V3 alone ($p=0.002$). The combination demonstrated significantly higher accuracy compared to BiliSeqV2/V3 alone ($p<0.001$), pathologic evaluation alone ($p<0.001$), CA19-9≥44 U/mL ($p<0.001$), and CA19-9>129 U/mL ($p<0.001$). After excluding PDAC and ampullary adenocarcinoma, NPV improved from 81% to 87% and accuracy improved from 89% to 91% for BiliSeqV2/V3, reflecting the increased proportion of benign outcomes and the consistent detection of primary biliary tract neoplasms.

Supplementary Figure Legend

Supplementary Figure 1. A flow diagram showing the study design that includes details of individual patient cohorts, BiliSeq version 2 (BiliSeqV2), BiliSeq version 3 (BiliSeqV3), statistical analysis, repeat testing, and identification of therapeutically relevant genomic alterations (GAs).

Supplementary Figure 2. Variant allele frequencies (VAF) of the most prevalent genomic alterations detected by BiliSeqV2 and BiliSeqV3. Gray and red circles correspond to individual patients with neoplastic and non-neoplastic bile duct strictures, respectively. The dotted green line represents a VAF of 1.5%.

Supplementary Figure 3. Diagnostic performance of CA19-9, cytopathology, and BiliSeqV2 testing for detecting neoplasia in biliary strictures. (A-E) ROC curves with AUC values for each diagnostic test. Red circles indicate optimal operating points. (F) Forest plot comparing sensitivity, specificity, NPV, PPV, and accuracy with 95% confidence intervals. (G-K) McNemar's test p-value matrices for pairwise comparisons of diagnostic performance. Dark red indicates $p < 0.001$, lighter shades $p < 0.01$ and $p < 0.05$, and gray indicates non-significant differences ($p \geq 0.05$).

Supplementary Figure 4. Diagnostic performance of CA19-9, cytopathology, and BiliSeqV3 testing for detecting neoplasia in biliary strictures. (A-E) ROC curves with AUC values for each diagnostic test. Red circles indicate optimal operating points. (F) Forest plot comparing sensitivity, specificity, NPV, PPV, and accuracy with 95% confidence intervals. (G-K) McNemar's test p-value matrices for pairwise comparisons of diagnostic performance. Dark red indicates $p < 0.001$, lighter shades $p < 0.01$ and $p < 0.05$, and gray indicates non-significant differences ($p \geq 0.05$).

Supplementary Figure 5. Diagnostic performance of CA19-9, cytopathology, and BiliSeqV2/V3 testing for detecting neoplasia in biliary strictures. (A-E) ROC curves with AUC values for each

diagnostic test. Red circles indicate optimal operating points. (F) Forest plot comparing sensitivity, specificity, NPV, PPV, and accuracy with 95% confidence intervals. (G-K) McNemar's test p-value matrices for pairwise comparisons of diagnostic performance. Dark red indicates $p < 0.001$, lighter shades $p < 0.01$ and $p < 0.05$, and gray indicates non-significant differences ($p \geq 0.05$).

Supplementary Figure 6. Diagnostic performance of CA19-9, cytopathology, and BiliSeqV2/V3 testing for detecting neoplasia from bile duct brushing specimens. (A-E) ROC curves with AUC values for each diagnostic test. Red circles indicate optimal operating points. (F) Forest plot comparing sensitivity, specificity, NPV, PPV, and accuracy with 95% confidence intervals. (G-K) McNemar's test p-value matrices for pairwise comparisons of diagnostic performance. Dark red indicates $p < 0.001$, lighter shades $p < 0.01$ and $p < 0.05$, and gray indicates non-significant differences ($p \geq 0.05$).

Supplementary Figure 7. Diagnostic performance of CA19-9, cytopathology, and BiliSeqV2/V3 testing for detecting neoplasia from bile duct biopsy specimens. (A-E) ROC curves with AUC values for each diagnostic test. Red circles indicate optimal operating points. (F) Forest plot comparing sensitivity, specificity, NPV, PPV, and accuracy with 95% confidence intervals. (G-K) McNemar's test p-value matrices for pairwise comparisons of diagnostic performance. Dark red indicates $p < 0.001$, lighter shades $p < 0.01$ and $p < 0.05$, and gray indicates non-significant differences ($p \geq 0.05$).

Supplementary Figure 8. Diagnostic performance of CA19-9, cytopathology, and BiliSeqV2/V3 testing for detecting neoplasia in intrahepatic/hilar biliary strictures. (A-E) ROC curves with AUC values for each diagnostic test. Red circles indicate optimal operating points. (F) Forest plot comparing sensitivity, specificity, NPV, PPV, and accuracy with 95% confidence intervals. (G-K) McNemar's test p-value matrices for pairwise comparisons of diagnostic performance. Dark red

indicates $p < 0.001$, lighter shades $p < 0.01$ and $p < 0.05$, and gray indicates non-significant differences ($p \geq 0.05$).

Supplementary Figure 9. Diagnostic performance of CA19-9, cytopathology, and BiliSeqV2/V3 testing for detecting neoplasia in middle bile duct strictures. (A-E) ROC curves with AUC values for each diagnostic test. Red circles indicate optimal operating points. (F) Forest plot comparing sensitivity, specificity, NPV, PPV, and accuracy with 95% confidence intervals. (G-K) McNemar's test p-value matrices for pairwise comparisons of diagnostic performance. Dark red indicates $p < 0.001$, lighter shades $p < 0.01$ and $p < 0.05$, and gray indicates non-significant differences ($p \geq 0.05$).

Supplementary Figure 10. Diagnostic performance of CA19-9, cytopathology, and BiliSeqV2/V3 testing for detecting neoplasia in distal bile duct strictures. (A-E) ROC curves with AUC values for each diagnostic test. Red circles indicate optimal operating points. (F) Forest plot comparing sensitivity, specificity, NPV, PPV, and accuracy with 95% confidence intervals. (G-K) McNemar's test p-value matrices for pairwise comparisons of diagnostic performance. Dark red indicates $p < 0.001$, lighter shades $p < 0.01$ and $p < 0.05$, and gray indicates non-significant differences ($p \geq 0.05$).

Supplementary Figure 11. Diagnostic performance of CA19-9, cytopathology, and BiliSeqV2/V3 testing for detecting neoplasia in indeterminate bile duct strictures. (A-E) ROC curves with AUC values for each diagnostic test. Red circles indicate optimal operating points. (F) Forest plot comparing sensitivity, specificity, NPV, PPV, and accuracy with 95% confidence intervals. (G-K) McNemar's test p-value matrices for pairwise comparisons of diagnostic performance. Dark red indicates $p < 0.001$, lighter shades $p < 0.01$ and $p < 0.05$, and gray indicates non-significant differences ($p \geq 0.05$).

Supplementary Figure 12. Diagnostic performance of CA19-9, cytopathology, and BiliSeqV2/V3 testing for detecting neoplasia in bile duct strictures from primary sclerosing cholangitis (PSC) patients. (A-E) ROC curves with AUC values for each diagnostic test. Red circles indicate optimal operating points. (F) Forest plot comparing sensitivity, specificity, NPV, PPV, and accuracy with 95% confidence intervals. (G-K) McNemar's test p-value matrices for pairwise comparisons of diagnostic performance. Dark red indicates $p < 0.001$, lighter shades $p < 0.01$ and $p < 0.05$, and gray indicates non-significant differences ($p \geq 0.05$).

Supplementary Figure 13. Diagnostic performance of CA19-9, cytopathology, and BiliSeqV3 testing for detecting neoplasia in bile duct strictures from patients with germline alterations. (A-E) ROC curves with AUC values for each diagnostic test. Red circles indicate optimal operating points. (F) Forest plot comparing sensitivity, specificity, NPV, PPV, and accuracy with 95% confidence intervals. (G-K) McNemar's test p-value matrices for pairwise comparisons of diagnostic performance. Dark red indicates $p < 0.001$, lighter shades $p < 0.01$ and $p < 0.05$, and gray indicates non-significant differences ($p \geq 0.05$).

Supplementary Figure 14. Diagnostic performance of CA19-9, cytopathology, and BiliSeqV2/V3 testing for detecting neoplasia in bile duct strictures from Caucasian patients. (A-E) ROC curves with AUC values for each diagnostic test. Red circles indicate optimal operating points. (F) Forest plot comparing sensitivity, specificity, NPV, PPV, and accuracy with 95% confidence intervals. (G-K) McNemar's test p-value matrices for pairwise comparisons of diagnostic performance. Dark red indicates $p < 0.001$, lighter shades $p < 0.01$ and $p < 0.05$, and gray indicates non-significant differences ($p \geq 0.05$).

Supplementary Figure 15. Diagnostic performance of CA19-9, cytopathology, and BiliSeqV2/V3 testing for detecting neoplasia in bile duct strictures from Black patients. (A-E) ROC curves with AUC values for each diagnostic test. Red circles indicate optimal operating points. (F) Forest plot comparing sensitivity, specificity, NPV, PPV, and accuracy with 95% confidence intervals. (G-K) McNemar's test p-value matrices for pairwise comparisons of diagnostic performance. Dark red indicates $p < 0.001$, lighter shades $p < 0.01$ and $p < 0.05$, and gray indicates non-significant differences ($p \geq 0.05$).

Supplementary Figure 16. Diagnostic performance of CA19-9, cytopathology, and BiliSeqV2/V3 testing for detecting neoplasia in bile duct strictures from Hispanic patients. (A-E) ROC curves with AUC values for each diagnostic test. Red circles indicate optimal operating points. (F) Forest plot comparing sensitivity, specificity, NPV, PPV, and accuracy with 95% confidence intervals. (G-K) McNemar's test p-value matrices for pairwise comparisons of diagnostic performance. Dark red indicates $p < 0.001$, lighter shades $p < 0.01$ and $p < 0.05$, and gray indicates non-significant differences ($p \geq 0.05$).

Supplementary Figure 17. Diagnostic performance of CA19-9, cytopathology, and BiliSeqV2/V3 testing for detecting neoplasia in bile duct strictures from Asian patients. (A-E) ROC curves with AUC values for each diagnostic test. Red circles indicate optimal operating points. (F) Forest plot comparing sensitivity, specificity, NPV, PPV, and accuracy with 95% confidence intervals. (G-K) McNemar's test p-value matrices for pairwise comparisons of diagnostic performance. Dark red indicates $p < 0.001$, lighter shades $p < 0.01$ and $p < 0.05$, and gray indicates non-significant differences ($p \geq 0.05$).

Supplementary Figure 18. Diagnostic performance of CA19-9, cytopathology, and BiliSeqV2/V3 testing for detecting neoplasia in bile duct strictures from repeat testing patients (initial ERCP).

(A-E) ROC curves with AUC values for each diagnostic test. Red circles indicate optimal operating points. (F) Forest plot comparing sensitivity, specificity, NPV, PPV, and accuracy with 95% confidence intervals. (G-K) McNemar's test p-value matrices for pairwise comparisons of diagnostic performance. Dark red indicates $p < 0.001$, lighter shades $p < 0.01$ and $p < 0.05$, and gray indicates non-significant differences ($p \geq 0.05$).

Supplementary Figure 19. Diagnostic performance of CA19-9, cytopathology, and BiliSeqV2/V3 testing for detecting neoplasia in bile duct strictures from repeat testing patients (1st repeat/2nd ERCP). (A-E) ROC curves with AUC values for each diagnostic test. Red circles indicate optimal operating points. (F) Forest plot comparing sensitivity, specificity, NPV, PPV, and accuracy with 95% confidence intervals. (G-K) McNemar's test p-value matrices for pairwise comparisons of diagnostic performance. Dark red indicates $p < 0.001$, lighter shades $p < 0.01$ and $p < 0.05$, and gray indicates non-significant differences ($p \geq 0.05$).

Supplementary Figure 20. Diagnostic performance of CA19-9, cytopathology, and BiliSeqV2/V3 testing for detecting neoplasia in bile duct strictures from repeat testing patients (2nd repeat/3rd ERCP). (A-E) ROC curves with AUC values for each diagnostic test. Red circles indicate optimal operating points. (F) Forest plot comparing sensitivity, specificity, NPV, PPV, and accuracy with 95% confidence intervals. (G-K) McNemar's test p-value matrices for pairwise comparisons of diagnostic performance. Dark red indicates $p < 0.001$, lighter shades $p < 0.01$ and $p < 0.05$, and gray indicates non-significant differences ($p \geq 0.05$).

Supplementary Figure 21. Diagnostic performance of CA19-9, cytopathology, and BiliSeqV2/V3 testing for detecting neoplasia in bile duct strictures from repeat testing patients (collective testing). (A-E) ROC curves with AUC values for each diagnostic test. Red circles indicate optimal operating points. (F) Forest plot comparing sensitivity, specificity, NPV, PPV, and accuracy with

95% confidence intervals. (G-K) McNemar's test p-value matrices for pairwise comparisons of diagnostic performance. Dark red indicates $p < 0.001$, lighter shades $p < 0.01$ and $p < 0.05$, and gray indicates non-significant differences ($p \geq 0.05$).

Supplementary Figure 22. Diagnostic performance of CA19-9, cytopathology, and BiliSeqV2/V3 testing for detecting malignancy in bile duct strictures with dysplasia classified as benign. (A-E) ROC curves with AUC values for each diagnostic test. Red circles indicate optimal operating points. (F) Forest plot comparing sensitivity, specificity, NPV, PPV, and accuracy with 95% confidence intervals. (G-K) McNemar's test p-value matrices for pairwise comparisons of diagnostic performance. Dark red indicates $p < 0.001$, lighter shades $p < 0.01$ and $p < 0.05$, and gray indicates non-significant differences ($p \geq 0.05$).

Supplementary Figure 23. Diagnostic performance of CA19-9, cytopathology, and BiliSeqV2/V3 testing for detecting neoplasia in bile duct strictures, which excluded pancreatic ductal adenocarcinoma and ampullary adenocarcinoma. (A-E) ROC curves with AUC values for each diagnostic test. Red circles indicate optimal operating points. (F) Forest plot comparing sensitivity, specificity, NPV, PPV, and accuracy with 95% confidence intervals. (G-K) McNemar's test p-value matrices for pairwise comparisons of diagnostic performance. Dark red indicates $p < 0.001$, lighter shades $p < 0.01$ and $p < 0.05$, and gray indicates non-significant differences ($p \geq 0.05$).

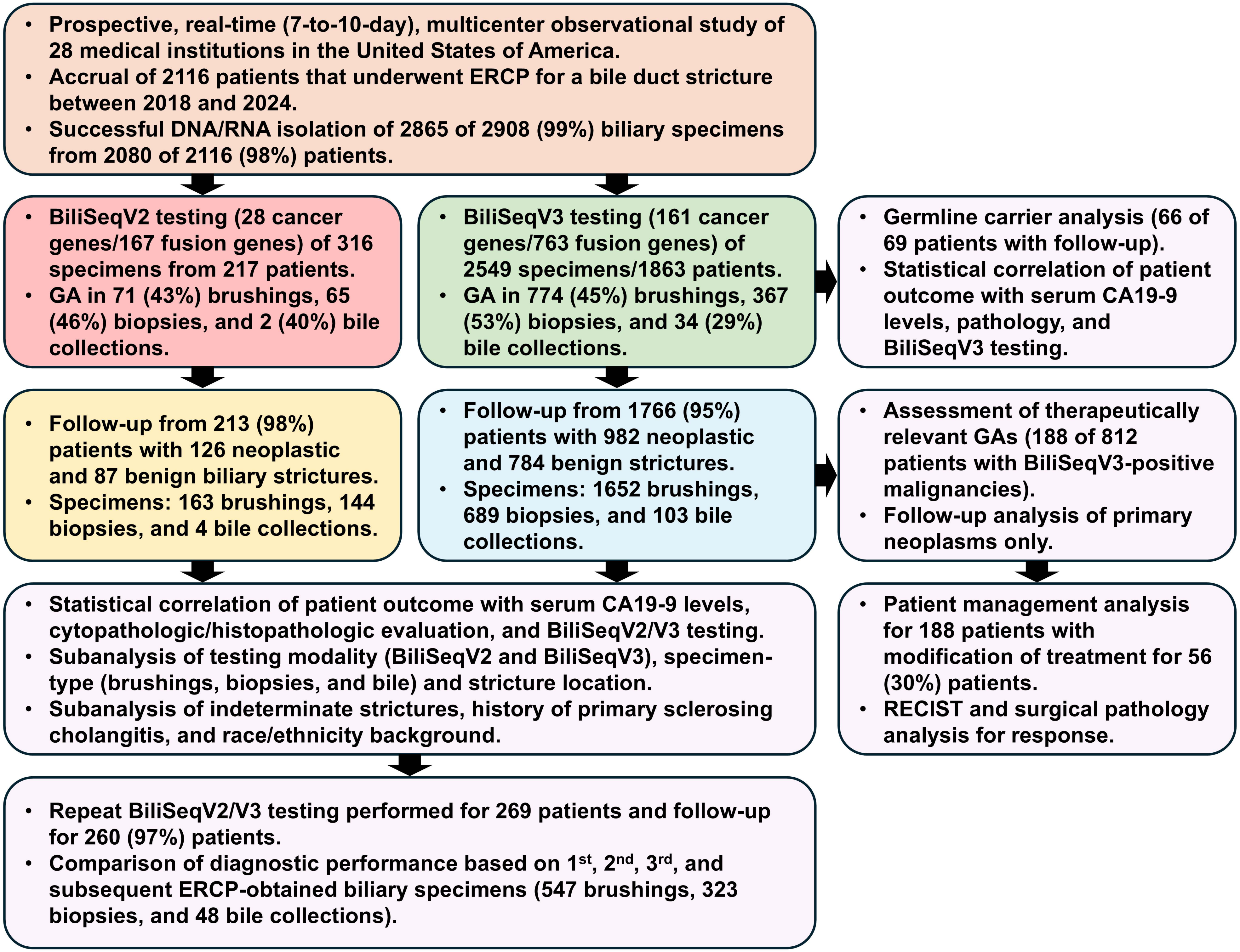
• Prospective, real-time (7-to-10-day), multicenter observational study of 28 medical institutions in the United States of America.
• Accrual of 2116 patients that underwent ERCP for a bile duct stricture between 2018 and 2024.
• Successful DNA/RNA isolation of 2865 of 2908 (99%) biliary specimens from 2080 of 2116 (98%) patients.
• BiliSeqV2 testing (28 cancer genes/167 fusion genes) of 316 specimens from 217 patients.
• GA in 71 (43%) brushings, 65 (46%) biopsies, and 2 (40%) bile collections.
• BiliSeqV3 testing (161 cancer genes/763 fusion genes) of 2549 specimens/1863 patients.
• GA in 774 (45%) brushings, 367 (53%) biopsies, and 34 (29%) bile collections.
• Germline carrier analysis (66 of 69 patients with follow-up).
• Statistical correlation of patient outcome with serum CA19-9 levels, pathology, and BiliSeqV3 testing.
• Follow-up from 213 (98%) patients with 126 neoplastic and 87 benign biliary strictures.
• Specimens: 163 brushings, 144 biopsies, and 4 bile collections.
• Follow-up from 1766 (95%) patients with 982 neoplastic and 784 benign strictures.
• Specimens: 1652 brushings, 689 biopsies, and 103 bile collections.
• Assessment of therapeutically relevant GAs (188 of 812 patients with BiliSeqV3-positive malignancies).
• Follow-up analysis of primary neoplasms only.
• Statistical correlation of patient outcome with serum CA19-9 levels, cytopathologic/histopathologic evaluation, and BiliSeqV2/V3 testing.
• Subanalysis of testing modality (BiliSeqV2 and BiliSeqV3), specimen-type (brushings, biopsies, and bile) and stricture location.
• Subanalysis of indeterminate strictures, history of primary sclerosing cholangitis, and race/ethnicity background.
• Patient management analysis for 188 patients with modification of treatment for 56 (30%) patients.
• RECIST and surgical pathology analysis for response.
• Repeat BiliSeqV2/V3 testing performed for 269 patients and follow-up for 260 (97%) patients.
• Comparison of diagnostic performance based on 1st, 2nd, 3rd, and subsequent ERCP-obtained biliary specimens (547 brushings, 323 biopsies, and 48 bile collections).

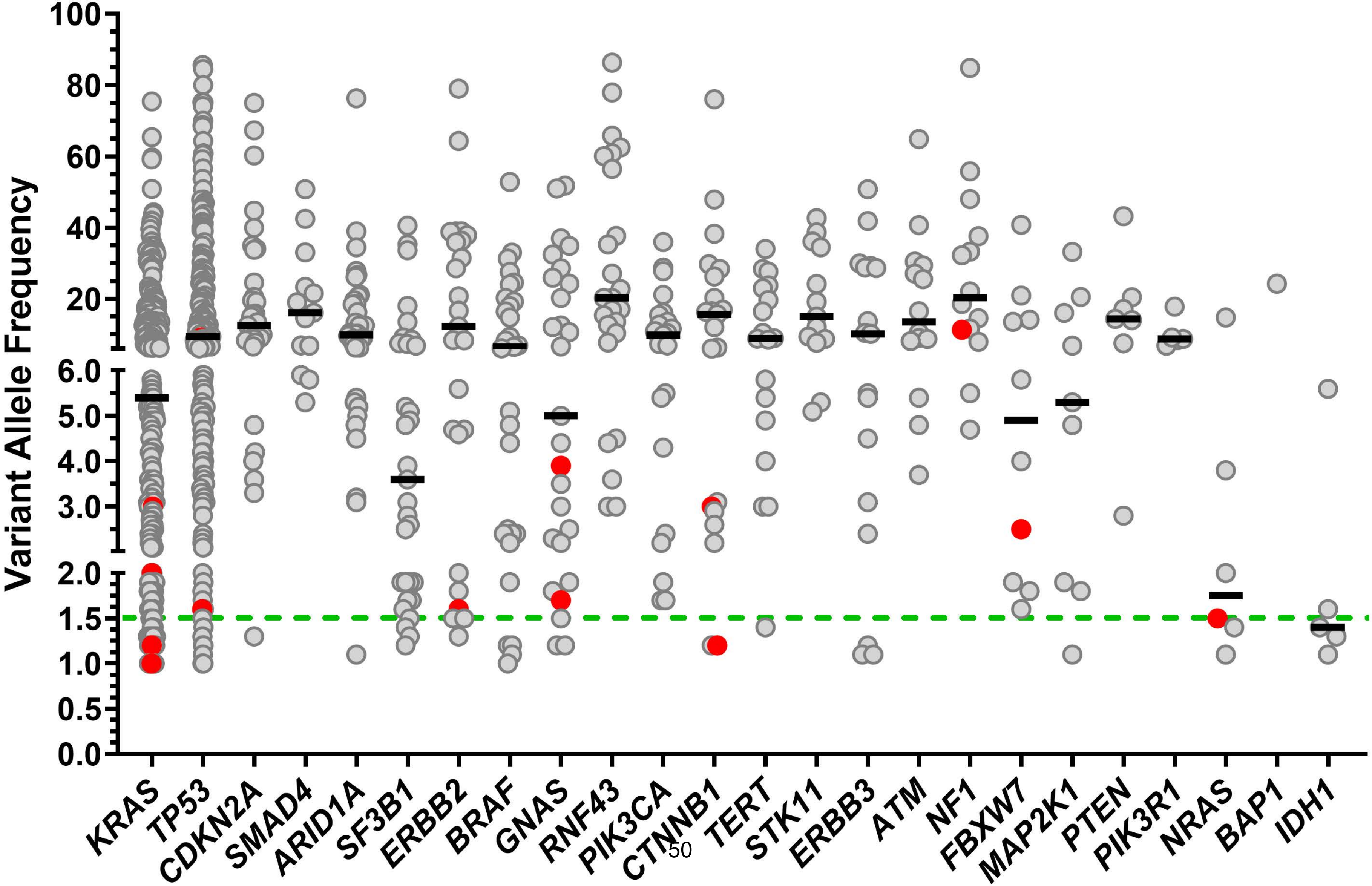

Variant Allele Frequency
100
80
60
40
20
6.0
5.0
4.0
3.0
2.0
1.5
1.0
0.5
0.0
KRAS
TP53
CDKN2A
SMAD4
ARID1A
SF3B1
ERBB2
BRAF
GNAS
RNF43
PIK3CA
CTNNB1
TERT
STK11
ERBB3
ATM
NF1
FBXW7
MAP2K1
PTEN
PIK3R1
NRAS
BAP1
IDH1

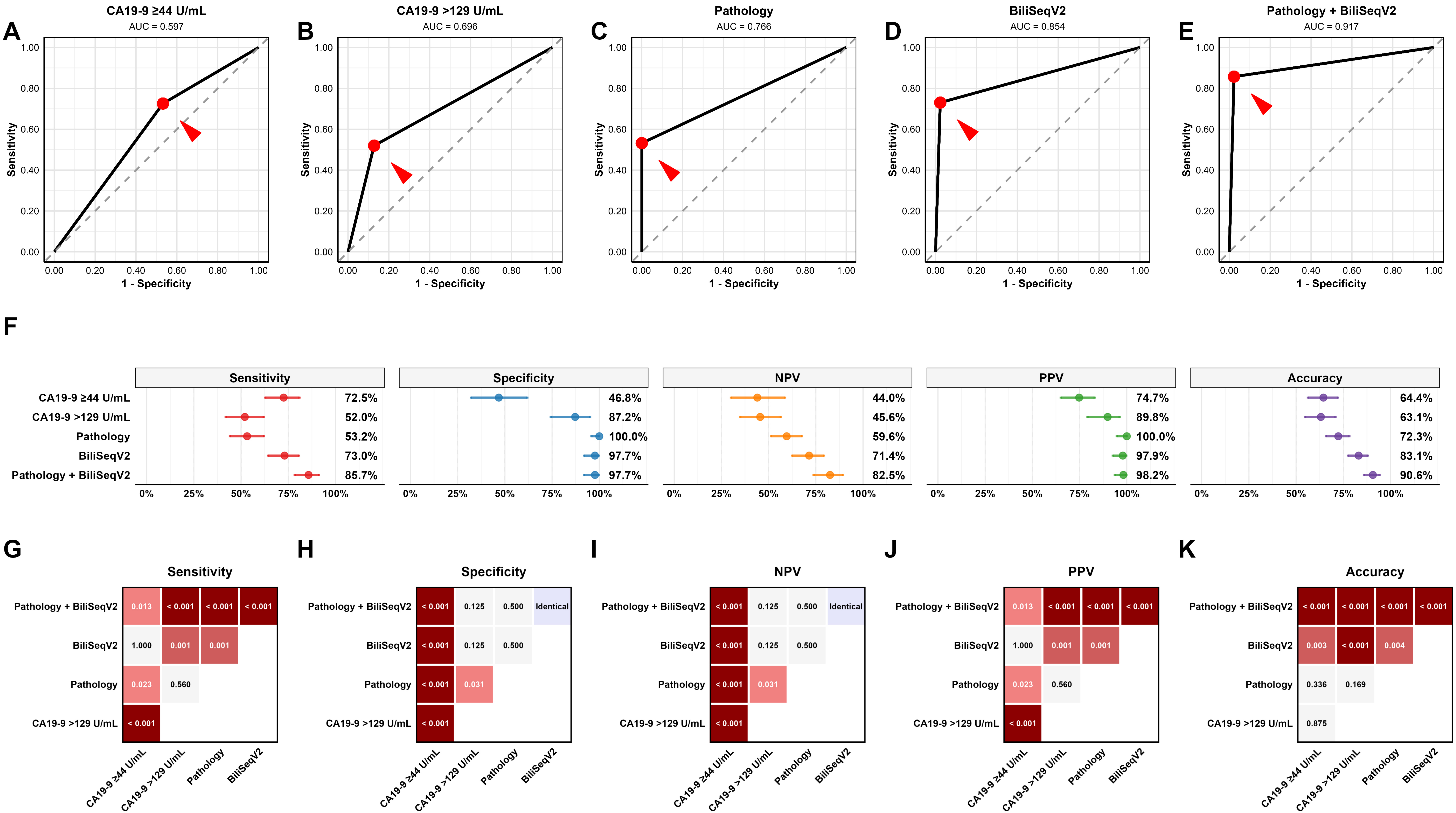

A
CA19-9 ≥44 U/mL
AUC = 0.597
B
CA19-9 >129 U/mL
AUC = 0.696
C
Pathology
AUC = 0.766
D
BiliSeqV2
AUC = 0.854
E
Pathology + BiliSeqV2
AUC = 0.917
Sensitivity
1 - Specificity
0.00 0.20 0.40 0.60 0.80 1.00
F
Sensitivity
Specificity
NPV
PPV
Accuracy
CA19-9 ≥44 U/mL
CA19-9 >129 U/mL
Pathology
BiliSeqV2
Pathology + BiliSeqV2
72.5% 52.0% 53.2% 73.0% 85.7%
46.8% 87.2% 100.0% 97.7% 97.7%
44.0% 45.6% 59.6% 71.4% 82.5%
74.7% 89.8% 100.0% 97.9% 98.2%
64.4% 63.1% 72.3% 83.1% 90.6%
0% 25% 50% 75% 100%
G
Sensitivity
Pathology + BiliSeqV2: 0.013, < 0.001, < 0.001, < 0.001
BiliSeqV2: 1.000, 0.001, 0.001
Pathology: 0.023, 0.560
CA19-9 >129 U/mL: < 0.001
H
Specificity
Pathology + BiliSeqV2: < 0.001, 0.125, 0.500, Identical
BiliSeqV2: < 0.001, 0.125, 0.500
Pathology: < 0.001, 0.031
CA19-9 >129 U/mL: < 0.001
I
NPV
Pathology + BiliSeqV2: < 0.001, 0.125, 0.500, Identical
BiliSeqV2: < 0.001, 0.125, 0.500
Pathology: < 0.001, 0.031
CA19-9 >129 U/mL: < 0.001
J
PPV
Pathology + BiliSeqV2: 0.013, < 0.001, < 0.001, < 0.001
BiliSeqV2: 1.000, 0.001, 0.001
Pathology: 0.023, 0.560
CA19-9 >129 U/mL: < 0.001
K
Accuracy
Pathology + BiliSeqV2: < 0.001, < 0.001, < 0.001, < 0.001
BiliSeqV2: 0.003, < 0.001, 0.004
Pathology: 0.336, 0.169
CA19-9 >129 U/mL: 0.875
CA19-9 ≥44 U/mL
CA19-9 >129 U/mL
Pathology
BiliSeqV2

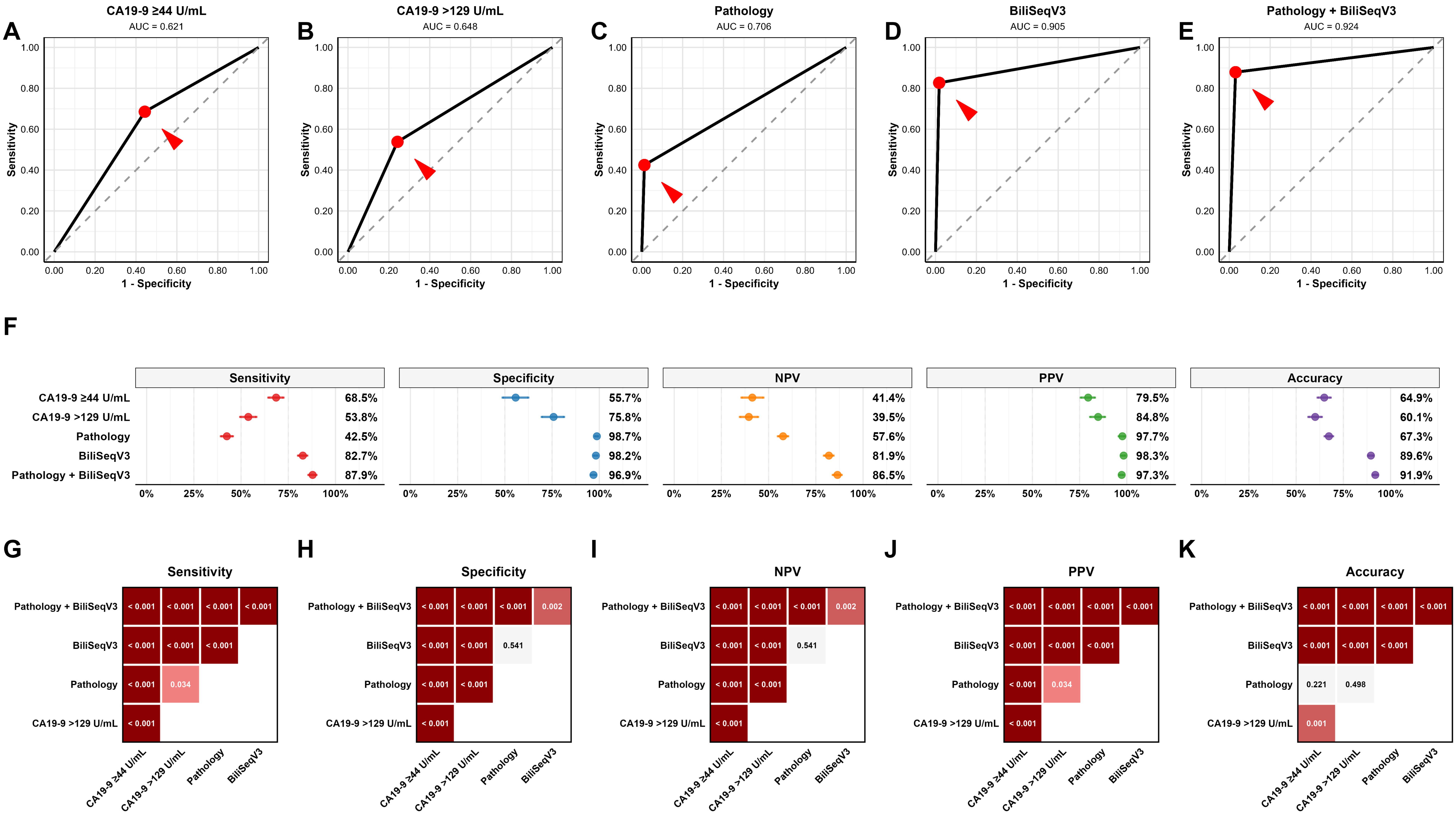
A
CA19-9 ≥44 U/mL
AUC = 0.621
B
CA19-9 >129 U/mL
AUC = 0.648
C
Pathology
AUC = 0.706
D
BiliSeqV3
AUC = 0.905
E
Pathology + BiliSeqV3
AUC = 0.924
Sensitivity
1 - Specificity
F
Sensitivity
Specificity
NPV
PPV
Accuracy
CA19-9 ≥44 U/mL
CA19-9 >129 U/mL
Pathology
BiliSeqV3
Pathology + BiliSeqV3
68.5%
53.8%
42.5%
82.7%
87.9%
55.7%
75.8%
98.7%
98.2%
96.9%
41.4%
39.5%
57.6%
81.9%
86.5%
79.5%
84.8%
97.7%
98.3%
97.3%
64.9%
60.1%
67.3%
89.6%
91.9%
G
Sensitivity
H
Specificity
I
NPV
J
PPV
K
Accuracy
0.002
0.541
0.034
0.221
0.498
0.001

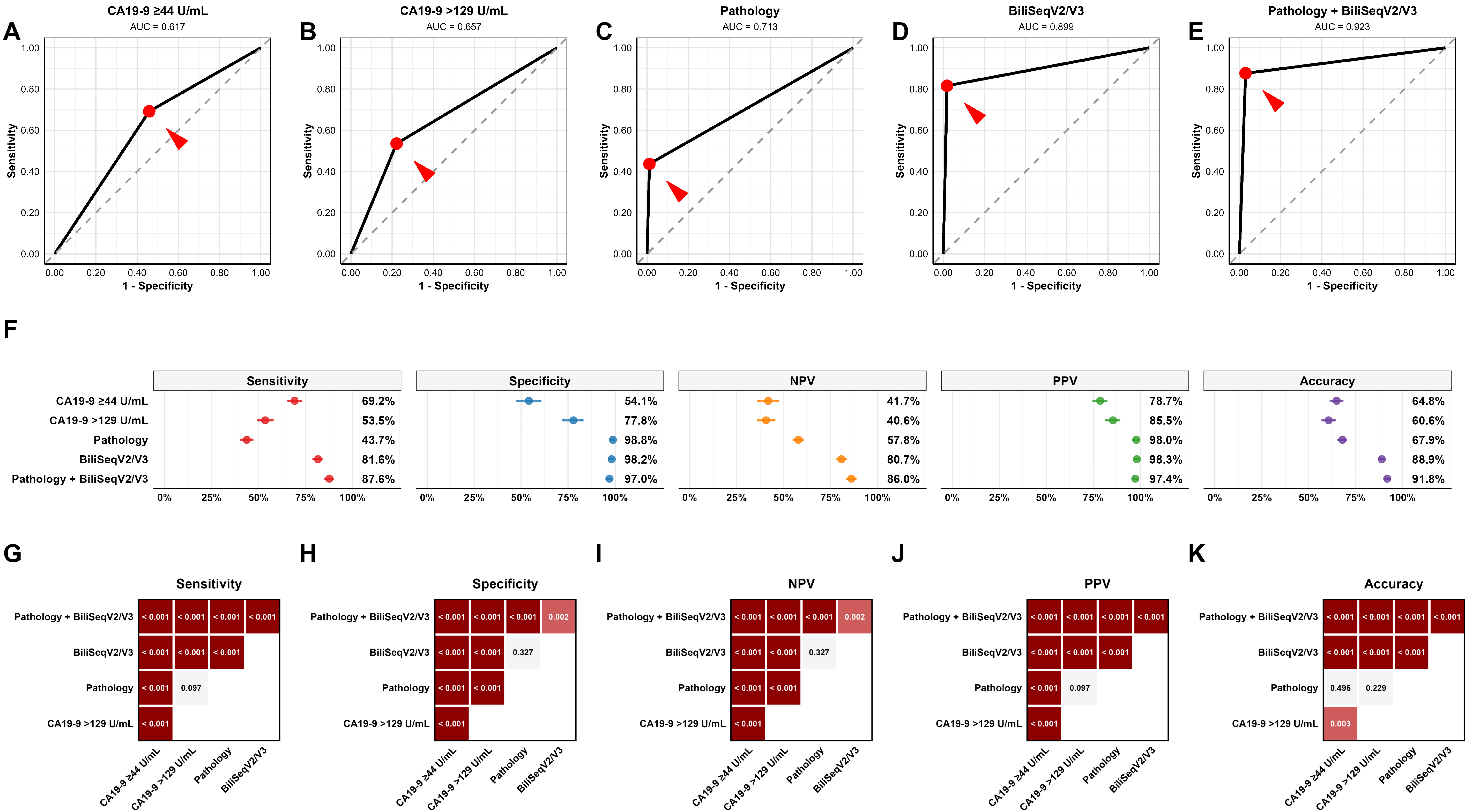

A
CA19-9 ≥44 U/mL
AUC = 0.617
B
CA19-9 >129 U/mL
AUC = 0.657
C
Pathology
AUC = 0.713
D
BiliSeqV2/V3
AUC = 0.899
E
Pathology + BiliSeqV2/V3
AUC = 0.923
Sensitivity
1 - Specificity
F
Sensitivity
Specificity
NPV
PPV
Accuracy
CA19-9 ≥44 U/mL 69.2% 54.1% 41.7% 78.7% 64.8%
CA19-9 >129 U/mL 53.5% 77.8% 40.6% 85.5% 60.6%
Pathology 43.7% 98.8% 57.8% 98.0% 67.9%
BiliSeqV2/V3 81.6% 98.2% 80.7% 98.3% 88.9%
Pathology + BiliSeqV2/V3 87.6% 97.0% 86.0% 97.4% 91.8%
G
H
I
J
K
Sensitivity
Specificity
NPV
PPV
Accuracy
Pathology + BiliSeqV2/V3
BiliSeqV2/V3
Pathology
CA19-9 >129 U/mL
CA19-9 ≥44 U/mL
< 0.001
0.097
0.002
0.327
0.496
0.229
0.003

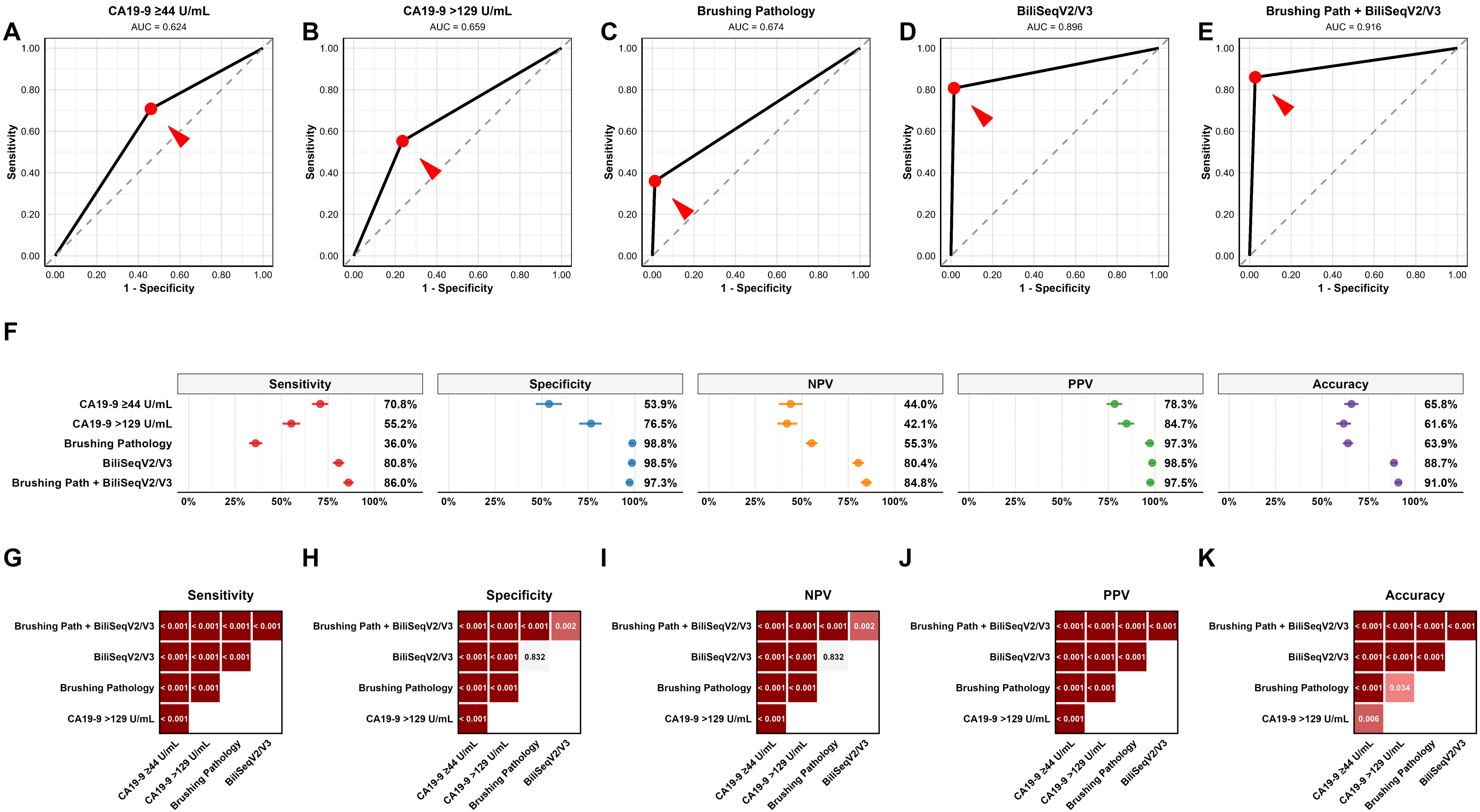
A
CA19-9 ≥44 U/mL
AUC = 0.624
B
CA19-9 >129 U/mL
AUC = 0.659
C
Brushing Pathology
AUC = 0.674
D
BiliSeqV2/V3
AUC = 0.896
E
Brushing Path + BiliSeqV2/V3
AUC = 0.916
Sensitivity
1 - Specificity
F
Sensitivity
Specificity
NPV
PPV
Accuracy
CA19-9 ≥44 U/mL 70.8% 53.9% 44.0% 78.3% 65.8%
CA19-9 >129 U/mL 55.2% 76.5% 42.1% 84.7% 61.6%
Brushing Pathology 36.0% 98.8% 55.3% 97.3% 63.9%
BiliSeqV2/V3 80.8% 98.5% 80.4% 98.5% 88.7%
Brushing Path + BiliSeqV2/V3 86.0% 97.3% 84.8% 97.5% 91.0%
G
H
I
J
K
Brushing Path + BiliSeqV2/V3
BiliSeqV2/V3
Brushing Pathology
CA19-9 >129 U/mL
CA19-9 ≥44 U/mL
Specificity: 0.002, 0.832
NPV: 0.002, 0.832
Accuracy: 0.034, 0.006
< 0.001

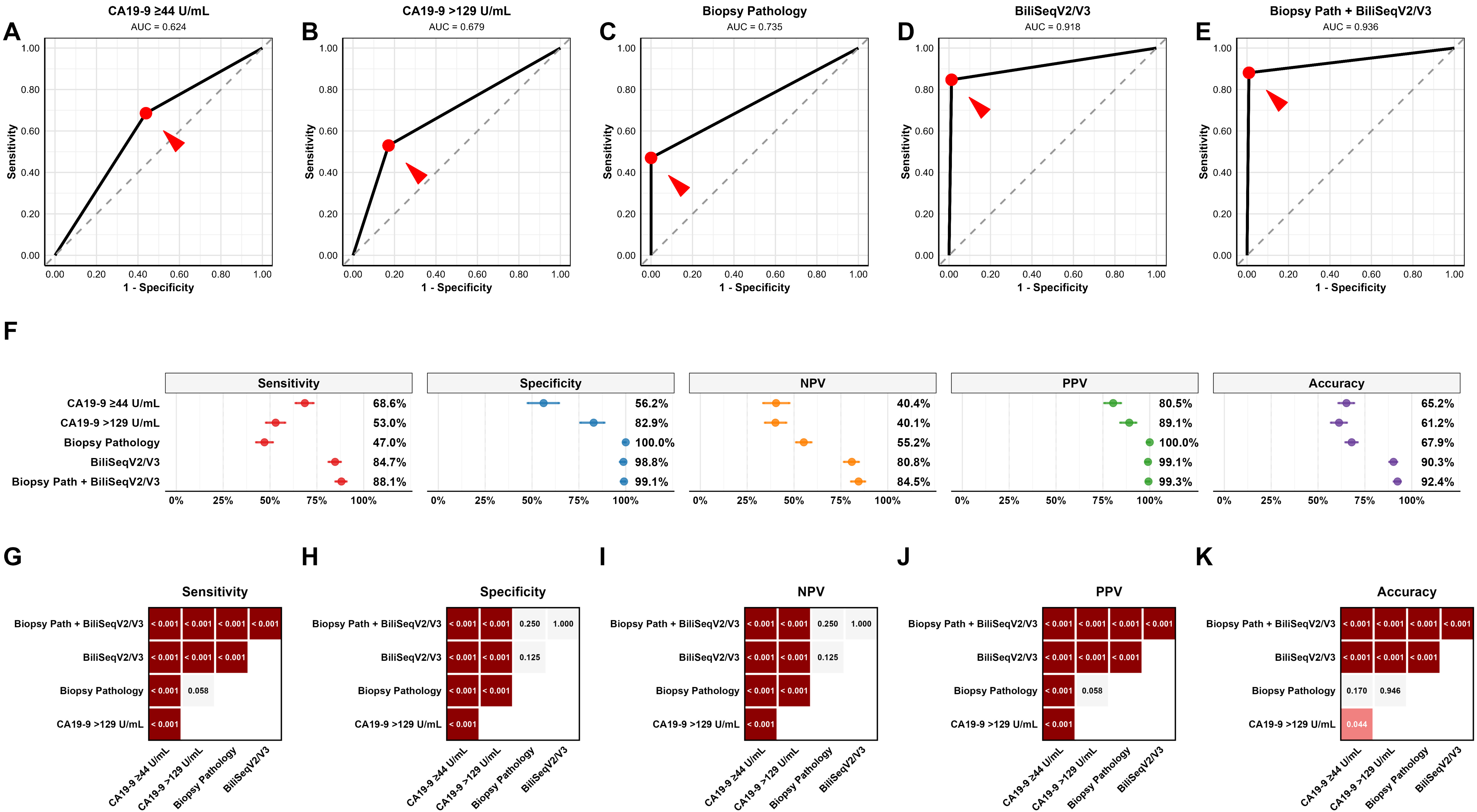
A
CA19-9 ≥44 U/mL
AUC = 0.624
B
CA19-9 >129 U/mL
AUC = 0.679
C
Biopsy Pathology
AUC = 0.735
D
BiliSeqV2/V3
AUC = 0.918
E
Biopsy Path + BiliSeqV2/V3
AUC = 0.936
Sensitivity
1 - Specificity
0.00
0.20
0.40
0.60
0.80
1.00
F
Sensitivity
Specificity
NPV
PPV
Accuracy
CA19-9 ≥44 U/mL
CA19-9 >129 U/mL
Biopsy Pathology
BiliSeqV2/V3
Biopsy Path + BiliSeqV2/V3
68.6%
53.0%
47.0%
84.7%
88.1%
56.2%
82.9%
100.0%
98.8%
99.1%
40.4%
40.1%
55.2%
80.8%
84.5%
80.5%
89.1%
100.0%
99.1%
99.3%
65.2%
61.2%
67.9%
90.3%
92.4%
0%
25%
50%
75%
100%
G
Sensitivity
H
Specificity
I
NPV
J
PPV
K
Accuracy
< 0.001
0.058
0.250
1.000
0.125
0.170
0.946
0.044

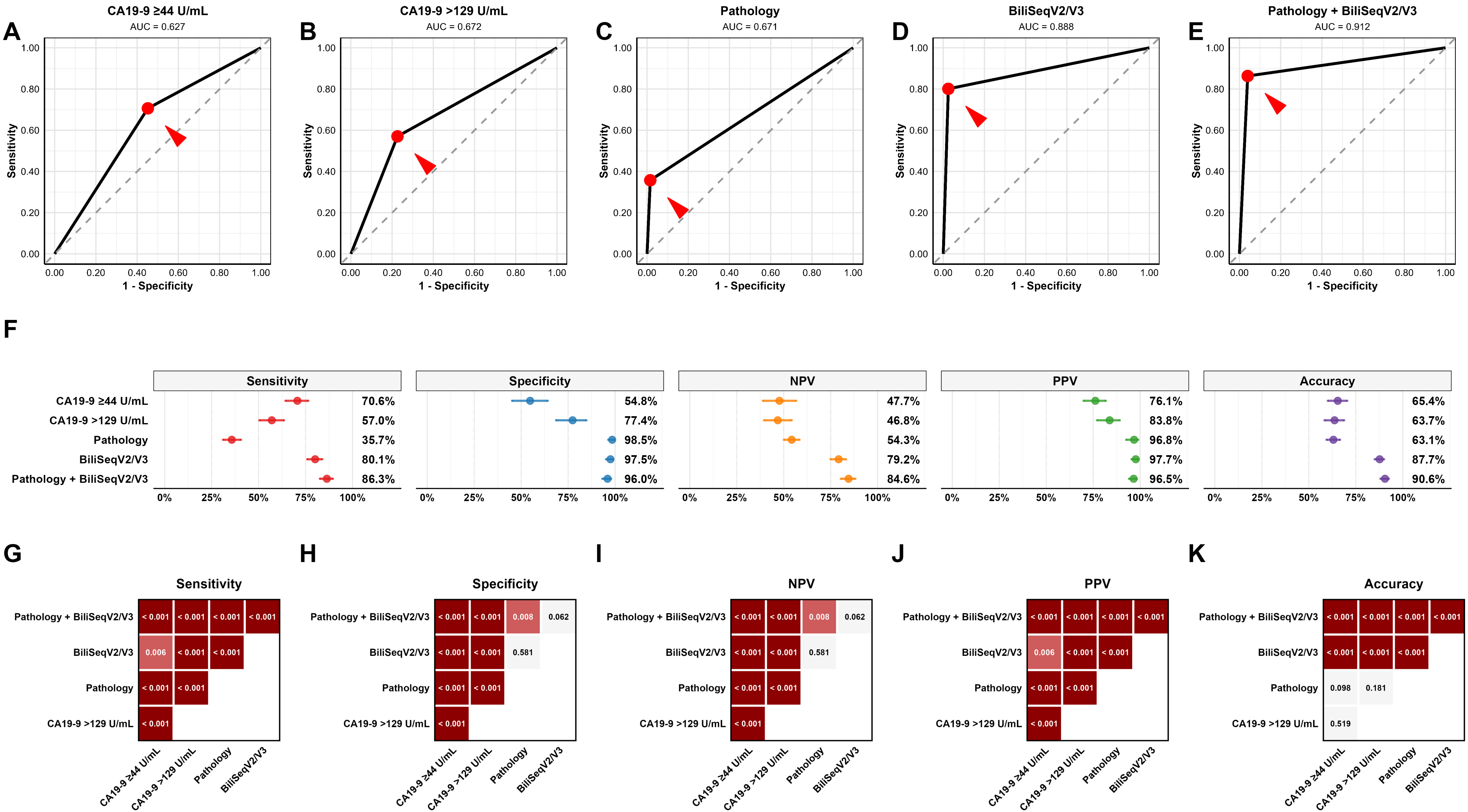
A
CA19-9 ≥44 U/mL
AUC = 0.627
B
CA19-9 >129 U/mL
AUC = 0.672
C
Pathology
AUC = 0.671
D
BiliSeqV2/V3
AUC = 0.888
E
Pathology + BiliSeqV2/V3
AUC = 0.912
Sensitivity
1 - Specificity
F
Sensitivity
Specificity
NPV
PPV
Accuracy
CA19-9 ≥44 U/mL
CA19-9 >129 U/mL
Pathology
BiliSeqV2/V3
Pathology + BiliSeqV2/V3
70.6%
57.0%
35.7%
80.1%
86.3%
54.8%
77.4%
98.5%
97.5%
96.0%
47.7%
46.8%
54.3%
79.2%
84.6%
76.1%
83.8%
96.8%
97.7%
96.5%
65.4%
63.7%
63.1%
87.7%
90.6%
G
Sensitivity
H
Specificity
I
NPV
J
PPV
K
Accuracy

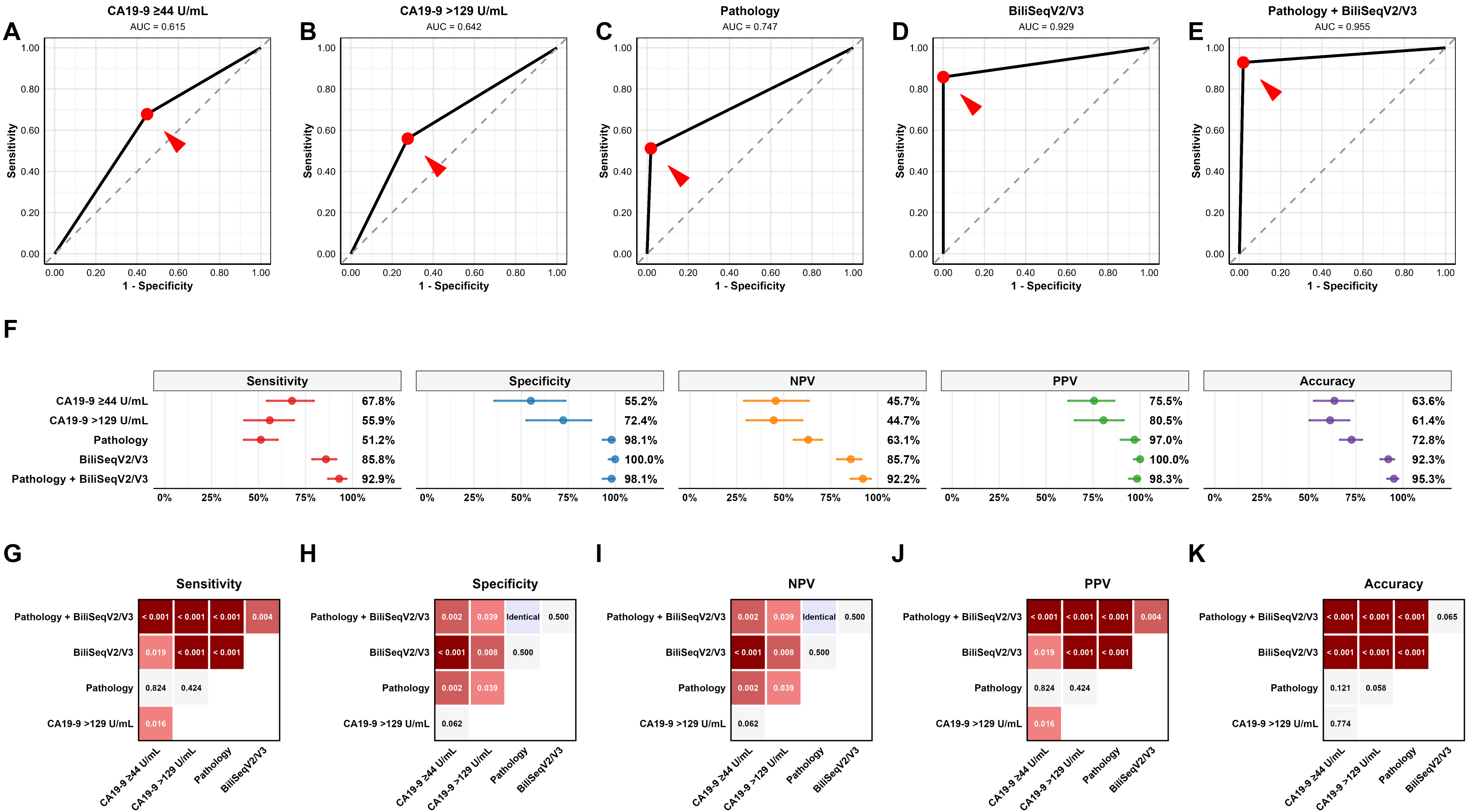
A
CA19-9 ≥44 U/mL
AUC = 0.615
B
CA19-9 >129 U/mL
AUC = 0.642
C
Pathology
AUC = 0.747
D
BiliSeqV2/V3
AUC = 0.929
E
Pathology + BiliSeqV2/V3
AUC = 0.955
Sensitivity
1 - Specificity
0.00
0.20
0.40
0.60
0.80
1.00
F
Sensitivity
Specificity
NPV
PPV
Accuracy
CA19-9 ≥44 U/mL
CA19-9 >129 U/mL
Pathology
BiliSeqV2/V3
Pathology + BiliSeqV2/V3
67.8%
55.9%
51.2%
85.8%
92.9%
55.2%
72.4%
98.1%
100.0%
98.1%
45.7%
44.7%
63.1%
85.7%
92.2%
75.5%
80.5%
97.0%
100.0%
98.3%
63.6%
61.4%
72.8%
92.3%
95.3%
0%
25%
50%
75%
100%
G
Sensitivity
Pathology + BiliSeqV2/V3 < 0.001 < 0.001 < 0.001 0.004
BiliSeqV2/V3 0.019 < 0.001 < 0.001
Pathology 0.824 0.424
CA19-9 >129 U/mL 0.016
H
Specificity
Pathology + BiliSeqV2/V3 0.002 0.039 Identical 0.500
BiliSeqV2/V3 < 0.001 0.008 0.500
Pathology 0.002 0.039
CA19-9 >129 U/mL 0.062
I
NPV
Pathology + BiliSeqV2/V3 0.002 0.039 Identical 0.500
BiliSeqV2/V3 < 0.001 0.008 0.500
Pathology 0.002 0.039
CA19-9 >129 U/mL 0.062
J
PPV
Pathology + BiliSeqV2/V3 < 0.001 < 0.001 < 0.001 0.004
BiliSeqV2/V3 0.019 < 0.001 < 0.001
Pathology 0.824 0.424
CA19-9 >129 U/mL 0.016
K
Accuracy
Pathology + BiliSeqV2/V3 < 0.001 < 0.001 < 0.001 0.065
BiliSeqV2/V3 < 0.001 < 0.001 < 0.001
Pathology 0.121 0.058
CA19-9 >129 U/mL 0.774
CA19-9 ≥44 U/mL
CA19-9 >129 U/mL
Pathology
BiliSeqV2/V3

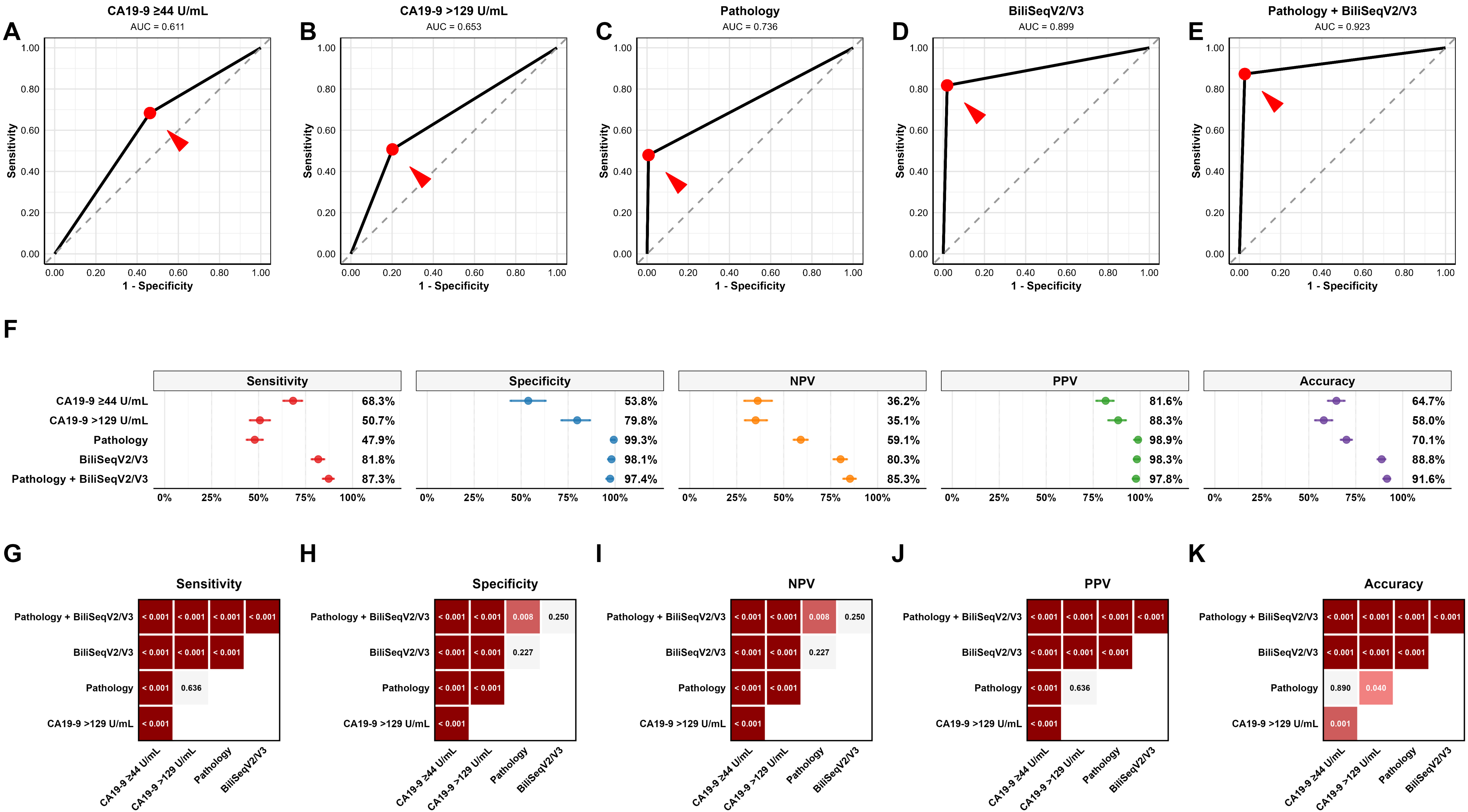
A
CA19-9 ≥44 U/mL
AUC = 0.611
B
CA19-9 >129 U/mL
AUC = 0.653
C
Pathology
AUC = 0.736
D
BiliSeqV2/V3
AUC = 0.899
E
Pathology + BiliSeqV2/V3
AUC = 0.923
Sensitivity
1 - Specificity
0.00
0.20
0.40
0.60
0.80
1.00
F
Sensitivity
Specificity
NPV
PPV
Accuracy
CA19-9 ≥44 U/mL
CA19-9 >129 U/mL
Pathology
BiliSeqV2/V3
Pathology + BiliSeqV2/V3
68.3%
50.7%
47.9%
81.8%
87.3%
53.8%
79.8%
99.3%
98.1%
97.4%
36.2%
35.1%
59.1%
80.3%
85.3%
81.6%
88.3%
98.9%
98.3%
97.8%
64.7%
58.0%
70.1%
88.8%
91.6%
0%
25%
50%
75%
100%
G
Sensitivity
H
Specificity
I
NPV
J
PPV
K
Accuracy
Pathology + BiliSeqV2/V3
BiliSeqV2/V3
Pathology
CA19-9 >129 U/mL
CA19-9 ≥44 U/mL
< 0.001
0.636
0.008
0.250
0.227
0.890
0.040
0.001

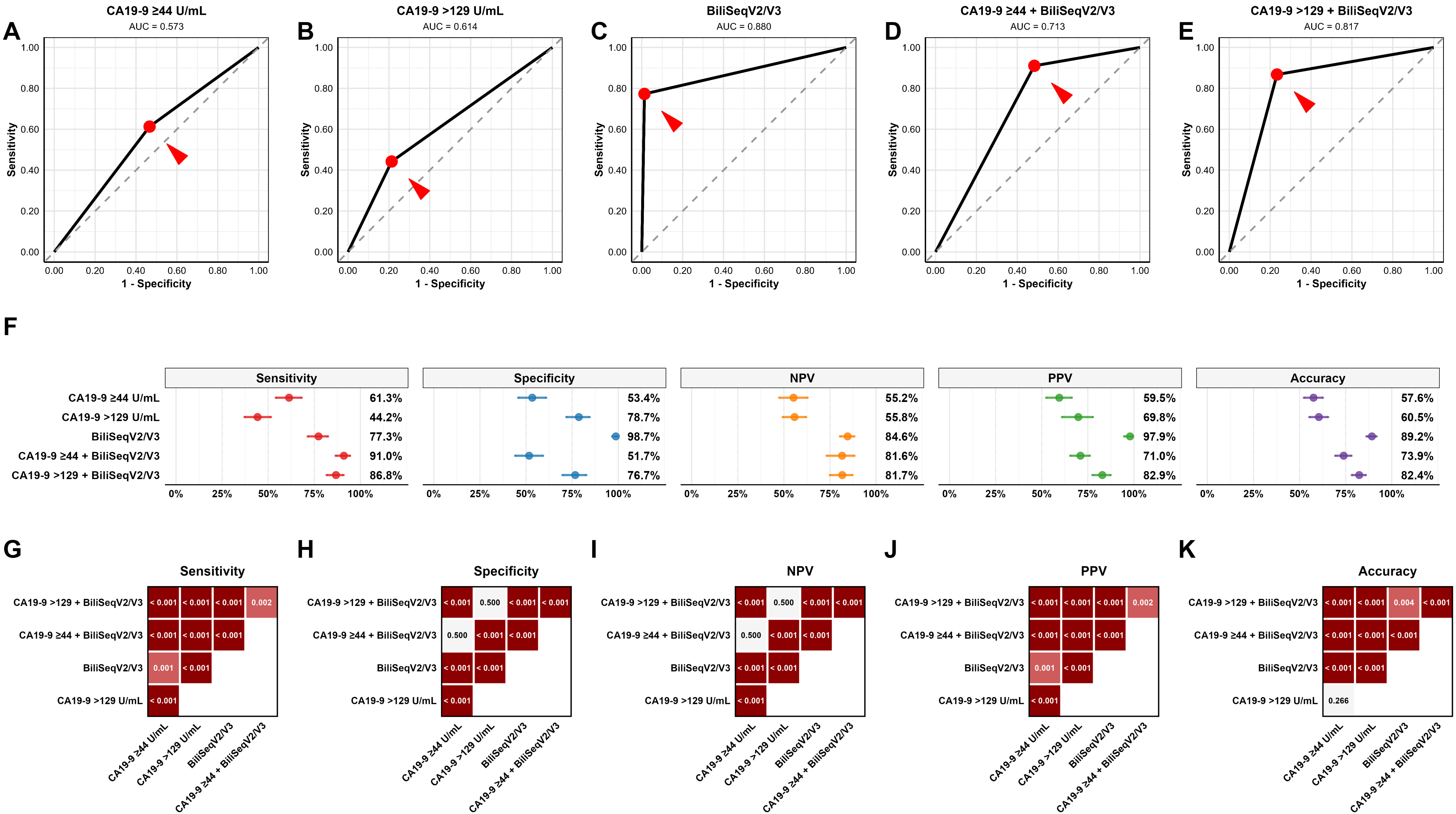
A
CA19-9 ≥44 U/mL
AUC = 0.573
B
CA19-9 >129 U/mL
AUC = 0.614
C
BiliSeqV2/V3
AUC = 0.880
D
CA19-9 ≥44 + BiliSeqV2/V3
AUC = 0.713
E
CA19-9 >129 + BiliSeqV2/V3
AUC = 0.817
Sensitivity
1 - Specificity
0.00
0.20
0.40
0.60
0.80
1.00
F
Sensitivity
Specificity
NPV
PPV
Accuracy
CA19-9 ≥44 U/mL
CA19-9 >129 U/mL
BiliSeqV2/V3
CA19-9 ≥44 + BiliSeqV2/V3
CA19-9 >129 + BiliSeqV2/V3
61.3%
44.2%
77.3%
91.0%
86.8%
53.4%
78.7%
98.7%
51.7%
76.7%
55.2%
55.8%
84.6%
81.6%
81.7%
59.5%
69.8%
97.9%
71.0%
82.9%
57.6%
60.5%
89.2%
73.9%
82.4%
0%
25%
50%
75%
100%
G
Sensitivity
CA19-9 >129 + BiliSeqV2/V3: < 0.001, < 0.001, < 0.001, 0.002
CA19-9 ≥44 + BiliSeqV2/V3: < 0.001, < 0.001, < 0.001
BiliSeqV2/V3: 0.001, < 0.001
CA19-9 >129 U/mL: < 0.001
H
Specificity
CA19-9 >129 + BiliSeqV2/V3: < 0.001, 0.500, < 0.001, < 0.001
CA19-9 ≥44 + BiliSeqV2/V3: 0.500, < 0.001, < 0.001
BiliSeqV2/V3: < 0.001, < 0.001
CA19-9 >129 U/mL: < 0.001
I
NPV
CA19-9 >129 + BiliSeqV2/V3: < 0.001, 0.500, < 0.001, < 0.001
CA19-9 ≥44 + BiliSeqV2/V3: 0.500, < 0.001, < 0.001
BiliSeqV2/V3: < 0.001, < 0.001
CA19-9 >129 U/mL: < 0.001
J
PPV
CA19-9 >129 + BiliSeqV2/V3: < 0.001, < 0.001, < 0.001, 0.002
CA19-9 ≥44 + BiliSeqV2/V3: < 0.001, < 0.001, < 0.001
BiliSeqV2/V3: 0.001, < 0.001
CA19-9 >129 U/mL: < 0.001
K
Accuracy
CA19-9 >129 + BiliSeqV2/V3: < 0.001, < 0.001, 0.004, < 0.001
CA19-9 ≥44 + BiliSeqV2/V3: < 0.001, < 0.001, < 0.001
BiliSeqV2/V3: < 0.001, < 0.001
CA19-9 >129 U/mL: 0.266
CA19-9 ≥44 U/mL
CA19-9 >129 U/mL
BiliSeqV2/V3
CA19-9 ≥44 + BiliSeqV2/V3

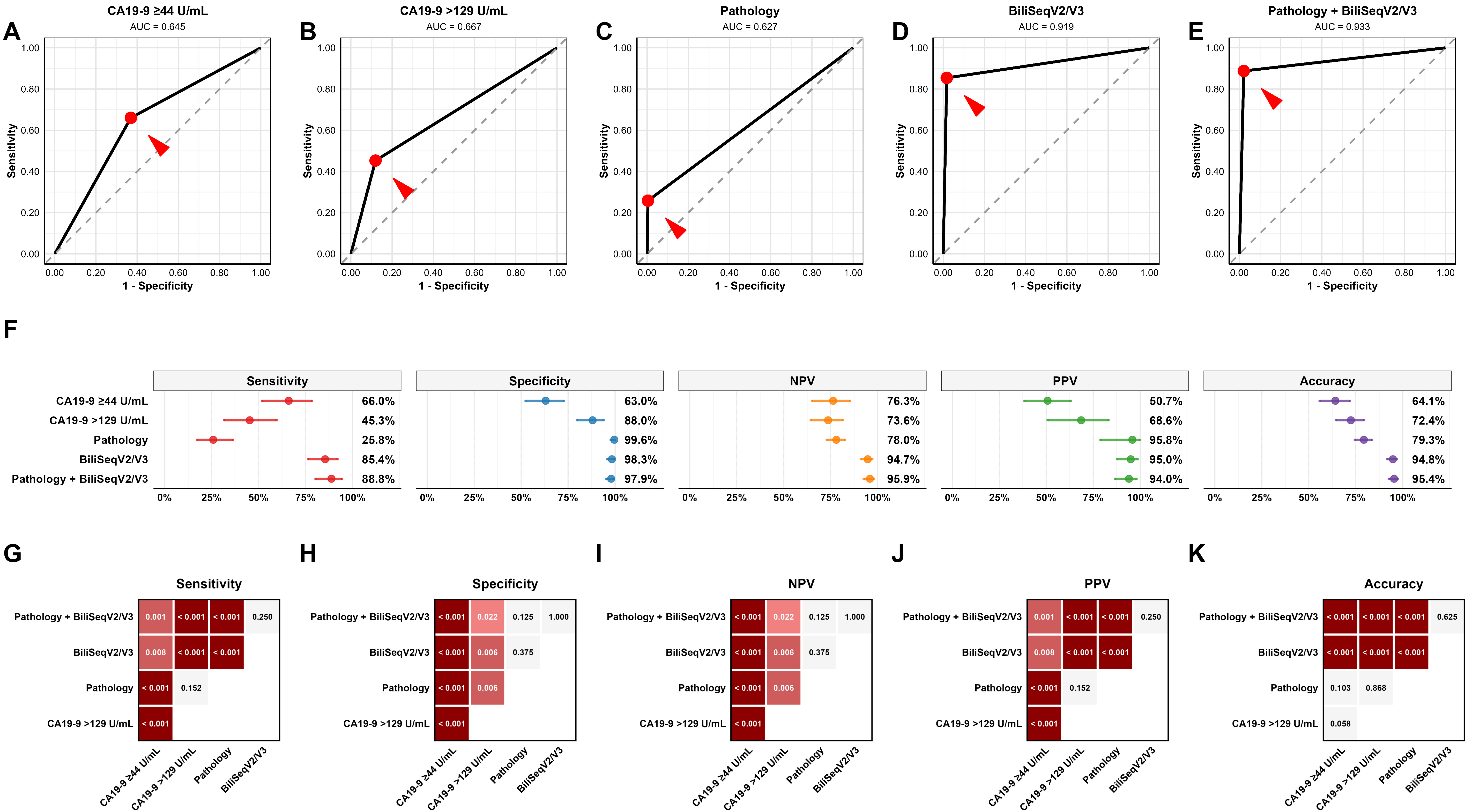
A
CA19-9 ≥44 U/mL
AUC = 0.645
B
CA19-9 >129 U/mL
AUC = 0.667
C
Pathology
AUC = 0.627
D
BiliSeqV2/V3
AUC = 0.919
E
Pathology + BiliSeqV2/V3
AUC = 0.933
Sensitivity
1 - Specificity
0.00
0.20
0.40
0.60
0.80
1.00
F
Sensitivity
Specificity
NPV
PPV
Accuracy
CA19-9 ≥44 U/mL
CA19-9 >129 U/mL
Pathology
BiliSeqV2/V3
Pathology + BiliSeqV2/V3
66.0%
45.3%
25.8%
85.4%
88.8%
63.0%
88.0%
99.6%
98.3%
97.9%
76.3%
73.6%
78.0%
94.7%
95.9%
50.7%
68.6%
95.8%
95.0%
94.0%
64.1%
72.4%
79.3%
94.8%
95.4%
0%
25%
50%
75%
100%
G
Sensitivity
Pathology + BiliSeqV2/V3: 0.001, < 0.001, < 0.001, 0.250
BiliSeqV2/V3: 0.008, < 0.001, < 0.001
Pathology: < 0.001, 0.152
CA19-9 >129 U/mL: < 0.001
H
Specificity
Pathology + BiliSeqV2/V3: < 0.001, 0.022, 0.125, 1.000
BiliSeqV2/V3: < 0.001, 0.006, 0.375
Pathology: < 0.001, 0.006
CA19-9 >129 U/mL: < 0.001
I
NPV
Pathology + BiliSeqV2/V3: < 0.001, 0.022, 0.125, 1.000
BiliSeqV2/V3: < 0.001, 0.006, 0.375
Pathology: < 0.001, 0.006
CA19-9 >129 U/mL: < 0.001
J
PPV
Pathology + BiliSeqV2/V3: 0.001, < 0.001, < 0.001, 0.250
BiliSeqV2/V3: 0.008, < 0.001, < 0.001
Pathology: < 0.001, 0.152
CA19-9 >129 U/mL: < 0.001
K
Accuracy
Pathology + BiliSeqV2/V3: < 0.001, < 0.001, < 0.001, 0.625
BiliSeqV2/V3: < 0.001, < 0.001, < 0.001
Pathology: 0.103, 0.868
CA19-9 >129 U/mL: 0.058
CA19-9 ≥44 U/mL
CA19-9 >129 U/mL
Pathology
BiliSeqV2/V3

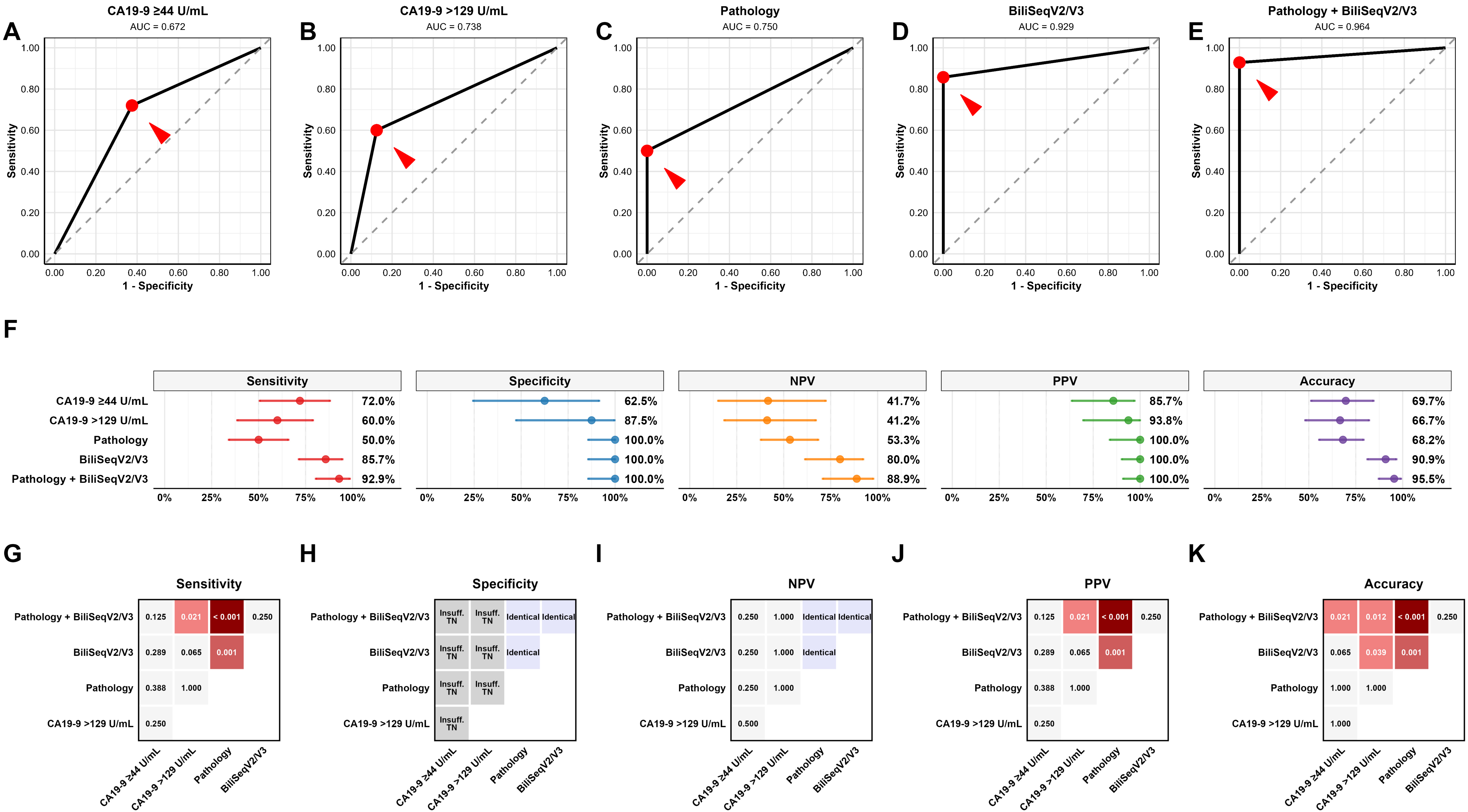
A
CA19-9 ≥44 U/mL
AUC = 0.672
B
CA19-9 >129 U/mL
AUC = 0.738
C
Pathology
AUC = 0.750
D
BiliSeqV2/V3
AUC = 0.929
E
Pathology + BiliSeqV2/V3
AUC = 0.964
Sensitivity
1 - Specificity
F
Sensitivity
Specificity
NPV
PPV
Accuracy
CA19-9 ≥44 U/mL
CA19-9 >129 U/mL
Pathology
BiliSeqV2/V3
Pathology + BiliSeqV2/V3
72.0%
60.0%
50.0%
85.7%
92.9%
62.5%
87.5%
100.0%
100.0%
100.0%
41.7%
41.2%
53.3%
80.0%
88.9%
85.7%
93.8%
100.0%
100.0%
100.0%
69.7%
66.7%
68.2%
90.9%
95.5%
G
Sensitivity
H
Specificity
I
NPV
J
PPV
K
Accuracy
Insuff. TN
Identical

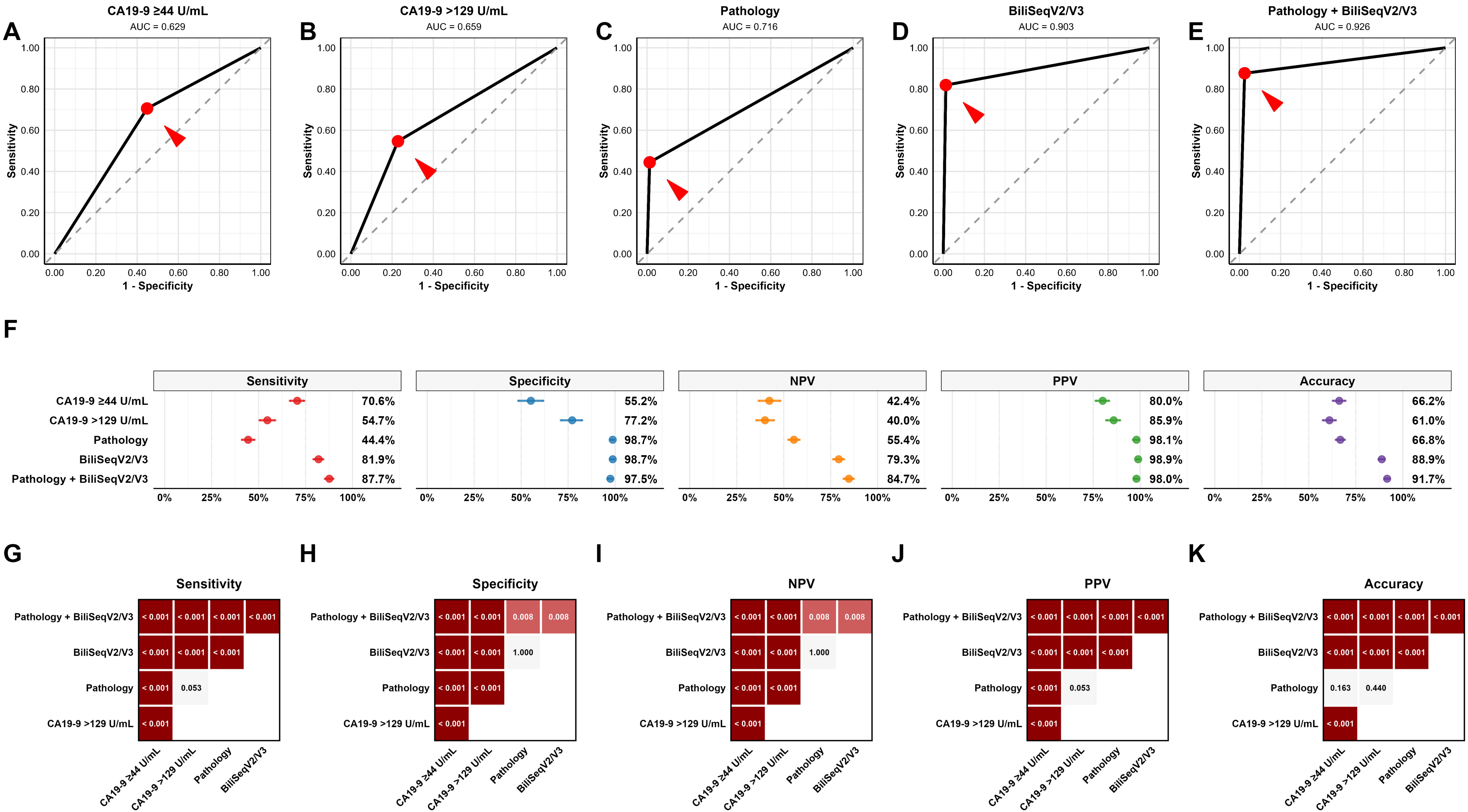
A
CA19-9 ≥44 U/mL
AUC = 0.629
B
CA19-9 >129 U/mL
AUC = 0.659
C
Pathology
AUC = 0.716
D
BiliSeqV2/V3
AUC = 0.903
E
Pathology + BiliSeqV2/V3
AUC = 0.926
Sensitivity
1 - Specificity
F
Sensitivity
Specificity
NPV
PPV
Accuracy
CA19-9 ≥44 U/mL
CA19-9 >129 U/mL
Pathology
BiliSeqV2/V3
Pathology + BiliSeqV2/V3
70.6%
54.7%
44.4%
81.9%
87.7%
55.2%
77.2%
98.7%
98.7%
97.5%
42.4%
40.0%
55.4%
79.3%
84.7%
80.0%
85.9%
98.1%
98.9%
98.0%
66.2%
61.0%
66.8%
88.9%
91.7%
G
H
I
J
K

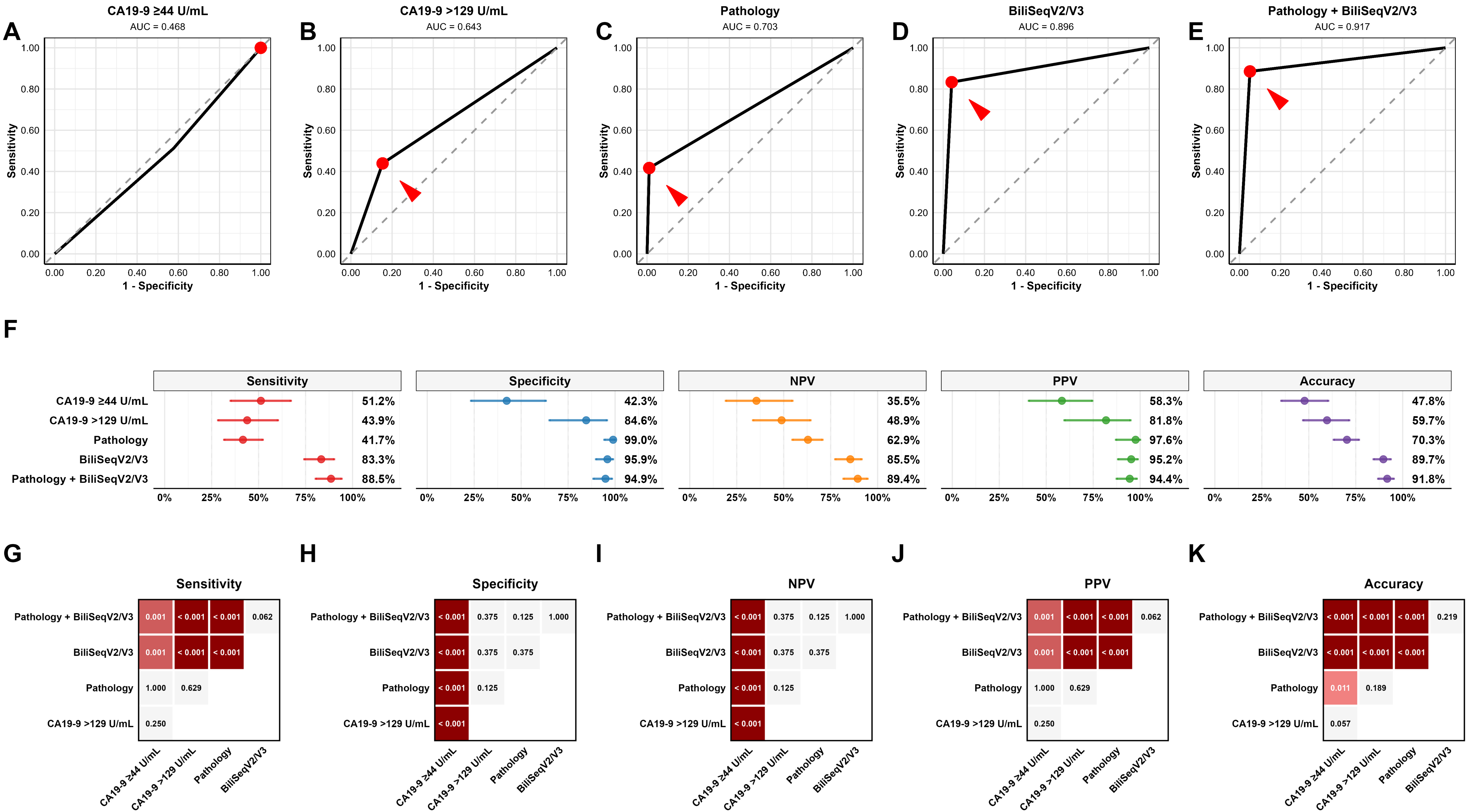
A
CA19-9 ≥44 U/mL
AUC = 0.468
B
CA19-9 >129 U/mL
AUC = 0.643
C
Pathology
AUC = 0.703
D
BiliSeqV2/V3
AUC = 0.896
E
Pathology + BiliSeqV2/V3
AUC = 0.917
Sensitivity
1 - Specificity
F
Sensitivity
Specificity
NPV
PPV
Accuracy
CA19-9 ≥44 U/mL
CA19-9 >129 U/mL
Pathology
BiliSeqV2/V3
Pathology + BiliSeqV2/V3
51.2% 43.9% 41.7% 83.3% 88.5%
42.3% 84.6% 99.0% 95.9% 94.9%
35.5% 48.9% 62.9% 85.5% 89.4%
58.3% 81.8% 97.6% 95.2% 94.4%
47.8% 59.7% 70.3% 89.7% 91.8%
G
Sensitivity
Pathology + BiliSeqV2/V3: 0.001, < 0.001, < 0.001, 0.062
BiliSeqV2/V3: 0.001, < 0.001, < 0.001
Pathology: 1.000, 0.629
CA19-9 >129 U/mL: 0.250
H
Specificity
Pathology + BiliSeqV2/V3: < 0.001, 0.375, 0.125, 1.000
BiliSeqV2/V3: < 0.001, 0.375, 0.375
Pathology: < 0.001, 0.125
CA19-9 >129 U/mL: < 0.001
I
NPV
Pathology + BiliSeqV2/V3: < 0.001, 0.375, 0.125, 1.000
BiliSeqV2/V3: < 0.001, 0.375, 0.375
Pathology: < 0.001, 0.125
CA19-9 >129 U/mL: < 0.001
J
PPV
Pathology + BiliSeqV2/V3: 0.001, < 0.001, < 0.001, 0.062
BiliSeqV2/V3: 0.001, < 0.001, < 0.001
Pathology: 1.000, 0.629
CA19-9 >129 U/mL: 0.250
K
Accuracy
Pathology + BiliSeqV2/V3: < 0.001, < 0.001, < 0.001, 0.219
BiliSeqV2/V3: < 0.001, < 0.001, < 0.001
Pathology: 0.011, 0.189
CA19-9 >129 U/mL: 0.057
CA19-9 ≥44 U/mL
CA19-9 >129 U/mL
Pathology
BiliSeqV2/V3

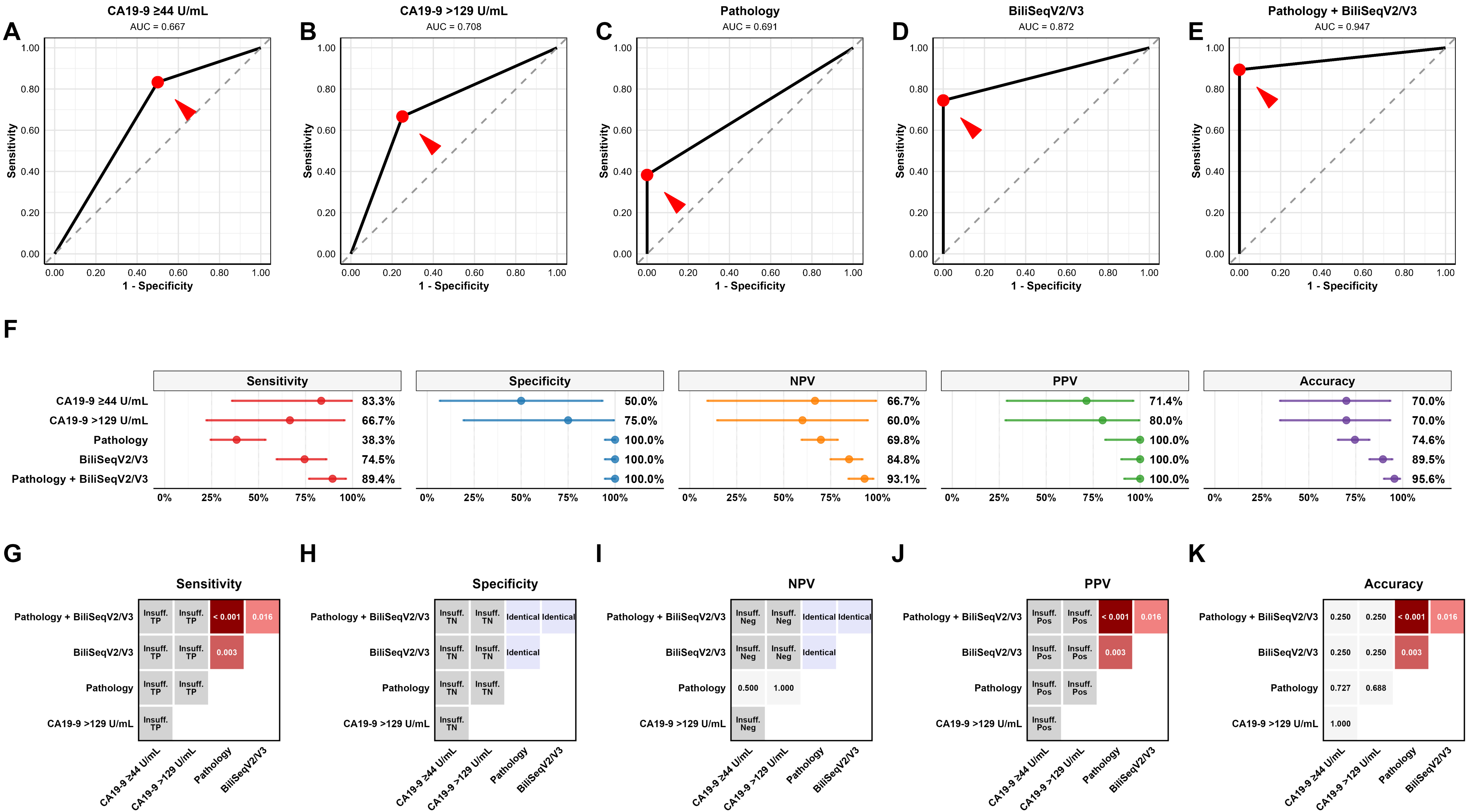
A
CA19-9 ≥44 U/mL
AUC = 0.667
B
CA19-9 >129 U/mL
AUC = 0.708
C
Pathology
AUC = 0.691
D
BiliSeqV2/V3
AUC = 0.872
E
Pathology + BiliSeqV2/V3
AUC = 0.947
Sensitivity
1 - Specificity
F
Sensitivity
Specificity
NPV
PPV
Accuracy
CA19-9 ≥44 U/mL
CA19-9 >129 U/mL
Pathology
BiliSeqV2/V3
Pathology + BiliSeqV2/V3
83.3%
66.7%
38.3%
74.5%
89.4%
50.0%
75.0%
100.0%
100.0%
100.0%
66.7%
60.0%
69.8%
84.8%
93.1%
71.4%
80.0%
100.0%
100.0%
100.0%
70.0%
70.0%
74.6%
89.5%
95.6%
G
Sensitivity
H
Specificity
I
NPV
J
PPV
K
Accuracy
Insuff. TP
Insuff. TN
Insuff. Neg
Insuff. Pos
< 0.001
0.016
0.003
Identical
0.500
1.000
0.250
0.727
0.688

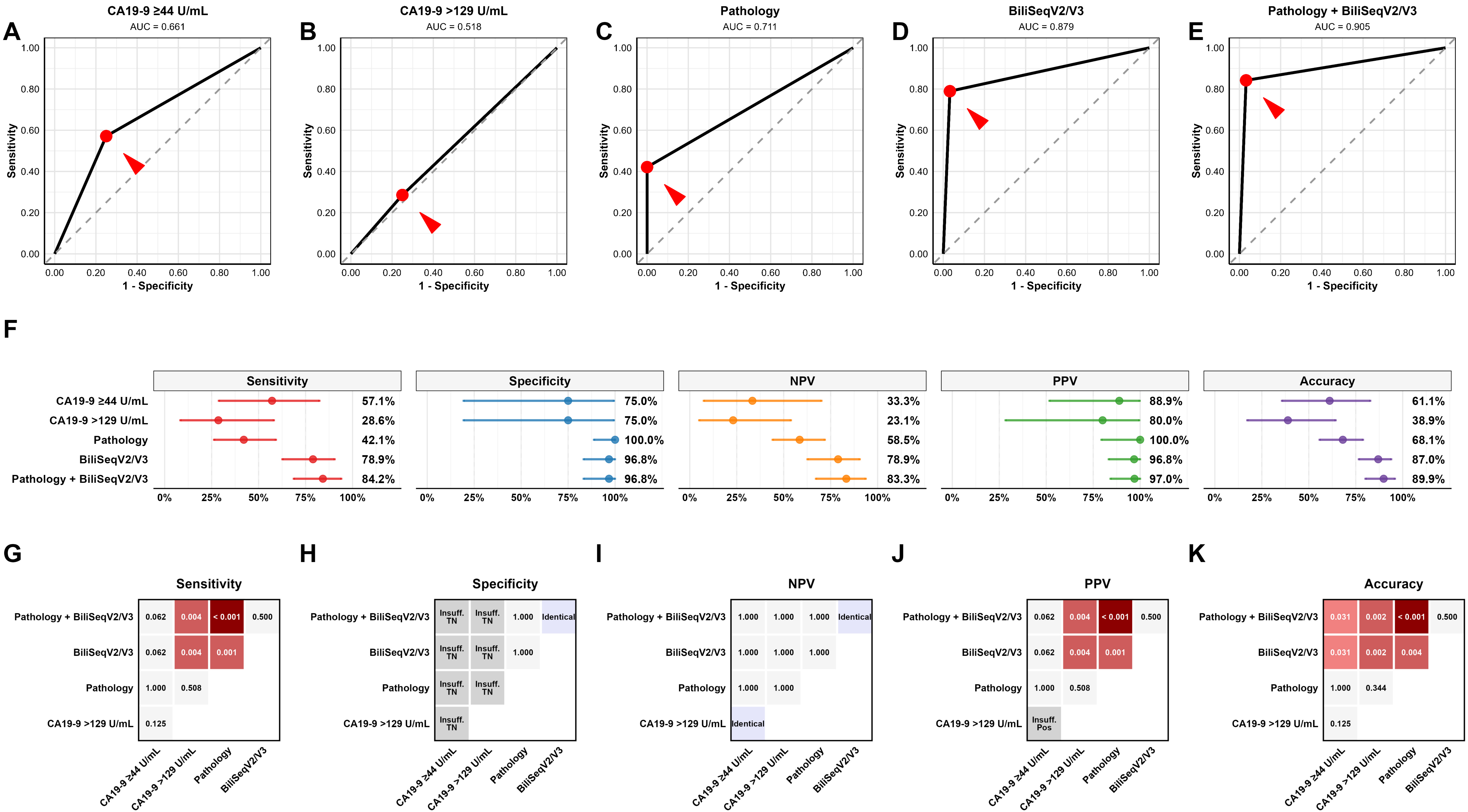
A
CA19-9 ≥44 U/mL
AUC = 0.661
B
CA19-9 >129 U/mL
AUC = 0.518
C
Pathology
AUC = 0.711
D
BiliSeqV2/V3
AUC = 0.879
E
Pathology + BiliSeqV2/V3
AUC = 0.905
Sensitivity
1 - Specificity
0.00 0.20 0.40 0.60 0.80 1.00
F
Sensitivity
Specificity
NPV
PPV
Accuracy
CA19-9 ≥44 U/mL 57.1% 75.0% 33.3% 88.9% 61.1%
CA19-9 >129 U/mL 28.6% 75.0% 23.1% 80.0% 38.9%
Pathology 42.1% 100.0% 58.5% 100.0% 68.1%
BiliSeqV2/V3 78.9% 96.8% 78.9% 96.8% 87.0%
Pathology + BiliSeqV2/V3 84.2% 96.8% 83.3% 97.0% 89.9%
0% 25% 50% 75% 100%
G
Sensitivity
Pathology + BiliSeqV2/V3 0.062 0.004 < 0.001 0.500
BiliSeqV2/V3 0.062 0.004 0.001
Pathology 1.000 0.508
CA19-9 >129 U/mL 0.125
H
Specificity
Pathology + BiliSeqV2/V3 Insuff. TN Insuff. TN 1.000 Identical
BiliSeqV2/V3 Insuff. TN Insuff. TN 1.000
Pathology Insuff. TN Insuff. TN
CA19-9 >129 U/mL Insuff. TN
I
NPV
Pathology + BiliSeqV2/V3 1.000 1.000 1.000 Identical
BiliSeqV2/V3 1.000 1.000 1.000
Pathology 1.000 1.000
CA19-9 >129 U/mL Identical
J
PPV
Pathology + BiliSeqV2/V3 0.062 0.004 < 0.001 0.500
BiliSeqV2/V3 0.062 0.004 0.001
Pathology 1.000 0.508
CA19-9 >129 U/mL Insuff. Pos
K
Accuracy
Pathology + BiliSeqV2/V3 0.031 0.002 < 0.001 0.500
BiliSeqV2/V3 0.031 0.002 0.004
Pathology 1.000 0.344
CA19-9 >129 U/mL 0.125
CA19-9 ≥44 U/mL
CA19-9 >129 U/mL
Pathology
BiliSeqV2/V3

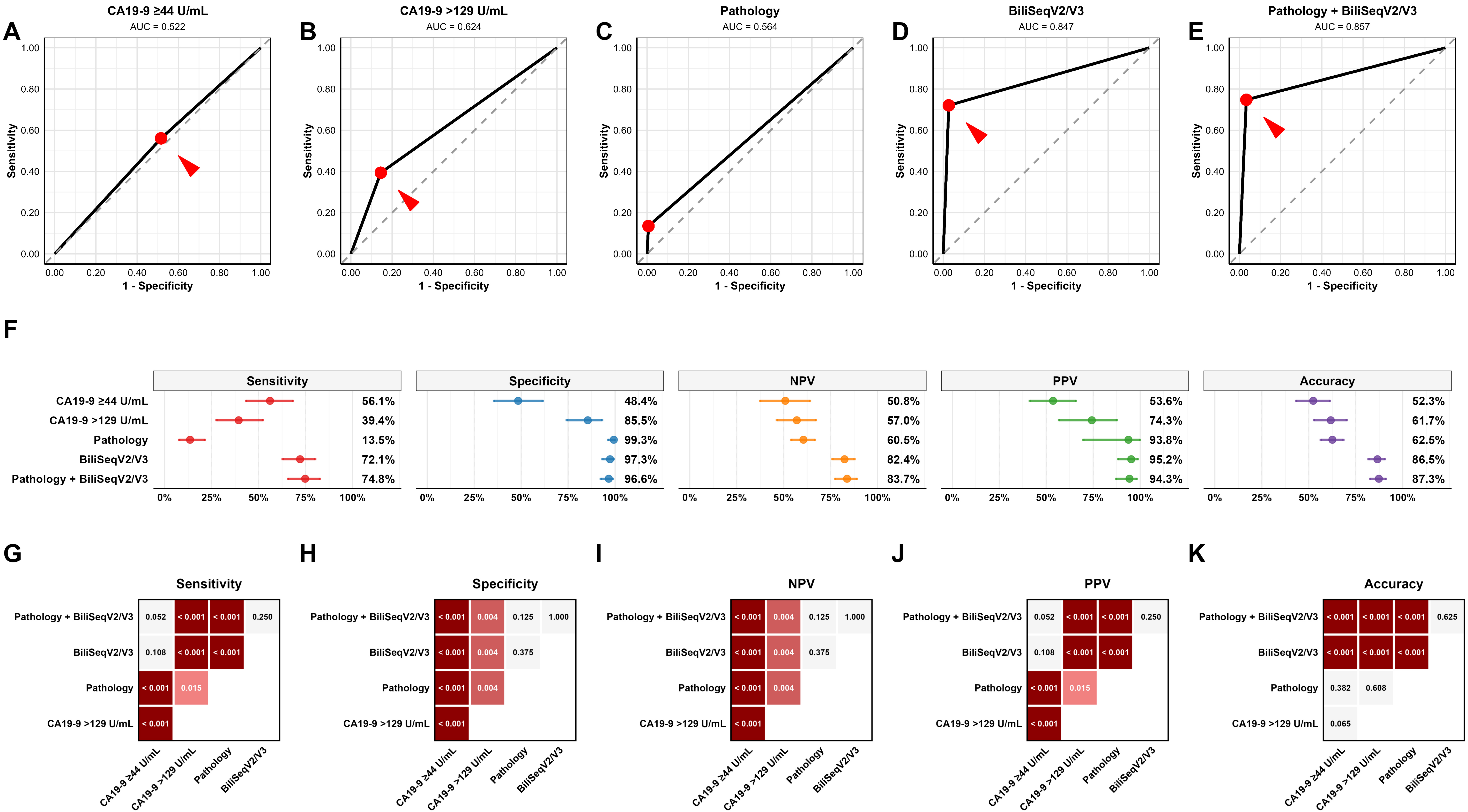
A
CA19-9 ≥44 U/mL
AUC = 0.522
B
CA19-9 >129 U/mL
AUC = 0.624
C
Pathology
AUC = 0.564
D
BiliSeqV2/V3
AUC = 0.847
E
Pathology + BiliSeqV2/V3
AUC = 0.857
Sensitivity
1 - Specificity
0.00
0.20
0.40
0.60
0.80
1.00
F
Sensitivity
Specificity
NPV
PPV
Accuracy
CA19-9 ≥44 U/mL
CA19-9 >129 U/mL
Pathology
BiliSeqV2/V3
Pathology + BiliSeqV2/V3
56.1% 39.4% 13.5% 72.1% 74.8%
48.4% 85.5% 99.3% 97.3% 96.6%
50.8% 57.0% 60.5% 82.4% 83.7%
53.6% 74.3% 93.8% 95.2% 94.3%
52.3% 61.7% 62.5% 86.5% 87.3%
0% 25% 50% 75% 100%
G
Sensitivity
Pathology + BiliSeqV2/V3 0.052 < 0.001 < 0.001 0.250
BiliSeqV2/V3 0.108 < 0.001 < 0.001
Pathology < 0.001 0.015
CA19-9 >129 U/mL < 0.001
H
Specificity
Pathology + BiliSeqV2/V3 < 0.001 0.004 0.125 1.000
BiliSeqV2/V3 < 0.001 0.004 0.375
Pathology < 0.001 0.004
CA19-9 >129 U/mL < 0.001
I
NPV
Pathology + BiliSeqV2/V3 < 0.001 0.004 0.125 1.000
BiliSeqV2/V3 < 0.001 0.004 0.375
Pathology < 0.001 0.004
CA19-9 >129 U/mL < 0.001
J
PPV
Pathology + BiliSeqV2/V3 0.052 < 0.001 < 0.001 0.250
BiliSeqV2/V3 0.108 < 0.001 < 0.001
Pathology < 0.001 0.015
CA19-9 >129 U/mL < 0.001
K
Accuracy
Pathology + BiliSeqV2/V3 < 0.001 < 0.001 < 0.001 0.625
BiliSeqV2/V3 < 0.001 < 0.001 < 0.001
Pathology 0.382 0.608
CA19-9 >129 U/mL 0.065
CA19-9 ≥44 U/mL
CA19-9 >129 U/mL
Pathology
BiliSeqV2/V3

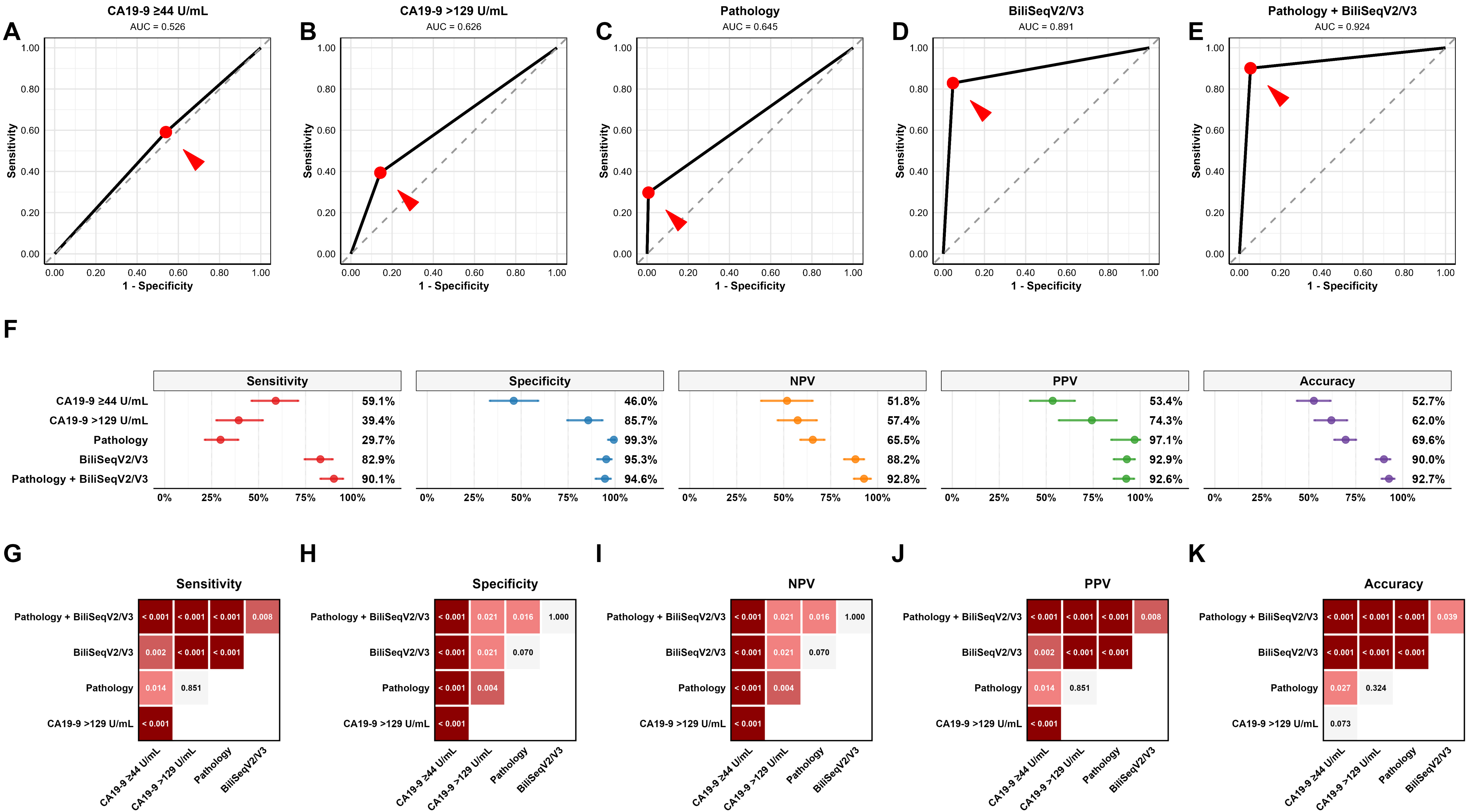
A
CA19-9 ≥44 U/mL
AUC = 0.526
B
CA19-9 >129 U/mL
AUC = 0.626
C
Pathology
AUC = 0.645
D
BiliSeqV2/V3
AUC = 0.891
E
Pathology + BiliSeqV2/V3
AUC = 0.924
Sensitivity
1 - Specificity
0.00
0.20
0.40
0.60
0.80
1.00
F
Sensitivity
Specificity
NPV
PPV
Accuracy
CA19-9 ≥44 U/mL
CA19-9 >129 U/mL
Pathology
BiliSeqV2/V3
Pathology + BiliSeqV2/V3
59.1%
39.4%
29.7%
82.9%
90.1%
46.0%
85.7%
99.3%
95.3%
94.6%
51.8%
57.4%
65.5%
88.2%
92.8%
53.4%
74.3%
97.1%
92.9%
92.6%
52.7%
62.0%
69.6%
90.0%
92.7%
0%
25%
50%
75%
100%
G
Sensitivity
H
Specificity
I
NPV
J
PPV
K
Accuracy
Pathology + BiliSeqV2/V3
BiliSeqV2/V3
Pathology
CA19-9 >129 U/mL
CA19-9 ≥44 U/mL
CA19-9 >129 U/mL
Pathology
BiliSeqV2/V3
Sensitivity: < 0.001, < 0.001, < 0.001, 0.008; 0.002, < 0.001, < 0.001; 0.014, 0.851; < 0.001
Specificity: < 0.001, 0.021, 0.016, 1.000; < 0.001, 0.021, 0.070; < 0.001, 0.004; < 0.001
NPV: < 0.001, 0.021, 0.016, 1.000; < 0.001, 0.021, 0.070; < 0.001, 0.004; < 0.001
PPV: < 0.001, < 0.001, < 0.001, 0.008; 0.002, < 0.001, < 0.001; 0.014, 0.851; < 0.001
Accuracy: < 0.001, < 0.001, < 0.001, 0.039; < 0.001, < 0.001, < 0.001; 0.027, 0.324; 0.073

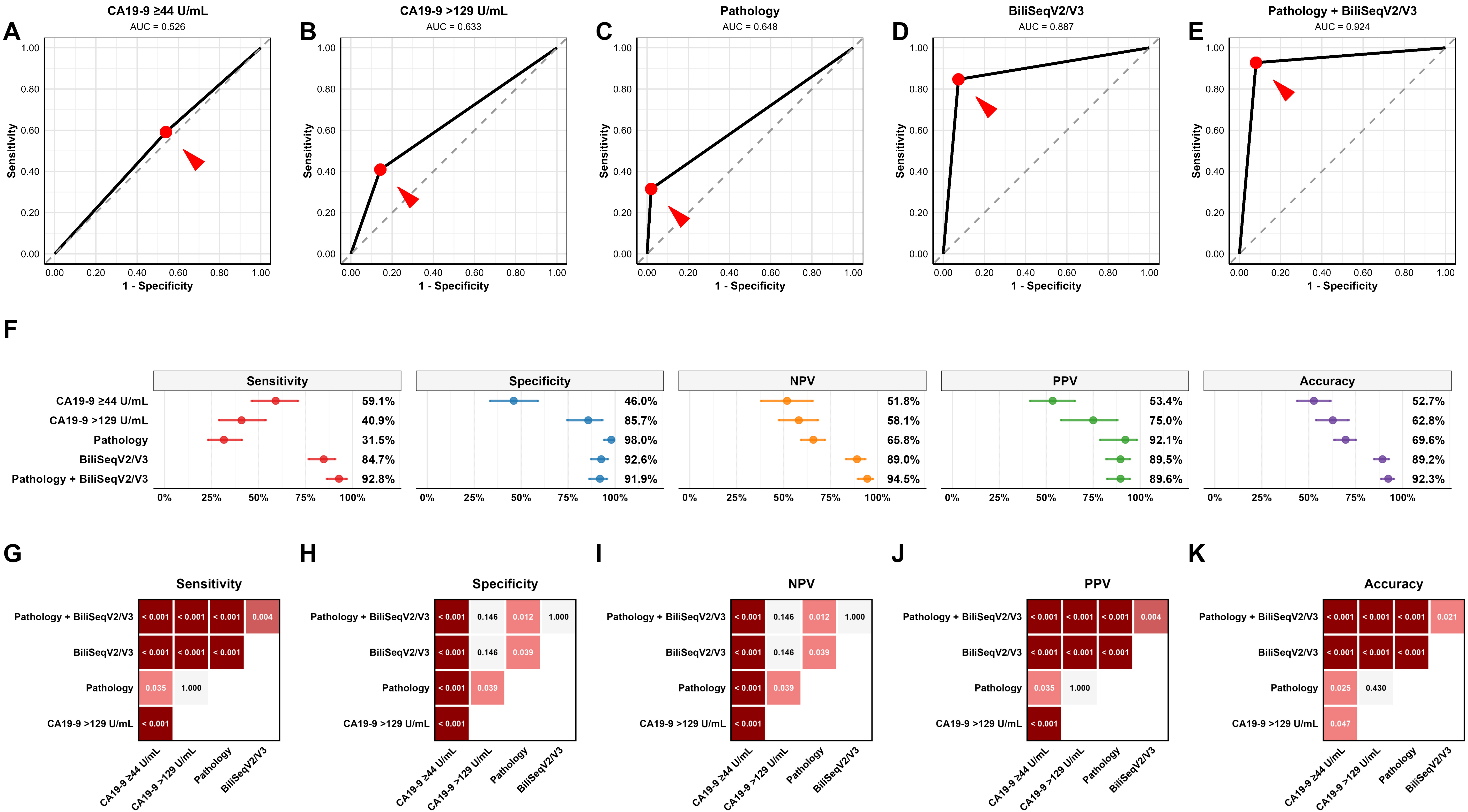

Sensitivity (G)	CA19-9 ≥44 U/mL	CA19-9 >129 U/mL	Pathology	BiliSeqV2/V3
Pathology + BiliSeqV2/V3	< 0.001	< 0.001	< 0.001	0.004
BiliSeqV2/V3	< 0.001	< 0.001	< 0.001	
Pathology	0.035	1.000		
CA19-9 >129 U/mL	< 0.001			

Specificity (H)	CA19-9 ≥44 U/mL	CA19-9 >129 U/mL	Pathology	BiliSeqV2/V3
Pathology + BiliSeqV2/V3	< 0.001	0.146	0.012	1.000
BiliSeqV2/V3	< 0.001	0.146	0.039	
Pathology	< 0.001	0.039		
CA19-9 >129 U/mL	< 0.001			

NPV (I)	CA19-9 ≥44 U/mL	CA19-9 >129 U/mL	Pathology	BiliSeqV2/V3
Pathology + BiliSeqV2/V3	< 0.001	0.146	0.012	1.000
BiliSeqV2/V3	< 0.001	0.146	0.039	
Pathology	< 0.001	0.039		
CA19-9 >129 U/mL	< 0.001			

PPV (J)	CA19-9 ≥44 U/mL	CA19-9 >129 U/mL	Pathology	BiliSeqV2/V3
Pathology + BiliSeqV2/V3	< 0.001	< 0.001	< 0.001	0.004
BiliSeqV2/V3	< 0.001	< 0.001	< 0.001	
Pathology	0.035	1.000		
CA19-9 >129 U/mL	< 0.001			

Accuracy (K)	CA19-9 ≥44 U/mL	CA19-9 >129 U/mL	Pathology	BiliSeqV2/V3
Pathology + BiliSeqV2/V3	< 0.001	< 0.001	< 0.001	0.021
BiliSeqV2/V3	< 0.001	< 0.001	< 0.001	
Pathology	0.025	0.430		
CA19-9 >129 U/mL	0.047			

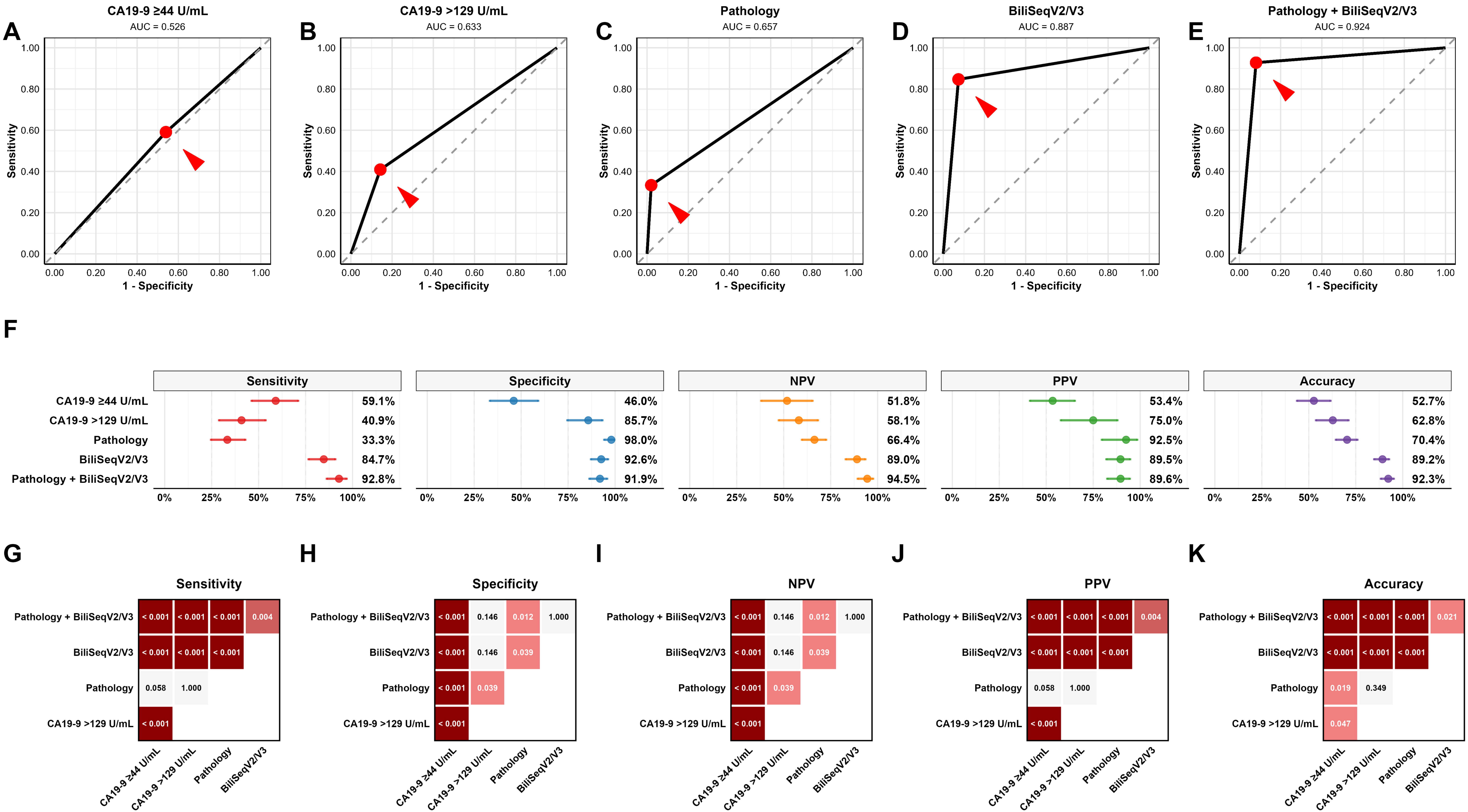

A
CA19-9 ≥44 U/mL
AUC = 0.526
B
CA19-9 >129 U/mL
AUC = 0.633
C
Pathology
AUC = 0.657
D
BiliSeqV2/V3
AUC = 0.887
E
Pathology + BiliSeqV2/V3
AUC = 0.924
Sensitivity
1 - Specificity
0.00
0.20
0.40
0.60
0.80
1.00
F
Sensitivity
Specificity
NPV
PPV
Accuracy
CA19-9 ≥44 U/mL
CA19-9 >129 U/mL
Pathology
BiliSeqV2/V3
Pathology + BiliSeqV2/V3
59.1%
40.9%
33.3%
84.7%
92.8%
46.0%
85.7%
98.0%
92.6%
91.9%
51.8%
58.1%
66.4%
89.0%
94.5%
53.4%
75.0%
92.5%
89.5%
89.6%
52.7%
62.8%
70.4%
89.2%
92.3%
0%
25%
50%
75%
100%
G
Sensitivity
H
Specificity
I
NPV
J
PPV
K
Accuracy
Pathology + BiliSeqV2/V3
BiliSeqV2/V3
Pathology
CA19-9 >129 U/mL
CA19-9 ≥44 U/mL
< 0.001
0.004
0.058
1.000
0.146
0.012
0.039
0.021
0.019
0.349
0.047

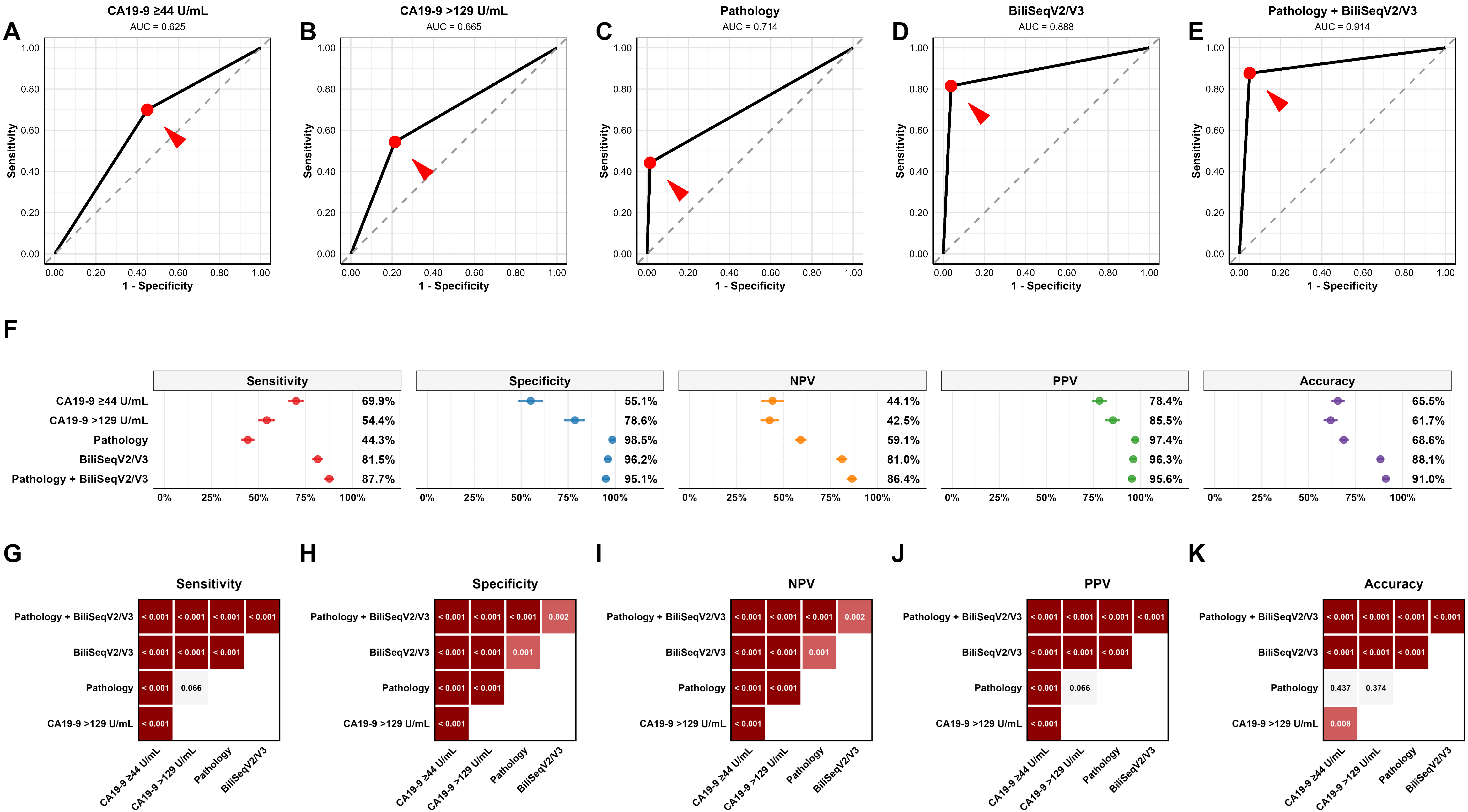

A
CA19-9 ≥44 U/mL
AUC = 0.625
B
CA19-9 >129 U/mL
AUC = 0.665
C
Pathology
AUC = 0.714
D
BiliSeqV2/V3
AUC = 0.888
E
Pathology + BiliSeqV2/V3
AUC = 0.914
Sensitivity
1 - Specificity
F
Sensitivity
Specificity
NPV
PPV
Accuracy
CA19-9 ≥44 U/mL
CA19-9 >129 U/mL
Pathology
BiliSeqV2/V3
Pathology + BiliSeqV2/V3
69.9%
54.4%
44.3%
81.5%
87.7%
55.1%
78.6%
98.5%
96.2%
95.1%
44.1%
42.5%
59.1%
81.0%
86.4%
78.4%
85.5%
97.4%
96.3%
95.6%
65.5%
61.7%
68.6%
88.1%
91.0%
G
Sensitivity
H
Specificity
I
NPV
J
PPV
K
Accuracy
Pathology + BiliSeqV2/V3
BiliSeqV2/V3
Pathology
CA19-9 >129 U/mL
CA19-9 ≥44 U/mL

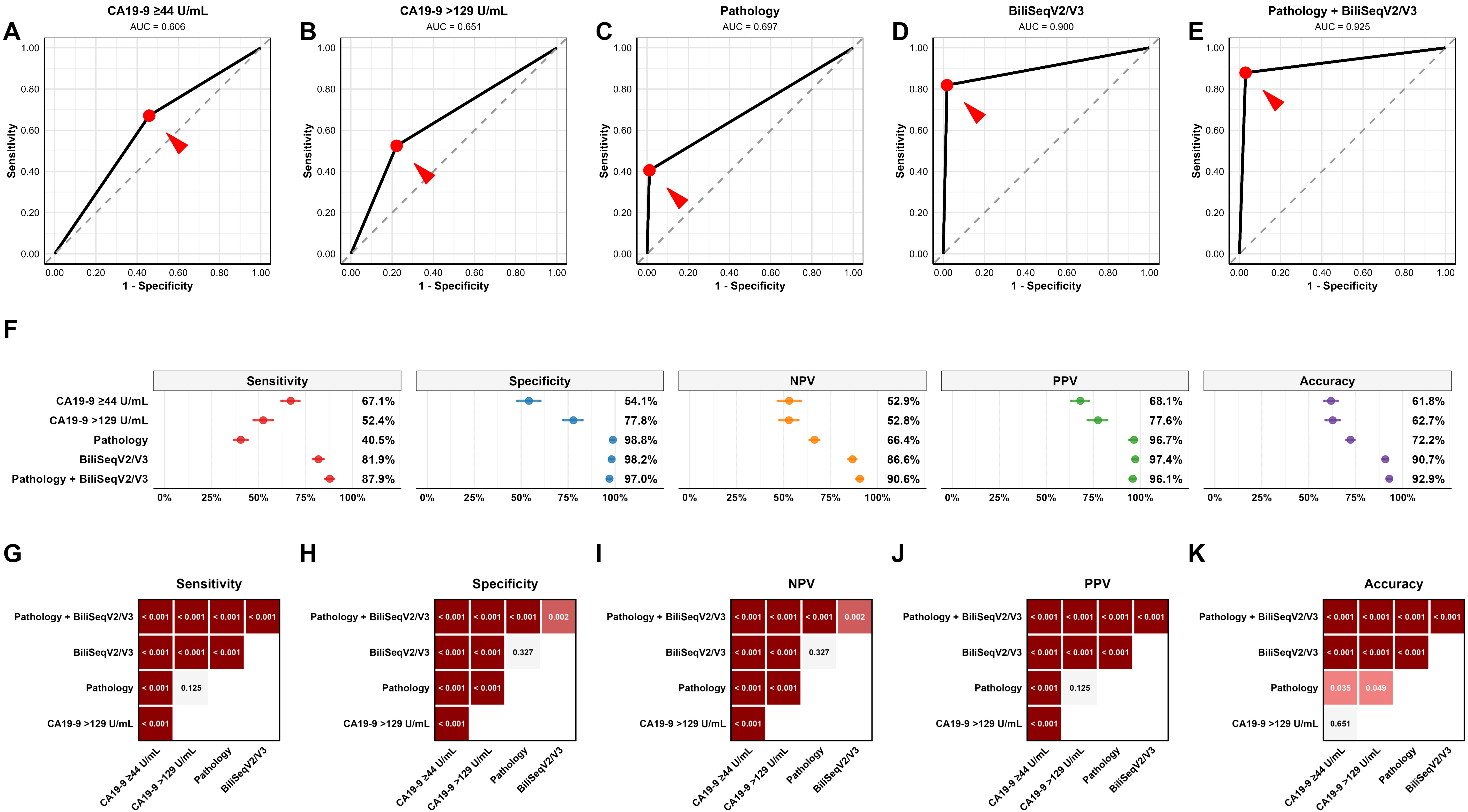
A
CA19-9 ≥44 U/mL
AUC = 0.606
B
CA19-9 >129 U/mL
AUC = 0.651
C
Pathology
AUC = 0.697
D
BiliSeqV2/V3
AUC = 0.900
E
Pathology + BiliSeqV2/V3
AUC = 0.925
Sensitivity
1 - Specificity
F
Sensitivity
Specificity
NPV
PPV
Accuracy
CA19-9 ≥44 U/mL
CA19-9 >129 U/mL
Pathology
BiliSeqV2/V3
Pathology + BiliSeqV2/V3
67.1% 52.4% 40.5% 81.9% 87.9%
54.1% 77.8% 98.8% 98.2% 97.0%
52.9% 52.8% 66.4% 86.6% 90.6%
68.1% 77.6% 96.7% 97.4% 96.1%
61.8% 62.7% 72.2% 90.7% 92.9%
G
H
I
J
K
Pathology + BiliSeqV2/V3: < 0.001 < 0.001 < 0.001 < 0.001 | < 0.001 < 0.001 < 0.001 0.002 | < 0.001 < 0.001 < 0.001 0.002 | < 0.001 < 0.001 < 0.001 < 0.001 | < 0.001 < 0.001 < 0.001 < 0.001
BiliSeqV2/V3: < 0.001 < 0.001 < 0.001 | < 0.001 < 0.001 0.327 | < 0.001 < 0.001 0.327 | < 0.001 < 0.001 < 0.001 | < 0.001 < 0.001 < 0.001
Pathology: < 0.001 0.125 | < 0.001 < 0.001 | < 0.001 < 0.001 | < 0.001 0.125 | 0.035 0.049
CA19-9 >129 U/mL: < 0.001 | < 0.001 | < 0.001 | < 0.001 | 0.651
CA19-9 ≥44 U/mL
CA19-9 >129 U/mL
Pathology
BiliSeqV2/V3
